# GWAS of peptic ulcer disease implicates *Helicobacter pylori* infection, other gastrointestinal disorders and depression

**DOI:** 10.1038/s41467-021-21280-7

**Published:** 2021-02-19

**Authors:** Yeda Wu, Graham K. Murray, Enda M. Byrne, Julia Sidorenko, Peter M. Visscher, Naomi R. Wray

**Affiliations:** 1 Institute for Molecular Bioscience, The University of Queensland, Brisbane, Australia; 2 Department of Psychiatry, University of Cambridge, Cambridge, UK; 3 Behavioural and Clinical Neuroscience Institute, University of Cambridge, Cambridge, UK; 4 Cambridgeshire and Peterborough NHS Foundation Trust, Cambridge, UK; 5 Queensland Brain Institute, The University of Queensland, Brisbane, Australia

**Keywords:** Genome-wide association studies, Irritable bowel syndrome, Gastro-oesophageal reflux disease, Peptic ulcers

## Abstract

Genetic factors are recognized to contribute to peptic ulcer disease (PUD) and other gastrointestinal diseases, such as gastro-oesophageal reflux disease (GORD), irritable bowel syndrome (IBS) and inflammatory bowel disease (IBD). Here, genome-wide association study (GWAS) analyses based on 456,327 UK Biobank (UKB) individuals identify 8 independent and significant loci for PUD at, or near, genes *MUC1*, *MUC6, FUT2*, *PSCA*, *ABO*, *CDX2, GAST* and *CCKBR*. There are previously established roles in susceptibility to *Helicobacter pylori* infection, response to counteract infection-related damage, gastric acid secretion or gastrointestinal motility for these genes. Only two associations have been previously reported for duodenal ulcer, here replicated trans-ancestrally. The results highlight the role of host genetic susceptibility to infection. Post-GWAS analyses for PUD, GORD, IBS and IBD add insights into relationships between these gastrointestinal diseases and their relationships with depression, a commonly comorbid disorder.

## Abstract (web summary)

Genetic factors contribute to peptic ulcer disease (PUD). Here, the authors perform a genome-wide association analysis on PUD in the UK Biobank, highlighting shared architecture with other gastrointestinal disorders and possible causal links with depression.

## Introduction

Gastrointestinal (GI) diseases are highly prevalent in western countries. They use substantial health care resources, have a heavy societal economic burden^1,2^, and impact the quality of life of those affected. GI disorders include peptic ulcer disease (PUD), gastro-esophageal reflux disease (GORD), irritable bowel syndrome (IBS), and inflammatory bowel disease (IBD), and some of these disorders are commonly reported as correlated with each other^3^. PUD, a common GI disorder, involves breaks (ulcers) in the inner lining of the digestive tract, usually located in the stomach or proximal duodenum. In GORD, the stomach contents leak back from the stomach into the esophagus^4^. IBS is a chronic functional disorder of the GI system. Patients with IBS often manifest abdominal pain and altered bowel habit, with either predominantly diarrhea, constipation, or both. IBD includes Crohn’s disease (CD) and ulcerative colitis (UC), which are chronic immune dysregulation disorders causing inflammation of the GI tract.

PUD is a complex disorder, for which *Helicobacter (H.) pylori* infection and the use of non-steroidal anti-inflammatory drugs (NSAIDs) are the main risk factors^5^. The development of infection-relevant PUD is recognized to be a multistep process, with contributions from both *H. pylori* infection and subsequent inflammation and damage of mucosa^5^. Eradicating *H. pylori* is effective for infection-relevant PUD treatment^5^. However, understanding the host factors influencing *H. pylori* infection and subsequent response could contribute to earlier risk identification and/or prevention, especially given the increasing antimicrobial resistance worldwide^5^. Moreover, clinical presentation of PUD that is not associated with *H. pylori* infection, nor with the use of NSAIDs, are now also imposing substantial diagnostic and therapeutic challenges^5,6^. Lifetime prevalence of PUD in the general population has been estimated to be about 5–10%^5^. GORD is a multifactorial disorder and is more common in individuals with obesity and hiatal hernia^7^. Lifetime risk estimates of GORD have a wide range (9–26%), with a sample size-weighted mean of 15%^8^. An increase in the prevalence of GORD since 1995 has been reported^8^. IBS, a common disorder with a population lifetime risk of 11% globally^9^, is also likely a multifactorial disease, where hypervigilance of the central nervous system, immune activation of the intestinal mucosa, microbiome, prior infections, and diet are all suspected to play a role^10^. IBD is associated with many lifestyle risk factors, particularly smoking^11^, and lifetime risk for IBD is around 0.3% in most countries of Europe^12^. The genetic contributions to PUD, GORD, IBS and IBD have been well-recognized^13–16^, and well-powered genome-wide association studies (GWASs) have identified >200 approximately independent susceptibility loci associated with IBD^17^. These loci implicate pathways such as autophagy and the IL-17/IL-23 axis and provide insights into IBD pathogenesis^17^. IBD has been extensively studied through the GWAS paradigm^17^ but to a lesser extent for PUD^18^, GORD^19,20^, and IBS^21–23^. Notably, the only GWAS to date for duodenal ulcer was in a Japanese ancestry cohort^18^.

Our primary focus was to identify genetic susceptibility factors for PUD by conducting a GWAS using data from the UK Biobank (UKB). Given the relationship between PUD and GORD (both acid-related disorders), the comorbidity of GORD and IBS^3^ and the much increased sample sizes afforded by the UKB, we also present GWAS results for GORD and IBS and investigate the shared genetic architecture between them. Inflammation of the gut is a key characteristic of IBD but not IBS^24^, and hence differences between IBD and IBS from a genetic perspective are expected. Here, we take the opportunity to evaluate formally the genetic relationship between IBD and the more common disorders PUD, GORD, and IBS. Given increasing evidence for the importance of bidirectional signaling between the brain and the gut^25–28^, possibly contributing to observational associations between depression and PUD^29^, GORD^30^, IBS^31^, and IBD^32^, we explore the potential causal relationships between major depression (MD) and the four disorders using Mendelian randomization (MR).

Here, we identify eight independent and significant single nucleotide polymorphisms (SNPs) for PUD and the results highlight the role of host genetic susceptibility to infection, acid secretion, and gastric motility. We also identify a total of 11 independent and significant SNPs previously unreported for GORD and IBS. We show genetic similarity across PUD, GORD, and IBS and between these GI diseases with psychiatric disorders. Post-GWAS analyses find a link between PUD, GORD, and IBS with the nervous system. We find a significant relationship between PUD, GORD, IBS, and major depression through observational and MR analyses. Taken together, our results expand our understanding of the role of genetics in gastrointestinal diseases and add insights into relationships between these gastrointestinal diseases and their relationships with MD.

## Results

The workflow for our study is given in Supplementary Fig. 1.

### Prevalence and comorbidity

**Fig. 1:**
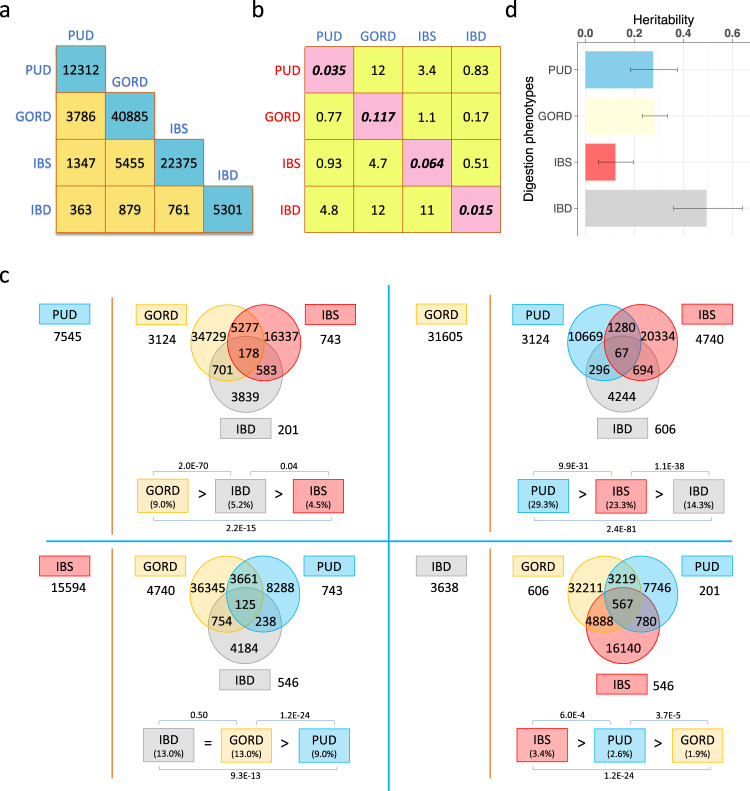
Comorbidity analyses in unrelated European individuals and heritability estimation based on full-sibling relative risk for PUD, GORD, IBS, and IBD. **a** The number of unrelated individuals with each diagnosis (cyan boxes) and the number of overlapped individuals between each pair of PUD, GORD, IBS, and IBD cases (yellow boxes). These differ from Table 1 because only unrelated individuals are considered here. **b** Cells represent ratio of the odds of disease cases from each column in those with disease from each row and the odds of each row disease cases in unrelated European-ancestry individuals. The diagonal elements are the sample risk rates and differ from “*N* case/(*N* case + *N* control)” in Table 1 because only unrelated individuals are included here. **c** Competitive comorbidity analyses for each of PUD, GORD, IBS, and IBD disease (defined, in turn, as index disease) to test among the other three diseases which disease is more prone to be comorbid with the index disease. The disease on the left of the red dashed line is the index disease and the number shows the number of cases without any comorbidity. The corresponding Venn diagram shows the number of individuals with recorded or self-report diagnosis with at least one of the other three diseases. The numbers outside of the Venn diagrams are the numbers of individuals with diagnosis of both the index disease and each of the other three diseases in turn, after removing the overlapped individuals for these three diseases. At the bottom of each Venn diagram is the proportion of the index disease cases in the other three diseases respectively, compared in pairs using a two-proportion *Z* test to test which disease is more prone to be comorbid with the index disease. **d** Heritability estimates and 95% confidence interval for PUD, GORD, IBS, and IBD based on full-sibling relative risk. See the Supplementary Note 1 for an example explaining **b**, **c**.

Based on disease-diagnosis (self-reported, hospital admission, primary care, and death register records) in the UKB, four case–control digestion disorder datasets were identified (Table 1), with prevalences for PUD, GORD, IBS and IBD of 3.7%, 12.0%, 6.5%, and 1.5%, respectively (Table 1). We note that the UKB prevalence rates are higher for IBD than the 0.3% aforementioned lifetime risk^12^. This may reflect ascertainment biases or misdiagnosis within the UKB. Disease statistics (Table 1) and comorbidity analyses (Fig. 1) help describe the UKB phenotypes. The male/female odds ratio for being a PUD case is 1.66 while for IBS it is 0.42 (Table 1), consistent with PUD being more common in men and IBS more common in women. To allow readers to interpret the phenotype data that underpin our analyses, we describe in detail the disorder co-occurrences present in UKB participant records (Fig. 1 and Supplementary Note 1), which may reflect, in part, the natural course of the symptom presentations and/or misdiagnosis. For example, it is well recognized that IBD patients may have symptoms that provide a PUD or GORD diagnosis prior to, or as part of, the endoscopies that generate an IBD diagnosis and/or monitor the disease progress. Moreover, GORD can occur as a consequence of treatment of *H. pylori* for PUD as *H. pylori* infection can reduce gastric acidity^33^. Here, while rates of PUD and IBD in those with GORD were significantly lower than the rate of GORD cases in the UKB as a whole, those with PUD or IBD were significantly more likely to also have GORD. For each of the PUD, GORD, IBS, and IBD (defined as the index disease), competitive comorbidity analyses tested, among the other three diseases, which disease is more prone to be comorbid with the index disease. We found that PUD is more prone to be comorbid with GORD while IBS is more likely to be comorbid with IBD (Fig. 1c).

**Table 1:** Full-sibling relative risk and heritability estimation for PUD, GORD, IBS, and IBD.

	Digestion phenotypes			
	PUD	GORD	IBS	IBD
*N* case:*N* control*	16666:439661	54854:401473	29524:426803	7045:449282
*N* case/(*N* case + *N* control)	0.037	0.120	0.065	0.015
No. of male case:No. of female case	9641:702s5	24841:30013	7981:21543	3432:3613
No. of male control:No. of female control	199156:240505	183956:217517	200816:225987	205365:243917
Odds of being male case/Odds of being female case	0.048/0.029	0.135/0.138	0.040/0.095	0.017/0.015
Male:female odds ratio for being case	1.66	0.98	0.42	1.13
No. of full-sibling pairs where both proband and full-sibling are cases	104	940	236	38
No. of full-sibling pairs where only the proband is a case	1460	4530	2682	630
Full-sibling relative risk (95% confidence interval)	1.82 (1.51–2.19)	1.43 (1.35–1.52)	1.25 (1.11–1.41)	3.68 (2.70–5.02)
Heritability (95% confidence interval)^†^	0.28 (0.18–0.37)	0.28 (0.23–0.33)	0.12 (0.05–0.19)	0.49 (0.36–0.64)

^*^The number for the cases and controls of each digestion phenotypes are from the whole UK Biobank individuals with European ancestry, i.e., related individuals are included.

^†^The corresponding lower and upper values of 95% confidence interval (CI) for risk in full-sibling were used to calculate the 95% CI for heritability estimation.

### Full-sibling risk and heritability estimation

Using SNP-based estimates of coefficients of genetic relationship between individuals in the UKB, we estimated the full-sibling relative risk for each of the PUD, GORD, IBS, and IBD and the heritability of liability, with the assumption that the increased risk in relatives only reflect shared genetic factors (Table 1 and Fig. 1d). The estimated heritabilities for PUD, GORD, IBS, and IBD were 0.28 (95% CI: 0.18–0.37), 0.28 (95% CI: 0.23–0.33), 0.12 (95% CI: 0.05–0.19), and 0.49 (95% CI: 0.36–0.64), respectively, all significantly different from zero.

### GWAS

Genome-wide association analyses were conducted for five digestion phenotypes, the four disease-diagnosis traits (PUD, GORD, IBS, and IBD, see “**Methods**” section) and one trait that combined the disease-diagnosis and taking of corresponding medications and treatments (Supplementary Tables 1, 2). In clinical practice, medications for PUD also have a therapeutic effect on GORD, hence we generated the PG_+_M phenotype—a combination of disease-diagnosis of GORD and/or PUD and/or corresponding medications and treatments. We tested for association between 8,546,065 DNA variants and each of the five digestion phenotypes (PUD, GORD, PG_+_M, IBS, and IBD) in 456,327 UKB participants. A total of 66 within-trait (61 across-trait) independent variants were genome-wide significant (*P* < 5.0E−8) for the five digestion phenotypes analysed, of which 8 were associated with PUD, 6 with GORD, 19 with PG_+_M, 2 with IBS, and 31 with IBD. Two PUD-associated SNPs have been previously linked to PUD but in a Japanese cohort^18^. For GORD, a recent GWAS study^20^ which used an earlier release of UKB data plus meta-analysis with other studies reported 25 genome-wide significant SNPs. Conditional analyses of our GORD and PG_+_M association results on SNPs previously reported as genome-wide significantly associated gastroesophageal reflux^20^ using GCTA-COJO^34,35^ found that three SNPs remain significant for GORD and six SNPs remain significant for PG_+_M. Given the aim of our study, Tables 2–3 list the 19 previously unreported genome-wide significant SNPs for PUD, GORD, PG_+_M, and IBS. SNPs associated with IBD are in Supplementary Table 3; we note that 28 of the 31 SNPs have been previously linked with inflammatory bowel diseases (despite concerns of the high prevalence of IBD in the UKB). The three GORD-associated SNPs and 13 PG_+_M-associated SNPs that have been reported^20^ are listed in Supplementary Table 4. The GERA cohort data^36,37^ were available as a replication sample for PUD (1004 cases, 60,843 controls) and IBS (3359 cases and 58,488 controls), albeit with limited power (~20% for each SNP, Supplementary Table 5). Six of the eight genome-wide significant SNPs for UKB PUD exist and one for UKB IBS in GERA and all have very similar effect size estimates as those in UKB, but only rs681343 was formally significant (*P* = 5.0E−4 < 0.05/8, a replication rate consistent with the power calculations) (Supplementary Table 5).

**Table 2:** Genome-wide significant SNPs associated with PUD in the UK Biobank.

Digestion phenotypes	SNP*	CHR.	BP	A1/A2	A1 frequency	OR^†^	*P*	Nearby genes^‡^	Digestive diseases pleiotropy^§^	Mental health pleiotropy^§^
PUD	rs681343	19	49206462	C/T	0.49	0.92	1.9E−15	*FUT2*	Diarrheal disease, Crohn’s disease, IBD, and Gallstone disease	–
	rs2976388	8	143760256	G/A	0.58	1.09	1.8E−14	*PSCA*	Duodenal ulcer, gastric atrophy, and gastric cancer	–
	rs10500661	11	6273744	T/C	0.80	0.90	4.1E−14	*CCKBR*	–	–
	rs147048677	1	155161794	C/T	0.94	0.86	9.0E−12	*MUC1*	–	–
	rs78459074	11	1029905	A/G	0.89	1.12	2.6E−10	*MUC6*	–	–
	rs34074411	17	39867248	C/T	0.56	0.93	2.6E−10	*GAST*	–	–
	rs687621	9	136137065	A/G	0.68	1.08	1.3E−09	*ABO*	Duodenal ulcer, Gastric cancer, and pancreatic cancer	–
	rs9581957	13	28557889	C/T	0.68	0.93	3.6E−09	*CDX2*	–	–

*Locus zoom plot for SNPs are in Supplementary Fig. 4.

^†^Odds ratio (OR) is for risk of A1 allele compared to A2 allele.

^‡^We note that we do not have direct evidence to support the nearby genes as causal genes, except when linked to gene expression (see Summary data-based Mendelian randomization analysis at the links to gene expression, eQTLs, and mQTLs section).

^§^We only annotated SNPs if there are SNPs reported associated with either mental health-related traits or digestive diseases from GWAS Catalog in linkage disequilibrium with our UKB digestion SNPs (see “Methods” section and Supplementary Data 1 for detailed description).

IBD inflammatory bowel diseases.

**Table 3:** Genome-wide significant SNPs associated with GORD, PG_+_M, and IBS in the UK Biobank.

Digestion phenotypes	SNP*	CHR.	BP	A1/A2	A1 frequency	OR^†^	*P*	Nearby genes^‡^	Digestive diseases pleiotropy^§^	Mental health pleiotropy^§^
GORD	rs77968610	12	78532859	T/C	0.88	0.95	2.8E−08	*NAV3*	–	–
	rs32546	5	13205087	C/T	0.60	1.04	3.5E−08	*–*	–	–
	rs10891491	11	112898216	C/T	0.89	0.95	4.1E−08	*NCAM1*	–	Depressive symptoms, depressed affect
PG_+_M	rs12532051	7	147806981	C/G	0.96	1.08	1.2E−09	*CNTNAP2*	–	–
	rs9800013	5	542023	A/G	0.77	0.96	2.9E−09	*–*	UC	–
	rs149140438	22	32277882	A/C	0.98	0.90	9.9E−09	*DEPDC5*	–	–
	rs72915655	2	194001113	T/C	0.83	1.04	1.1E−08	*–*	–	ASD, Anorexia nervosa, SCZ, etc.
	rs3863241	8	73890335	C/T	0.47	0.97	1.9E−08	*–*	–	–
	14:103333187	14	103333187	AAC/A	0.76	0.97	4.3E−08	*TRAF3*	–	–
IBS	rs7947502	11	112909396	C/T	0.41	1.05	2.5E−08	*NCAM1*	–	Depressive symptoms, depressed affect
	rs2523599	6	31241092	C/G	0.39	0.95	4.4E−08	*HLA-C*	#	#

^*^Locus zoom plot for SNPs are in Supplementary Figs. 5–7. Supplementary Table 4 lists genome-wide significant SNPs associated with GORD and PG_+_M that have been previously reported to be associated with gastroesophageal reflux^20^.

^†^Odds ratio (OR) is for risk of A1 allele compared to A2 allele.

^‡^We note that we do not have direct evidence to support the nearby genes as causal genes, except when linked to gene expression (see Summary data-based Mendelian randomization analysis at the Links to gene expression, eQTLs, and mQTLs section).

^§^We only annotated SNPs if there are SNPs reported associated with either mental health-related traits or digestive diseases from GWAS Catalog in linkage disequilibrium with our UKB digestion SNPs (see “Methods” section and Supplementary Data 1 for detailed description). “#” represents that SNP is within MHC region. For SNPs within MHC region see Supplementary Data 1 for details given the complexity of MHC region.

*ASD* Autism spectrum disorder, *SCZ* Schizophrenia, *UC* Ulcerative colitis.

**Fig. 2:**
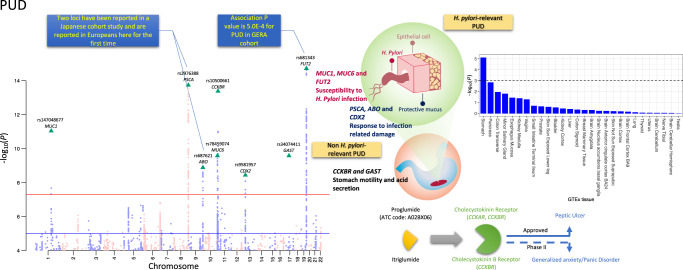
Manhattan plot of peptic ulcer disease (PUD) for SNPs associated *P* < 1.0E−4 from BOLT-LMM association test. SNPs highlighted with green triangles are independent loci with *P* < 5.0E−8. rs2976388 and rs687621 are the only two loci associated with duodenal ulcer in a Japanese cohort^18^ and rs681343 showed statistically significant association in GERA PUD GWAS, as annotated in the blue box. SNPs on odd/even chromosomes are presented in mauve/pink. Schematic diagram on the right side represents the reported biological evidence supporting involvement in peptic ulcers of genes physically located near PUD-associated loci (noting that we do not have direct evidence to link the associated SNPs with the genes). *MUC1*^76^, *MUC6*^77^, and *FUT2*^73^ have been linked to susceptibility to *H*. *pylori* infection and *PSCA* and *ABO* have been proposed to be associated with subsequent response after infection^18^. Induced/enhanced *CDX2* expression as a result of *H. pylori* infection of gastric epithelial cell lines has been observed^79^. *GAST* encodes gastrin, which is a hormone whose main function is to stimulate secretion of hydrochloric acid by the gastric mucosa. *CCKBR* encodes cholecystokinin receptor which mediates a therapeutic effect for peptic ulcer treatment by reducing acid secretion and inhibiting gastrointestinal motility. The cholecystokinin receptor is also an effect-mediating target of itriglumide on phase II clinical trial for anxiety and panic disorder^81^. Using the GENE2FUNC of FUMA pipeline^38^, we found that the eight annotated genes are highly overexpressed in human stomach tissue from GTEx 8th version data, as shown in the bar plot on the right side with data presented in Supplementary Data 2. The dashed line represents Bonferroni corrected significance at −log_10_(0.05/54).

**Fig. 3:**
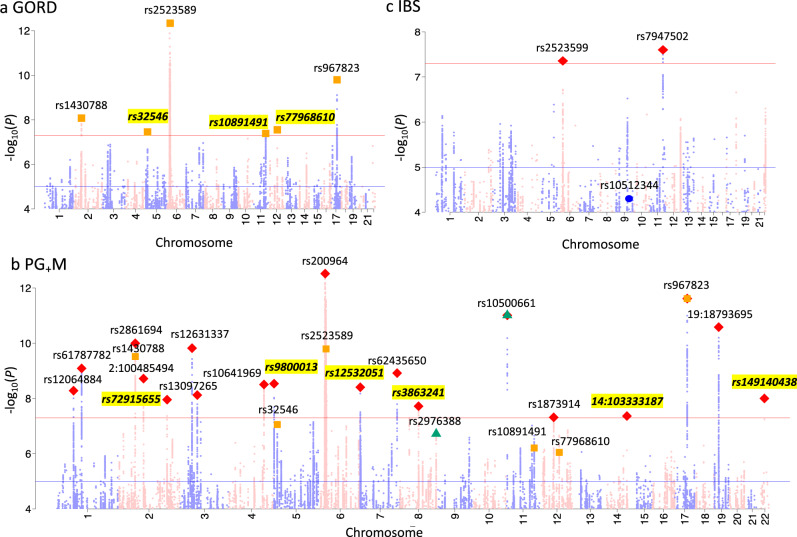
Manhattan plot of gastroesophageal reflux disease (GORD), peptic ulcer disease, GORD and corresponding medications (PG_+_M) and irritable bowel syndrome (IBS) for SNPs associated *P* < 1.0E−4 from BOLT-LMM association test. **a** GORD: SNPs highlighted with orange squares are genome-wide statistically significant (*P* < 5.0E−8) independent loci, which correspond to the orange squares in **b**. SNPs highlighted with yellow represent loci that have not previously been reported to be associated with GORD. **b** PG_+_M: SNPs highlighted with red diamond are independent loci with *P* < 5.0E−8. Only two of eight SNPs associated with peptic ulcer disease (highlighted with green triangles) are with *P* < 1.0E−5 in PG_+_M and all the SNPs associated with GORD (highlighted with orange squares) have *P* < 1.0E−5 in PG_+_M. SNPs highlighted with yellow represent loci that have not previously been reported to be associated with GORD. **c** IBS: SNPs highlighted with red diamond are independent loci with *P* < 5.0E−8. The blue dot is for rs10512344 that has been reported associated with female IBS in UKB previously released data^23^. The IBD Manhattan plot is found in Supplementary Fig. 2.

Many of the PUD SNPs associations are previously unreported even when checking published results for any digestive diseases (Table 2). There are eight PUD-associated SNPs, which by physical distance (<22 kb) could implicate *MUC1*, *MUC6*, *FUT2*, *PSCA*, *ABO*, *CDX2*, *GAST*, and *CCKBR*, as annotated in Fig. 2. Pathway analysis using the GENE2FUNC of the FUMA pipeline^38^ showed that these genes are highly overexpressed in human stomach tissue. Detail on these PUD results are found in the discussion section. For IBS, a previous study of UKB data (earlier phenotype release) reported rs10512344 as the only SNP genome-wide significantly associated (*P* = 3.6E−8) with self-report IBS, and in females only^23^. The *P* value for this SNP in our analyses is 5.0E−05 (*P* = 4.4E−07 in females, i.e., less associated than in the published study despite sample overlap). Given that they used a self-report phenotype while we used a combination of three resources, we regenerated three subgroup phenotypes (self-report only, hospital admission only, and primary care only) for IBS and repeated GWAS analyses for the three phenotypes and the results were used for further analyses (Supplementary Note 2). We also conducted similar subgroup phenotypes GWAS for GORD and PUD. Figure 2 shows Manhattan plots for PUD. Figure 3 shows Manhattan plots for GORD, PG_+_M, and IBS and Supplementary Fig. 2 shows Manhattan plots for IBD. Quantile–Quantile (Q–Q) plots of all the variants analysed in UKB are provided in Supplementary Fig. 3 for the five phenotypes. Regional visualization plots of the 66 independent variants are in Supplementary Figs. 4–8. Detailed pleiotropy results derived from the GWAS Catalog^39^ are provided in Supplementary Data 1. The 54 GTEx tissue specific enrichment results for eight genes in Fig. 2 are in Supplementary Data 2. In sensitivity analysis GWAS, phenotypes for each of PUD, GORD, IBS, and IBD were regenerated by excluding individuals with more than one GI diagnosis among these four disorders from the corresponding original phenotype cases (Supplementary Note 3).

### SNP-based heritabilities and genetic correlations

**Fig. 4:**
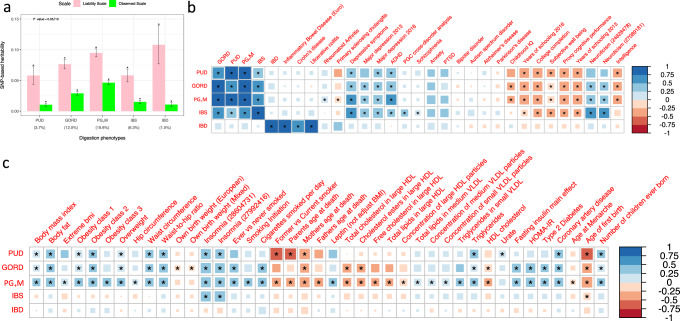
SNP-based heritability and genetic correlation analyses for the five digestion phenotypes from LD score regression analyses. **a** SNP-based heritability of the five digestion phenotypes both on the observed and liability scales. The transformation to the liability scale uses the UKB sample risk, i.e., the proportion cases in the UKB cohort, as the population lifetime risk; the sample risk percentage of whole UKB European ancestry is shown below the *x*-axis in parentheses. The error bars represent 95% confidence interval for the estimated SNP-based heritability. “*” represents that the SNP-based heritability *P* value remain significant after Bonferroni correction (*P* < 0.05/10). **b**, **c** Results for genetic correlation within-digestion phenotypes and between digestion phenotypes and the nine psychiatric and neurological disorder traits are in **b**, while genetic correlation results between digestion phenotypes and traits from LD Hub that had a significant correlation with at least one of the digestion phenotypes are provided in **b**, **c**. “*” represent that genetic correlation estimates are still significant after Bonferroni correction (*P* < 0.05/((5 + 9 + 258) * 5)).

We estimated the proportion of variance in trait liability attributable to genome-wide common SNPs (i.e., SNP-based heritability, $h_{{\rm{SNP}}}^2$) using Linkage Disequilibrium Score regression (LDSC)^40^. $h_{{\rm{SNP}}}^2$ estimates on the liability scale were: PUD 0.06 (SE = 0.007), GORD 0.08 (SE = 0.004), PG_+_M 0.09 (SE = 0.003), IBS 0.06 (SE = 0.005), and IBD 0.11 (SE = 0.016) (Fig. 4a and Supplementary Table 6), all significantly different from zero. The SNP-based genetic correlation (*r*_g_) between PUD and GORD is 0.65 (SE = 0.05, *P* = 4.9E−36, phenotypic correlation (*r*_p_) = 0.11), similar to the *r*_g_ estimate for GORD and IBS (0.65, SE = 0.05, *P* = 1.1E−46, *r*_p_ = 0.10). The *r*_g_ between PUD and IBS is 0.49 (SE = 0.08, *P* = 2.0E-10, *r*_p_ = 0.03) (Supplementary Table 7), while the *r*_g_ between IBD and each of PUD, GORD, PG_+_M, and IBS are not statistically significantly different from zero after Bonferroni correction (Fig. 4b). In sensitivity analyses, all individuals with more than one GI diagnosis were excluded (Supplementary Fig. 9a); the $h_{{\rm{SNP}}}^2$ estimates were lower but still significantly different from zero (Supplementary Table 6 and Supplementary Fig. 9b), while the *r*_g_ between PUD, GORD, and IBS remain significant (all *r*_g_ > 0.25) and none showed statistically significant *r*_g_ with IBD (Supplementary Table 7 and Supplementary Fig. 9c_1_). Detailed results of sensitivity analyses are discussed in Supplementary Note 3 with the corresponding data presented in Supplementary Tables 6–7 and Supplementary Fig. 9. We also investigated $h_{{\rm{SNP}}}^2$ and *r*_g_ for the three subgroup phenotypes (mentioned above) of each of PUD, GORD, and IBS, and found them to show significant $h_{{\rm{SNP}}}^2$ and high genetic correlation (see Supplementary Fig. 10 and Supplementary Tables 8–9).

The *r*_g_ between each of the five digestion phenotypes and the 258 traits from LD Hub^41^ plus nine psychiatric^42–48^ and neurological traits^49,50^ (Supplementary Table 10 and Supplementary Data 3, respectively) included 26, 37, 53, 16, and 3 significant correlations for PUD, GORD, PG_+_M, IBS, and IBD, respectively, after Bonferroni correction (*P* < 3.7E−5). Figure 4b, c show the *r*_g_ between each of the five phenotypes and statistically significant correlations from the 267 traits. We summarize these results briefly. First, we observed significant positive *r*_g_ between four digestion phenotypes (PUD, GORD, PG_+_M, and IBS) and depressive symptoms^51^, major depression (MD)^48^, attention deficit hyperactivity disorder (ADHD)^42^, neuroticism^51^, and insomnia^52,53^. However, there was no statistically significant *r*_g_ between IBD and these traits. Second, PUD, GORD, and PG_+_M have significant positive *r*_g_ with body mass index (BMI)^54^, body fat-related traits^55^ and coronary artery disease^56^. GORD and PG_+_M are also genetically correlated with type 2 diabetes^57^ in a positive direction. Third, the common variant genetic architecture of PUD, GORD, PG_+_M, and IBS are positively correlated with educational attainment-related traits^58^. In sensitivity analyses, all individuals with more than one GI diagnosis were excluded and the results are similar as above. Detailed results of sensitivity analysis are discussed in Supplementary Note 3 with the corresponding data presented in Supplementary Table 11, Supplementary Fig. 9, and Supplementary Data 4.

### Links to gene expression, eQTLs, and mQTLs

**Fig. 5:**
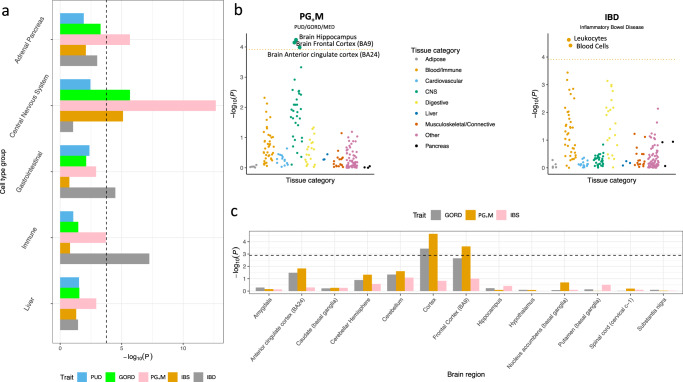
Analyses of partitioning SNP-based heritability by different annotations. **a** SNP-based heritability enrichment analysis for each digestion phenotype partitioned by cell type groups annotated by histone marks. The dashed line represents the Bonferroni corrected significance threshold (0.05/(53 * 5 + 5 * 5)). **b** SNP-based heritability enrichment analysis for PG_+_M and IBD partitioned by cell types annotated using cell-type specific gene expression data given the results from **a**. The dotted lines represent the Bonferroni correction threshold (*P* < 0.05/(205*2)). **c** SNP-based heritability enrichment analysis for GORD, PG_+_M, and IBS partitioned by cell types annotated using fine-scale GTEx brain gene expression data from 13 brain regions selected given the central nervous system enrichment results of these three phenotypes from **a**. The dashed line represents the Bonferroni correction threshold (*P* < 0.05/(13*3)).

We used partitioned SNP-based heritability analyses to determine if any genomic annotations showed enrichment of $h_{{\rm{SNP}}}^2$ relative to the null hypothesis that SNP-based heritability is partitioned proportional to the number of SNPs in an annotated set (see “Methods” section). After Bonferroni correction, GORD, PG_+_M, and IBS showed significant enrichment of $h_{{\rm{SNP}}}^2$ in conserved regions and $h_{{\rm{SNP}}}^2$ enrichment for IBD was in the super enhancer category (Supplementary Fig. 11 and Supplementary Table 12). In analyses based on SNP annotations derived from cell-type histone-mark data (Fig. 5a and Supplementary Table 13), IBD showed significant $h_{{\rm{SNP}}}^2$ enrichment in immune and gastrointestinal cell-type groups, while GORD, PG_+_M, and IBS showed enrichment in the CNS cell-type. Based on cell-type specific SNP annotations^59^ derived from gene expression data of 205 different tissues (53 from GTEx^60^ and 152 from Franke lab^61^), PG_+_M showed significantly enriched association with genes expressed in the hippocampus, frontal cortex (Brodmann Area, BA9) and anterior cingulate cortex (BA24) of the brain and IBD showed enriched associations in leukocytes (Fig. 5b and Supplementary Table 14). Given these significant results for GORD, PG_+_M, and IBS we conducted the same analyses using the fine-scale GTEx brain gene expression data which includes data from 13 brain regions (albeit smaller sample size)^60^. GWAS associations for PG_+_M were consistently enriched in the frontal cortex (BA9) (Fig. 5c and Supplementary Table 15). We conducted sensitivity analyses to investigate if these results are mediated by educational attainment (EA)^62^, BMI^55^, and smoking-related traits^63^. The results remain significant after conditioning the PG_+_M GWAS results on the GWAS results of EA, BMI and smoking using mtCOJO^37^ (Supplementary Tables 13–15). We also investigated whether associations between SNPs and the five digestion phenotypes were consistent with mediation through gene expression using the Summary data-based Mendelian randomization method, SMR^64^. SMR combines genome-wide significant SNP-gene expression associations (i.e., eQTLs) with the SNP-trait association results. Significant SMR associations provide the best statistical evidence given available data that the trait associated SNP could be a causal SNP through its action on gene expression. A total of five unique genes for which expression is significantly associated with three digestion phenotypes, including two genes for PUD (*PSCA* and *FUT2*), one gene for PG_+_M (*SUOX*), and one gene for IBD (*RP11-129J12.2* and *RPS23P10*), were identified (Supplementary Table 16). The statistical framework of SMR^64^ can be applied to mQTL (genome-wide significant SNP-methylation association) data to identify putative methylation-trait association. Hence, we repeated SMR analyses for PUD using blood mQTL data from McRae et al.^65^. Among the mQTL SMR results (Supplementary Table 17), three DNA methylation probes (cg01656853, cg08873673, and cg04660111), located in promoter region of *FUT2*, are associated with PUD (Supplementary Fig. 12). From Roadmap epigenomics annotation^66^, there is a digestive tissue specific active enhancer for *FUT2*, as shown in Supplementary Fig. 12.

### Gene-based and gene-set enrichment analyses

While individual SNP associations can implicate relevance of specific genes, given gene-specific genetic architectures putative roles of genes can also be identified by gene-based tests that combine the SNP-associations into genic annotations. We used MAGMA^67^ software to identify genes significantly (*P* < 2.7E−6) associated with each of the five digestion phenotypes, identifying: 29 for PUD, 112 for GORD, 157 for PG_+_M, 14 for IBS, and 97 for IBD (Supplementary Data 5). For gene-set enrichment analysis applied to gene-based summary statistics identified 11 gene ontology (GO) sets as significant for PG_+_M and six for IBD. The top enriched gene set for PG_+_M is “GO: POSTSYNAPSE” while that for IBD is “GO MHC CLASS II PROTEIN COMPLEX” (Supplementary Table 18).

### Comorbidity and MR with depression

**Fig. 6:**
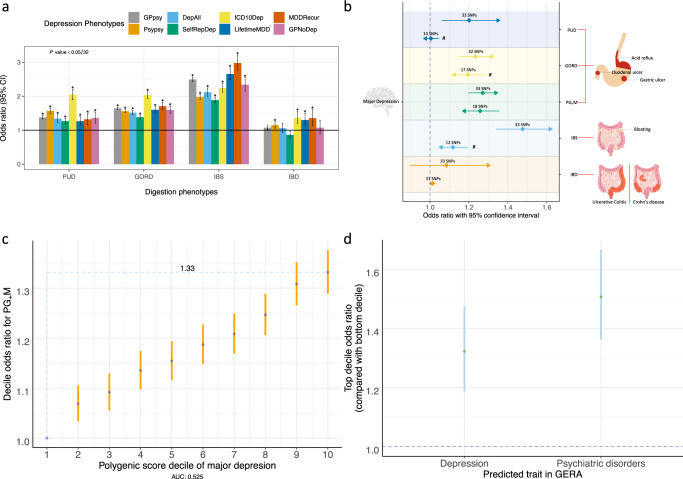
Comorbidity, Mendelian randomisation and polygenic risk score analyses with depression. **a** Comorbidity relationship between eight depression phenotypes^68^ and each of the PUD, GORD, IBS, and IBD. Odds ratio and 95% CI were calculated based on the 2 by 2 contingency table for number of cases and controls of each of the 32 digestion-depression phenotype pairs (Supplementary Tables 19 and 20). The definitions of the eight depression phenotypes are in the Methods section. “*” represents the *P* value for each digestion and depression pair that remain significant after Bonferroni correction (*P* < 0.05/32). **b** Mendelian randomization (MR) results between major depression (MD) and five digestion phenotypes. The left *y* axis is for MD while the right *y* axis is for the five digestion phenotypes. The arrow for each horizontal line represents the direction from exposure trait to outcome trait relative to the *y*-axis labels. OR and 95% CI are represented as diamond and horizontal lines taking values from *x* axis. Each digestive phenotype corresponds to two horizontal lines. “***R***” on the right side of the horizontal line represents relaxation of significance threshold of SNP associations to obtain more SNP genetic instruments (Supplementary Table 21). The number of the SNP instruments used in analyses are shown above the diamond. The common pathological characteristics or symptoms for these phenotype-related diseases are shown on the right side (noting that pathological characteristics or symptoms are not limited to these locations). **c** Decile of major depression polygenic score predicts PG_+_M. Odds ratio (OR) and 95% confidence intervals (CI, blue dots and orange bars) relative to decile 1 were estimated using logistic regression. The blue dashed lines shows the OR for the highest decile, OR of 1.33 for PG_+_M related disorders. **d** OR and 95% CIs (green dots and blue bars) for top decile of PG_+_M polygenic score predicting depression and psychiatric disorders in the independent GERA cohort^36^.

Given observational associations between depression and PUD^29^, GORD^30^, IBS^31^, and IBD^32^, we used the eight UKB depression phenotypes identified by Cai et al.^68^, together with our four digestion diagnoses, to test whether each of the 32 depression-digestion phenotypes pairs show significant lifetime comorbidity relationships. Cai et al.^68^ argued that some of the clinically derived depression phenotypes were more specific to major depressive disorder than self-report depression, and that biological interpretation should focus on analyses using clinically derived phenotypes. We were interested to know if the relationship between depression and common GI disorders depended on these different depression definitions. All eight depression phenotypes showed statistically significant comorbidity relationship with each of PUD, GORD, and IBS (Supplementary Tables 19, 20 and Fig. 6a). For IBD, the highest statistically significant ORs were for the more severe depression definitions of electronic medical record depression (ICD10Dep), DSM-V clinical guideline-defined major depressive disorder and recurrence (abbreviation: LifetimeMDD and MDDRecur) (Fig. 6a). Given the comorbidities of the digestion phenotypes with depression, we tested for statistical evidence of a putative causal association between MD and each of the five digestion phenotypes using Generalized Summary-data-based MR (GSMR)^37^; we also tested for reverse causality (Supplementary Table 21 and Supplementary Figs. 13, 14). For the relationship between MD and IBD, GSMR estimates were not statistically significant either in forward direction (the effect of MD on IBD) or the reverse direction (the effect of IBD on MD), despite being well-powered to identify associations. We found a unidirectional effect between MD and PUD, 1.20-fold risk for PUD per standard deviation (SD) in liability to MD. The reverse direction (PUD as exposure and MD as outcome) had a small point estimate and is not statistically significant, but we note that we relaxed the association significance threshold to obtain more PUD genetic instruments (8 SNPs to 13 SNPs). When we repeated the analyses (PUD as exposure and MD as outcome) using the eight genome-wide significant SNPs, the result remained statistically non-significant, suggesting that MD is putatively causal for PUD. This analysis should be revisited when GWAS sample size for PUD increases identifying more genome-wide significant SNP instruments. The effect of MD on GORD and IBS showed statistically significant estimates of 1.23-fold and 1.48-fold respectively increase per SD in liability to MD (Fig. 6b). The point estimates for the reverse causality analyses were smaller (but statistically significant), and again these analyses should be revisited in when more genome-wide significant SNPs are identified (Fig. 6b and Supplementary Table 21). We observed bidirectional statistically significant results between MD and PG_+_M, i.e., 1.27-fold increased risk for PG_+_M per SD) in liability to MD (*P* = 2.5E−15), and 1.26-fold increased risk for MD per SD in liability to PG_+_M (*P* = 2.7E−09). No SNPs were identified as outliers by the HEIDI test. The pattern of results was the same when other MR methods were applied, which, as expected, showed less significant results (see Supplementary Note 4, Supplementary Table 22, and Supplementary Fig. 15). The bidirectional MR statistical significance between MD and PG_+_M could be consistent with reverse causality or pleiotropy. To exclude potential known confounders we repeated analyses with GWAS summary statistics conditioned on GWAS summary statistics of on EA, BMI, and smoking-related traits from mtCOJO analysis. The results remain significant in both directions (Supplementary Table 23). We used the latent causal variable (LCV) method^69^, which is designed to better separate pleiotropy from causality. As expected, the genetic causality proportion is not significant for PG_+_M and MD because of the strong bidirectional significance (Supplementary Note 4 and Supplementary Table 24). As sensitivity analyses, we repeated the analyses after removing the depression cases from both cases and controls from the five GI disorder phenotypes (Supplementary Note 5 and Supplementary Tables 25–27). In summary, the statistically non-significant GSMR results in this study neither support a causal relationship from MD to IBD nor from IBD to MD. The results for PG_+_M do not support a unidirectional causal relationship given the significant bidirectional GSMR results. The MR results between MD and PUD, support a putative causal role of MD in PUD, but should be revisited when more SNP instruments are available.

### Out-of-sample polygenic score prediction

To further investigate the relationship between MD and PG_+_M, we used MD GWAS summary statistics (European ancestry, excluding the UKB cohort)^48^ to generate MD polygenic scores and used these to predict PG_+_M risk in the UKB. We found that participants in the UKB with a high polygenic score for MD had a higher risk for PG_+_M-related disorders. The top decile of individuals ranked on polygenic risk prediction for MD had an OR of 1.33 (95% CI: 1.29–1.38) for PG_+_M risk compared to the bottom decile (Fig. 6c and Supplementary Table 28). We also selected genome-wide significant SNPs associated with PG_+_M in the UKB to calculate polygenic scores and predict depression and psychiatric disorder risk in GERA cohort^36^, as shown in Fig. 6d respectively. Further, we selected genome-wide significant SNPs associated with PUD, IBS in the UKB to calculate polygenic scores and predict peptic ulcer and IBS risk in GERA cohort^36^. The top decile of individuals ranked on polygenic score for PUD had an OR of 1.80 (95% CI: 1.38–2.36) for PUD risk compared to the bottom decile and that for IBS had an OR 1.42 (95% CI: 1.21–1.67) for IBS risk compared to bottom decile (Supplementary Fig. 16).

## Discussion

This study describes an analysis of PUD and its relationship with other digestion disorders using a single study cohort. We used both the phenotypes and genotypes of up to 456,327 individuals to study the genetic contributions to PUD, GORD, IBS, and IBD and the connection between these disorders with major depression. Our results provide insights into the genetic basis of, and inter-relation between, these gastrointestinal disorders and also their relation to depression.

GWAS of PUD identified eight independent associated loci and 6 of 8 have potential links to *H. pylori* infection, highlighting the role of host genetic susceptibility (Fig. 2). Only two SNPs (rs2294008 and rs505922) have been previously reported as associated with peptic ulcers, both from a Japanese cohort study^18^ of duodenal ulcers. The authors provided evidence for their role in with duodenal ulcer development after *H. pylori* infection^18^. In our PUD-associated SNPs, rs2976388, located in *PSCA* gene, is in high LD (*r*^2^ = 0.94 in Europeans) with rs2294008, while rs687621, an intronic SNP located in the blood-group *ABO* gene, is in high LD (*r*^2^ = 0.98 in Europeans) with rs505922 (Fig. 2). These two loci are reported here in Europeans with the same direction of effect. From published gene expression data, we found that allele A of SNP rs2976388 is associated with increased *PSCA* expression (*b*_eQTL_ = 0.73, *P*_eQTL_ = 8.8E−41), and through SMR analysis, the increased expression of *PSCA* decreased risk for PUD (*b*_SMR_ = −0.12, *P*_SMR_ = 3.0E−11). Decreased *PSCA* expression has been reported following *H. pylori* infection^70^, indicating negative regulation of *PSCA* expression by *H. pylori* infection. Other data sets recorded for *H. pylori* infection status are needed to explore this proposed relationship. A previous study^71^ has shown that individuals with blood group O have a higher risk of peptic ulcers compared to those with other blood groups which offers a possible explanation that this observation may result from different susceptibilities and immunologic responses to *H. pylori* infection. The A allele of rs687621 is associated with PUD (OR = 1.08, Table 2), and this allele is in high LD (*r*^2^ = 0.86 in Europeans and *r*^2^ = 0.89 in Japanese) with the rs8176719 deletion allele which generates a premature stop codon that leads to the O blood group^72^. We note that rs505922 has similar LD (*r*^2^ = 0.87) with rs8176719 in Europeans, but is in slightly higher LD with rs8176719 in Japanese (*r*^2^ = 0.92). This finding deserves further investigation of the association between blood group and peptic ulcer. Of the six previously unreported PUD-associated SNPs, it is notable that rs681343 is statistically significant in both UKB discovery and GERA replication GWAS (a replication rate consistent with power, see Supplementary Table 5). This SNP is located in the *FUT2* gene which has been implicated in susceptibility to *H. pylori* infection in humans^73^ and mice gastric tissue^74^. The *FUT2* fucosyltransferase allows expression of blood group antigens on the gastrointestinal mucosa and in bodily secretions. European individuals who are homozygous (AA) at rs601338 (non-secretor phenotype) are unable to secrete blood group antigens into bodily fluids, or express them on mucosal surfaces given the allele AA encodes a stop codon that inactivates the *FUT2* enzyme^75^. Moreover, rs601338 is also associated with different infections (either resistance or susceptibility)^75^ and rs681343 associated with PUD in our analyses is in high LD with rs601338 in Europeans (*r*^2^ = 0.996), with the T allele of rs681343 paired with the A allele of rs601338.

Another two of the PUD-associated SNPs are in located in genes encoding mucin peptides, located on different chromosomes: rs147048677 (*P* = 9.0E−12) is a synonymous variant in the *MUC1* gene. A mouse model study^76^ has shown that Muc1 limits *H. pylori* colonization of gastric mucosa. rs78459074 (*P* = 2.6E−10) is an intronic variant in the *MUC6* gene. We note that, *MUC6*, *MUC2*, and *MUC5AC* are sequentially co-located on chromosome 11 (Supplementary Fig. 4). The expression of *MUC6* and *MUC5AC* have been linked to *H. pylori* infection^77,78^. For the other three PUD-associated SNPs, rs9581957 is 22 kb away from the *CDX2* gene (Supplementary Fig. 4). In nonneoplastic cell lines *H. pylori* infection led to prompt and robust expression of Cdx2^79^. rs10500661 (*P* = 4.1E−14) is located in ~7 kb upstream of *CCKBR* (cholecystokinin B receptor) and this gene encodes a G-protein coupled receptor for both gastrin and cholecystokinin, regulatory peptides of the brain and gastrointestinal tract^80^. This gene, as shown in the GTEx^60^ portal (https://gtexportal.org/home/gene/CCKBR), is highly expressed in the brain frontal cortex (Brodmann area (BA) 9) and stomach. Moreover, this gene is a therapeutic-effect target gene for a peptic ulcer drug proglumide (ATC code: A02BX06), which inhibits gastrointestinal motility and reduces gastric acid secretions. In addition to these findings, itriglumide, an antagonist for the *CCKBR*-encoded protein, has been investigated as a potential treatment for anxiety and panic disorders^81^. rs34074411 is located approximately 2 kb upstream of the *GAST* gene which encodes gastrin, a hormone whose main function is to stimulate secretion of hydrochloric acid by the gastric mucosa. It is unusual to be able to link 6 out of 8 GWAS associated loci for a trait to a putative mechanism, but here the results highlight the role of host genetic variability to bacterial infection. Given the high genetic correlation between PUD and GORD (0.65, SE = 0.05), we also conducted mtCOJO analyses for PUD conditioning on GORD GWAS summary statistics. All the SNPs mentioned above still showed statistical significance (Supplementary Table 29), suggesting that most of those PUD-associated SNPs are specific to PUD, and may provide important disorder specific profiles for use in clinical trials.

We identified 6, 19, and 2 independent significant associations for GORD, PG_+_M, and IBS, respectively. Some genes around these loci have biological support for their mechanistic involvement and are worthy of note. For PG_+_M, two of the significantly associated SNPs, 19:18793695 and rs13097265, have been previously linked to Barrett’s esophagus and esophageal adenocarcinoma^82^ (Supplementary Table 4). rs13097265 is located ~60 kb from the *FOXP1* gene, and recently a mouse study^83^ has shown that Foxp1 protein is expressed in all layers of the murine GI tract (including the myenteric plexus, which is part of the enteric nervous system and regulates gut peristalsis and transit). Altered motility and achalasia has been observed in *Foxp1*^+/−^ mice. Heartburn and regurgitation, the main GORD symptoms, commonly occur during the early stages of achalasia and are consequently poor indicators of esophageal motility disorder. It remains controversial whether GORD and achalasia coexist or whether one disease transforms into the other^83^. Interestingly, rs10891491 associated with GORD (*P* = 4.1E−08) and rs7947502 associated with IBS (*P* = 2.5E−08) are in LD (*r*^2^: 0.15) (Table 3). rs10891491 and rs7947502 are intronic variants of *NCAM1*, suggesting this genic region may show pleiotropic effect for these two disorders. *NCAM1* encoded protein is involved in development of the nervous system, including the enteric nervous system^84^. These GWAS results deserve further investigation to understand the role of genetic variants in the etiology of PUD, GORD, and IBS, especially since many of the genes putatively implicated by SNP associations have biological mechanism support for these disorders.

We provide direct genetic evidence for the difference between IBD and the other digestion phenotypes; although not unexpected, our analyses quantify these differences as illustrated by high genetic correlations among PUD, GORD, and IBS, which all show low genetic correlations with IBD (Fig. 4b). Both PUD and GORD are acid-related diseases; their high genetic correlation (*r*_g_ = 0.65, s.e. = 0.052) motivated the combination of GORD and PUD with medication-taking cases. Additional evidence for genetic differences between IBD and the other digestion phenotypes was provided by the partitioned SNP-based heritability analyses, which showed enrichment of PG_+_M-associated SNPs in genes expressed in the brain regions (hippocampus, BA9 and BA24) while those for IBD are enriched in blood and immune related tissues (Supplementary Table 14). SNP-based heritability for PG_+_M is still enriched in BA9 region when we used fine-scaled GTEx brain gene expression data (Supplementary Table 15), and also after controlling for the effect of EA, BMI, and smoking (Supplementary Tables 13–15). Gene set enrichment analysis showed PG_+_M-associated SNPs are enriched in neuron-related gene sets. We note that a limitation of our brain enrichment analysis is our conclusions are limited by the availability of tissue specific gene expression data. The GTEx database does not report gene expression data for multiple cortical regions, so specificity to BA9 or frontal cortex (over and above other cortical regions) is not established. Given the non-availability of gene expression data from other human tissues, such as sympathetic, parasympathetic (vagus nerve)^26^, and enteric nervous system^25,27^, we cannot conduct key hypothesis-based enrichment analyses. However, despite these limitations, our findings indicate that a genetic contribution to PG_+_M may highlight the potential link between the nervous system and esophagus, stomach and duodenum^28,85^, although there is likely not just one causal tissue or cell type^59^. Historically, vagotomy was used commonly to manage peptic ulcer diseases, as vagal stimulation promotes acid secretion^86^ (now successfully treated by H2 receptor agonists), indicating the clinical importance of the link between the nervous system and gastrointestinal tract.

We observed significant increased risk of the four digestion disorders with depression (under multiple definitions of clinical and self-report) (Fig. 6a). The association between mental health and GORD has been addressed through observational studies^30,87^. For example^87^, a bidirectional association between GORD and depression was reported with risk factor roles for depression on GORD and for GORD on depression. Here, we conducted MR to investigate if this statistical approach provides evidence of a causal relationship between major depression and GI disorder phenotypes. Since, the number of genome-wide significant SNP instruments for GORD and PUD is too few to draw strong conclusions, we focus on the combined PG_+_M phenotype that identified a higher number genome-wide significant SNPs for use as the MR instruments. We found an OR of 1.27 (*P* = 2.5E−15) for PG_+_M per SD in liability to MD, which has direction and effect size estimates consistent with those previously reported between MD and drugs for GORD and PUD (OR: 1.23, *P* = 4.0E−6)^88^. However, the reverse MR analysis (the effect of PG_+_M on MD) is also significant (OR: 1.26, *P* = 2.7E−9). The bidirectional statistically significant results from GSMR usually include two interpretations, one is that there is the bidirectional causality and the other one is horizontal pleiotropy, including an indirect relationship through an intermediate endophenotype or confounder^69,89^. Hence, our conclusions must include these interpretations, although we note that the MR Egger intercept test suggests no horizontal pleiotropy (Supplementary Table 22), and the results were similar after mtCOJO conditional analyses, which take into account correlated traits such as educational attainment, BMI, and smoking. In terms of bidirectional causality, there are several possible explanations. Patients with psychological comorbidity often perceive low intensity esophageal stimulation as being painful due to hypervigilance to these intra-esophageal events^85^. Psychological factors can decrease the pressure of the lower esophageal sphincter and change esophageal motility^90^. The reflux symptom itself could result in depression through potentially disabling effects on occupational or social function, or if patients are constantly feeling upset about their condition^90^. Use of medications could conceivably mediate bidirectional associations between depression and PG_+_M. Tricyclic antidepressants can lead to a decrease in lower esophageal sphincter pressure, and thus and increase in the number of reflux episodes (anticholinergic effect)^91^. Recently, a study shows that use of proton-pump inhibitors (PPI) for acid-related disorders are associated with the subsequent risk of major depression disorder^92^. Further studies are needed to clarify this association. We note a recent population based study of 83.9 million person-years^93^ found that a diagnosis of mood disorder was associated with increased risk (hazard ratio 1.57) of subsequent peptic ulcer disease/gastritis. While a causal relationship cannot be confirmed between major depression and digestion related disorders (or vice versa), consideration of clinical implications of a possible relationship is justified. When treating patients with MD, awareness of the digestion symptoms for PUD/GORD could help to decide if further interventions are needed. Also, these results may provide clues for screening psychological factors in PUD/GORD patients. A previous study^94^ shows that GORD patients who are also comorbid with psychological distress have more severe symptoms at baseline and more residual symptoms after PPI treatment. In those patients, treatment for the underlying psychological distress might improve the PPI response. A notable negative result was the absence of evidence for any causal relationship between IBD and major depression (an OR of 1.08 (*P* = 0.4) when major depression was the exposure trait and IBD as outcome and 1.01 (*P* = 0.11) for the reverse direction). A causal relationship might be expected from observational studies and mechanistic theory linking inflammation with depression^95^, suggesting that observed phenotypic associations may be driven by residual confounding, and having potential implications for future research into the role of inflammation in the pathogenesis of depression.

Despite these interesting findings, our study has several limitations. First, the phenotypes of PUD, GORD, and IBS were combinations of self-reported illness, primary care, hospital admission record, and/or medication use. There is a potential influence regarding accuracy of self-report and misdiagnosis, however, the correlation in effect sizes from case-ness defined by hospital admission, primary care, or self-report data support the use of self-report data, Supplementary Fig. 10e). Given the existent co-reporting of some diagnoses, we conducted sensitivity analyses in which individuals recorded with more than one diagnosis were excluded, but these analyses did not impact our conclusions (Supplementary Tables 6–7, and 11). Formal diagnosis of PUD requires an endoscopy or a upper gastrointestinal barium test, moreover non-ulcer dyspepsia (NUD), not considered here, can be misdiagnosed as PUD. This could influence the downstream MR analyses with depression. Future large datasets of formal-diagnosed PUD are needed to further explore the MR analyses with major depression. Importantly, we note that the GWAS results from IBD derived from the UKB were highly consistent with results from published GWAS, despite a higher prevalence in the UKB than expected in a random population sample. We specifically focus on the broad definition of our phenotypes, and hence heterogeneity in the phenotypes has not been explored. Second, our study is conducted in the UKB cohort, which although a large population study, has recognized volunteer bias^96^. Third, we do not have replication data sets for GORD and PG_+_M genome-wide significant SNPs. Fourth, we do not have the *H. pylori* infection status and microbiome data for all the UKB participants, thus additional analyses on these factors cannot be further explored.

In summary, we identified 35 independent SNPs associated with different digestion disorders, of which 19 SNPs are previously unreported, including eight SNPs at or near *MUC1*, *MUC6, FUT2*, *PSCA*, *ABO*, *CDX2, GAST*, and *CCKBR* genes associated with peptic ulcer disease. Previously established roles of these genes in *H. pylori* infection, response to counteract infection-related damage and gastric secretion support their involvement. Post-GWAS analyses highlighted the link between the nervous system and the gastrointestinal tract. In addition, MR analyses imply a bi-directional relationship (the risk of GORD, PUD, and taking corresponding medications in liability to major depression and vice versa), which may reflect bidirectional causality, or pleiotropic effects between them. Taken together, our findings demonstrate the role of genetic variants in the etiology of common digestion disorders and the link between depression and PUD/GORD.

## Methods

### UK Biobank genotyping and quality control

The United Kingdom Biobank (UKB) cohort is a population-based volunteer longitudinal cohort of ~500,000 individuals recruited at 22 centers across the United Kingdom^97^. Genotype data from these individuals were imputed using the Haplotype Reference Consortium (HRC) and UK10K as the reference sample. A European ancestry subset (456,327 individuals, including 348,441 unrelated individuals) was identified by projecting the UKB participants onto the 1000 Genome Project principal components coordinates^98^. Genotype probabilities were converted to hard-call genotypes using PLINK2^99^ (hard-call 0.1) and single nucleotide polymorphisms (SNPs) with minor allele count < 5, Hardy–Weinberg equilibrium test *P* value <1.0E−5, missing genotype rate > 0.05, or imputation accuracy (Info) score < 0.3 were excluded.

### Phenotype definition

The UKB phenotypes used in analyses were derived from two categories: one is disease-diagnoses phenotypes from either a death register, self-reported, hospital admission, or primary care record for the corresponding disease and the other is treatment phenotypes based on the operation and medication-taking code. Supplementary Table 1 lists the corresponding case definition and number of cases for each phenotype. PUD disease-diagnoses cases are a combination of gastric ulcer cases (UKB data field: 131591), duodenal ulcer cases (UKB data field: 131593), other site peptic ulcer cases (UKB data field: 131595) and gastro-jejunal ulcer cases (UKB data field: 131597). The remaining individuals were PUD controls. There were 16,666 cases and 439,661 controls for PUD. For the GORD disease-diagnoses phenotype (UKB data field: 131585), there are 54,854 cases and 401,473 controls. Inflammatory bowel disease (IBD)^100^ disease-diagnoses phenotype cases are a combination of Crohn’s diseases (UKB data field: 131627), ulcerative colitis (UKB data field: 131629) diagnoses, giving a total of 7045 cases and 449,282 controls. There were 29,524 cases and 426,803 controls for irritable bowel syndrome (IBS, UKB data field: 131639). For the comorbidity, full-sibling risk and heritability estimation analyses, we used this IBS phenotype definition. For the IBS GWAS analyses, we first removed 1006 participants who also had IBD diagnoses from the original IBS cases, the remaining 28,518 participants were assigned to case status for the IBS disease-diagnoses phenotype while the other 426,803 participants were coded as IBS controls. In clinical practice, medications for PUD also have a therapeutic effect on GORD. Thus, we combined 55,865 individuals taking medications that are mainly considered medications for GORD/PUD (UKB data field: 6154 and 20003), 1162 participants with endorsement for anti-reflux operations (UKB data field: 41272) and diseases-diagnoses cases for GORD and PUD, leaving a total of 90,175 cases (41,945 male cases) and 366,152 controls (phenotype abbreviation: PG_+_M—for GORD, PUD, and corresponding medications and treatment). Since the medications for IBD and IBS from UKB can also be used to treat other diseases or relieve symptoms (i.e., medications are not specific), we did not incorporate the medication data for the IBD and IBS phenotypes. The Supplementary Data 1 of Wu et al.^88^ provides UKB medication classification based on Anatomical Therapeutic Chemical (ATC) Classification System^101^, and we extracted medications for GORD/PUD (the first two ATC level: A02, Supplementary Table 2). For the validation of UKB PUD and IBS genome-wide significant SNPs, we used Genetic Epidemiology Research on Aging (GERA)^36^ PUD and IBS summary statistics from Zhu et al.^37^. The definition of PUD and IBS phenotypes from the GERA cohort is described in The Supplementary Table 4 of Zhu et al.^37^. Briefly, the total 61,847 individuals were divided into two groups: 1004 peptic ulcer cases according to ICD9 code (531–534) and 60,843 controls, respectively. There are 3359 cases and 58,488 controls for IBS phenotypes using the ICD9 code (564). Sensitivity phenotypes for each of PUD, GORD, IBS, and IBD were regenerated by excluding individuals with more than one GI diagnosis among these four disorders from the corresponding original phenotype cases (Supplementary Note 3). To understand the genetic similarity and difference among the self-report, primary care and hospital admission data for each of the PUD, GORD, and IBS, we further divided the cases of each of the PUD, GORD, and IBS into three groups according to the UKB coding (3rd column of Supplementary Tables 1 and 30: primary care only, 40: hospital admission data only, and 50: self-report only) and generated subgroup phenotypes using those subgroup cases, together with the controls from each of the original phenotypes.

### Comorbidity analyses

Comorbidity analyses, including comorbidity amongst PUD, GORD, IBS, and IBD and comorbidity between each with depression phenotypes in the UKB, were conducted in 348,441 unrelated individuals. Among the four digestive diseases, for each two of the PUD, GORD, IBS, and IBD cases (six pairs in total), we first checked whether the number of overlapped individuals between case groups is statistically significantly larger than the overlap expected by chance. For each of the PUD, GORD, IBS, and IBD disease (defined as the index disease), we conducted competitive comorbidity analyses to test among the other three diseases which disease is more prone to be comorbid with the index disease. Briefly, we calculated the proportion of the index disease cases in the other three diseases respectively and compared them in pairs by using two-proportions *Z*-test. One prerequisite for the two-proportion *Z*-test is that the two samples are independent of each other. Given this, we removed the overlapped cases among these three diseases when calculating the proportion of the index disease cases. For comorbidity between each of the PUD, GORD, IBS, and IBD with depression phenotypes, we first derived eight depression phenotypes based on different data field (UKB data field: 20002, 20216, 2090, 20440, 20442, 2100, 41202, and 41204) and mental health online follow-up (data category: 138) from the UKB. The details for depression phenotype definitions were described Cai et al.^68^. Briefly, depression phenotypes were defined according to help seeking behavior and symptoms, including seen general practice (GP) for nerves, anxiety, tension or depression (abbreviation: GPpsy), seen psychiatrist for nerves, anxiety, tension, or depression (abbreviation: Psypsy), probable recurrent major depression or single probable major depression episode (abbreviation: DepAll), self-reported depression (abbreviation: SelfRepDep), ICD10 defined depression (abbreviation: ICD10Dep), DSM V clinical guideline defined major depression (abbreviation: LifetimeMDD), major depression recurrence (abbreviation: MDDRecur) and seen GP for depression but no cardinal symptoms (abbreviation: GPNoDep) phenotypes. We then checked the overlap individuals from each of the GORD, PUD, IBS, and IBD with the eight depression phenotypes. For each of 32 digestion-depression phenotype pairs, we tested whether the number of individuals who are both digestion phenotype case and depression phenotype case is statistically significantly different from the expected number. Odds ratio, 95% confidence interval (CI) and corresponding *P* value for each digestion-depression phenotype pair were calculated using fmsb R package (https://cran.r-project.org/web/packages/fmsb/fmsb.pdf). Bonferroni correction was used to account for multiple testing.

### Full-sibling risk and estimation of heritability

To demonstrate the genetic component for the UKB case–control phenotypes of PUD, GORD, IBS, and IBD, we estimated the increased risk of the disorders in full-siblings of those affected (we did not incorporate other relative pairs data given the limited sample size for disease cases), compared to the risk in all UKB individuals (disease risk). As described by Bycroft et al.^97^, 22,665 full-sibling pairs were inferred from the kinship coefficients estimated using KING^102^. Risk ratio, 95% CI and corresponding *P* value were calculated using fmsb R package. After obtaining the full-sibling relative risks, we used liability distribution theory^103–105^ to estimate the heritability of each trait, under the assumption that the increased risk only reflects shared genetic factors.

### Genome-wide association study (GWAS) analyses

We performed case–control GWAS analyses using BOLT-LMM^106^ with sex, age and 20 ancestry principal components (PCs) fitted as covariates. 543,919 SNPs generated by linkage disequilibrium (LD) pruning (*r*^2^ < 0.9) from HapMap3 SNPs were used to control for population structure and polygenic effects, including genetic relatedness between individuals. The effect size (*β*) from BOLT-LMM on the observed 0–1 scale were transformed to odds ratio (OR) using the following equation^107^: ${\mathrm{OR}} = \frac{{\left( {k + \beta \left( {1 - p} \right)} \right. \times \left( {1 - k + \beta p} \right)}}{{\left( {k - \beta p} \right) \times \left( {1 - k - \beta \left( {1 - p} \right)} \right)}}$, where *k* is the proportion of sample that are cases, and *P* is the allele frequency in the full UKB European cohort. The standard error (s.e.) for OR were then calculated based on the OR and *P* value from the initial GWAS using the formula ${\mathrm{s}}.{\mathrm{e}}. = \left| {\frac{{\ln ({\mathrm{OR}})}}{{{\mathrm{{\Phi}}}^{ - 1}\left( {\frac{{\mathrm{P}}}{2}} \right)}}} \right|$. A total of 8,546,065 SNPs with minor allele frequency (MAF) > 0.01 were analysed. Independent trait-associated SNPs were generated using GCTA-COJO^34,35^ analyses. The genotype data (8,546,065 SNPs with MAF > 0.01) of 20,000 random sampled unrelated European individuals were used to provide a LD reference. Due to the complexity of major histocompatibility complex (MHC) region (25–34 Mb), only the most significant SNP across that region was reported. Regional visualization plots were produced using LocusZoom^108^. The genomic inflation factor (λ_GC_) was also reported for each phenotype. We used PUD and IBS GWAS summary statistics^37^ from the GERA^36^ cohort for validation look-up of the UKB PUD and IBS genome-wide significant SNPs, respectively. We applied RPower package^109^ to calculate power for UKB PUD and IBS genome-wide significant SNPs give GERA sample size for each trait. We also conducted pleiotropy (SNP associated with multiple traits) analysis. Briefly, we downloaded published GWAS associations from the GWAS Catalog^39^ on April 6th 2020. For each of PUD, GORD, PG_+_M, and IBS associated SNPs in our study (index SNP), we first selected SNPs from the GWAS Catalog within a ±1000 kb window size of the index SNP. We then selected the GWAS Catalog SNPs significantly associated (*P* < 5.0E−8) with either mental health-related traits or digestive diseases. We reported a pleiotropic association if selected GWAS Catalog SNPs are in LD (*r*^2^ > 0.1) with the index SNP. Similarly, for IBD-associated SNPs in our study, we checked whether significant association (*P* < 5.0E−8) have been reported for inflammatory bowel diseases (including the subtypes) using the downloaded GWAS Catalog data. GWASs for sensitivity phenotypes of each of PUD, GORD, IBS, and IBD were conducted. We also conducted GWASs for the three subgroup phenotypes of each of PUD, GORD, and IBS for further post-GWAS analyses, following the same procedures mentioned above. Given the sex specific effect of rs10512344 on IBS, we further conducted sex-specific GWAS on IBS. To explore the specificity of PUD-associated SNPs, we conducted a multitrait conditional and joint (mtCOJO)^37^ analysis to condition the PUD GWAS results on GORD GWAS summary statistics and check whether the original PUD-associated SNPs remain significant. We used the GENE2FUNC of FUMA pipeline^38^, together with GTEx 8th version gene expression data^60^, to investigate whether the genes surrounding PUD-associated SNPs are overexpressed in any specific tissue.

### SNP-based heritability and genetic correlations of the five digestion phenotypes

Linkage disequilibrium score regression (LDSC)^40^ was used to estimate SNP-based heritability ($h_{{\rm{SNP}}}^2$) from the GWAS summary statistics. The $h_{{\rm{SNP}}}^2$ estimated on the observed scale were transformed to the liability scale taking the sample lifetime risk (proportion of sample that are cases) as the disease lifetime risk estimates. The summary statistics for each phenotype were filtered using the LDSC default file, w_hm3.snplist, with the default LD scores computed using 1000 Genomes European data (eur_w_ld_chr) as a reference. Genetic correlations (*r*_g_) for each two of the five UKB digestion phenotypes or between each of the five phenotypes and the 258 GWAS traits in LD Hub^41^ were calculated using bivariate LDSC^110^. Since we had a particular interest in the relationship between the five digestion phenotypes and disorders of nervous system, we also used more recent GWAS summary statistics than those included in LDHub for seven psychiatric traits (attention deficit/hyperactivity disorder (ADHD)^42^, schizophrenia (SCZ)^43^, anxiety disorder^44^, posttraumatic stress disorder (PTSD)^45^, bipolar disorder (BIP)^46^ and autism spectrum disorder (ASD)^47^, major depression (MD)^48^) and two neurological traits (Alzheimer’s disease based on family history^49^ and Parkinson’s disease^50^). As sensitivity analyses, we repeated analyses using sensitivity phenotypes (excluding any individual recorded to have more than one disorder for each of PUD, GORD, IBS, and IBD). Although removal of these individuals could make estimates of SNP-based heritability difficult to interpret, genetic correlations are more robust to such ascertainment^89^. We also used three subgroup phenotypes for each of PUD, GORD, and IBS, as mentioned above, to calculate SNP-based heritibilities and within-diseases genetic correlations. Note, as shown by theory and simulation, estimates of genetic correlations are unbiased even in the context of sample overlap^110^. In the absence of sample overlap, the genetic covariance intercept term in the bivariate LDSC regression equation is expected to be zero. If sample overlap is present then the intercept term is expected to be a function of the phenotypic correlation, and the proportion of the samples that overlap between the two data sets.

### Linking GWAS findings to gene expression

Following the estimation of $h_{{\rm{SNP}}}^2$, we partitioned the SNP-based heritability by genomic features^111^. Briefly, in this method^111^, genetic variants are assigned into 53 functional categories using 24 publicly available annotation data sets, such as UCSC coding, UTRs, promoter and intronic regions^112^, conserved regions^113^ and functional genomic annotations constructed using ENCODE^114^ and Roadmap Epigenomics Consortium^66^ data. The method evaluates the contribution of each functional category to the overall $h_{{\rm{SNP}}}^2$ of a trait. A category is enriched for $h_{{\rm{SNP}}}^2$ if the variants with high LD to that category have elevated *χ*^2^ statistics, compared to the expectation given the number of SNPs in the category. In another analysis, genetic variants were annotated to histone marks (H3K4me1, H3K4me3, H3K9ac, and H3K27ac) by cell type specific classes and these annotations were allocated to ten groups: adrenal and pancreas, central nervous system (CNS), cardiovascular, connective and bone, gastrointestinal, immune and hematopoietic, kidney, liver, skeletal muscle, and other. We tested the enrichment of $h_{{\rm{SNP}}}^2$ in tissues relevant to the five digestion phenotypes: the adrenal and pancreas, gastrointestinal, immune and hematopoietic, and liver cell types. We also considered the CNS given the high *r*_g_ between four of the six digestion phenotypes and depressive symptoms. We also used LDSC specific expressed genes (SEG)^59^ analysis to test the enrichment of $h_{{\rm{SNP}}}^2$ through gene expression derived cell-type specific annotations. First, given the strong contribution to PG_+_M $h_{{\rm{SNP}}}^2$ from the CNS and to IBD $h_{{\rm{SNP}}}^2$ from the immune cell group, LDSC-SEG^59^ was applied to test the enrichment of $h_{{\rm{SNP}}}^2$ in 205 different tissues (53 from GTEx and 152 from Franke lab). Second, given the observation that GORD, PG_+_M, and IBS $h_{{\rm{SNP}}}^2$ were enriched in the CNS, we also applied LDSC SEG to test the enrichment of $h_{{\rm{SNP}}}^2$ for GORD, PG_+_M, and IBS in 13 brain regions using the multiple brain regions available in the GTEx study (https://gtexportal.org/home) data^60^ to identify specific brain regions implicated by the GWAS results for the three phenotypes. Bonferroni correction was used to account for multiple testing. As sensitivity analyses, we conducted a multitrait conditional and joint (mtCOJO)^37^ analysis to condition the PG_+_M GWAS results on educational attainment (EA)^62^, body mass index (BMI)^54^, and tobacco-use^63^ GWAS summary statistics and further conducted LDSC cell type group and LDSC-SEG enrichment analyses using gene expression data from the tissues which are statistically significantly enriched above. We checked eQTL (expression quantitative trait loci, i.e., SNPs significantly (*P* < 5 × 10^−8^) associated with gene expression) status for each genome-wide significant SNP using GTEx^60^ results for gastrointestinal tissue and brain tissues. Summary-data-based Mendelian randomization (SMR)^64^ was used to provide evidence for likely causal relationship between the trait-associated SNPs and gene expression. We used eQTLGen^115^ whole blood eQTL data since this is the largest eQTL data set and many eQTLs are shared across tissues^116^. To capture more tissue specific eQTL, we used GTEx^60^ eQTL data from six tissues: esophagus-gastroesophageal junction, esophagus mucosa, esophagus muscularis, stomach, small intestine terminal ileum, colon sigmoid, and colon transverse tissue. We also used GTEx eQTL data from hippocampus, brain anterior cingulate (Brodmann area 24, BA24), frontal cortex (Brodmann area 9, BA9) tissue for PG_+_M given $h_{{\rm{SNP}}}^2$ enrichment results. The Bonferroni corrected significance threshold was 0.05/131,295, where 131,295 is the number of total genes tested in SMR analyses. Because of its complexity, we do not report results of the MHC region (25–34 Mb)^48^. The statistical framework of SMR^64^ can be applied to mQTL (genome-wide significant SNP-methylation association) data to identify putative methylation-trait association. Hence, we repeated SMR analyses for PUD using blood mQTL data from McRae et al.^65^.

### Gene-based and gene-set enrichment analyses

MAGMA (v1.06)^67^ (Multi-marker Analysis of GenoMic Annotation) was used to test for gene-based association based on the SNP association results of the five digestion phenotypes. Gene length boundaries were defined as 35 kilobase (kb) upstream and 10 kb downstream from start and stop site, respectively, to include regulatory elements. The NCBI 37.3 build was used to assign the genetic variants to each gene. SNPs with MAF > 0.01 from 20,000 randomly sampled and unrelated UKB European-ancestry individuals were used to provide a LD reference. A total of 18,402 genes were assessed for an association with each of the six digestion phenotypes with Bonferroni correction used to determine significance (*α* = 0.05/18402, *P* < 2.7E−6). We used the results obtained from gene-based analysis, together with gene ontology sets (c5.cc) from MSigDB^117,118^ to conduct gene-set enrichment analyses. Competitive test *P* value for each gene set, as implemented in MAGMA, were computed taking gene size, density, minor allele count and gene–gene correlation into consideration^67^. False discovery rate (FDR)-adjusted *P* values for biological pathways for each of the five digestion phenotypes were generated using Benjamini and Hochberg’s method^119^ to account for multiple testing.

### Mendelian randomization

We applied the generalized summary-data-based Mendelian randomization (GSMR)^37^ method to explore the potentially causal effect of MD as an exposure on the five UKB digestion phenotypes as outcome traits (defined as forward direction). GSMR uses summary-level data to test for causal associations between a putative risk factor (exposure) and an outcome trait. Independent genome-wide significant SNPs from the MD GWAS (excluding UKB cohort)^48^ were used as the MR exposure instrument variables. The HEIDI outlier test^37^ was used to remove outlier pleiotropic genetic instruments associated with both exposure phenotype and outcome phenotype from the analysis. We also conducted reverse causation analysis (i.e., testing the opposite hypothesis that the five digestion phenotypes cause MD). However, GSMR guidelines advise the use of at least ten independent lead SNPs as genetic instruments to achieve robust results. In order to test the effect of PUD (eight SNPs with *P* < 5.0E−8), GORD (six SNPs), and IBS (two SNPs) on MD, we relaxed the significance threshold to allow for at least ten SNPs for each of the three phenotypes. For each trait as exposure, we only keep the most significant SNP within MHC region for MR analysis (if applicable). We used 0.01 as a clumping threshold *r*^2^ in GSMR analyses. The other parameters were set to software defaults. For comparison, we also conducted IVW-MR^120^, MR-Egger^121^, weighted median-MR^122^, and MR-PRESSO^123^ analyses following the STROBE-MR guideline^124^ for the effect between PG_+_M and MD. We also used LCV^69^ approach for PG_+_M and MD given the fact that this approach could control pleiotropy well. To explore whether the bidirectionally statistically significant GSMR results between PG_+_M and MD is mediated by EA^62^, BMI^55^, and tobacco-use^63^ effect, we further used mtCOJO results to conduct bidirectional GSMR analyses, i.e., taking MD conditional GWAS results (the MD original GWAS results conditioned on EA^62^, BMI^55^, and tobacco-use^63^ GWAS summary statistics separately or together) as exposure and PG_+_M non-conditional GWAS results as outcome and vice versa. As a sensitivity analysis, we further removed the depression cases (the combined cases from eight depression phenotypes in UKB as aforementioned) from the five GI disorders phenotypes and conducted GWASs for the five depression removed sensitivity phenotypes (defined as GI-DepComRMV phenotypes). We further repeated bivariate LDSC analyses for genetic correlation and GSMR analyses for MR between major depression and GI-DepComRMV phenotypes. In addition to this, we removed the depression cases of each of eight depression phenotypes in UKB from PUD phenotypes and generated eight PUD sensitivity phenotypes. We further repeated GSMR analyses for MR between major depression and eight PUD sensitivity phenotypes.

### Out-of-sample polygenic score prediction

We used MD GWAS summary statistics^48^ (European ancestry, excluding UKB cohort) as discovery data to predict PG_+_M risk (risk for PUD, GORD, and likelihood for taking PUD, GORD medications or treatments). The MD data SNPs were matched with the PG_+_M SNPs, then LD pruned and “clumped”, discarding variants within 1000 kb of, and in *r*^2^ ≥ 0.1 with, another (more significant) marker using SNPs with MAF > 0.01 from 20,000 random sampled unrelated UKB European-ancestry individuals as the LD reference. Polygenic score of PG_+_M sample individuals were generated for a range of MD GWAS summary statistics data association *P* value thresholds (5.0E−8, 1.0E−5, 1.0E−4, 1.0E−3, 1.0E−2, 0.05, 0.1, 0.5). For each discovery-target pair, three outcome variables were calculated. (1) The *P* value of case-control polygenic score difference from logistic regression. (2) Area under the receiver operator characteristic curve using R package pROC^125^, which can be interpreted as the probability of ranking a randomly chosen case higher than a randomly chosen control. (3) Odds ratio and 95% confidence interval for the 2nd–10th polygenic score deciles group compared with 1st decile. The polygenic score decile presented in Fig. 6c is based on association *P* value thresholds 1.0E−2. Given the availability of depression and psychiatric disorders (not GORD) phenotypes in GERA cohort, we used UKB PG_+_M GWAS summary statistics to calculate polygenic score for GERA individuals based on *P* value threshold 0.05 and conducted out-of-sample polygenic score prediction for depression and psychiatric disorders risk in GERA individuals, respectively. We further conducted out-of-sample prediction for PUD and IBS risk in GERA cohort following the same steps but the *P* value threshold for polygenic score calculation are 5E−8 and 0.1, respectively.

### Reporting summary

Further information on research design is available in the Nature Research Reporting Summary linked to this article.

## Supplementary information

Supplementary Information

Description of Additional Supplementary Files

Supplementary Data 1

Supplementary Data 2

Supplementary Data 3

Supplementary Data 4

Supplementary Data 5

Reporting Summary

## Data Availability

Association summary statistics from analyses presented here are available at https://cnsgenomics.com/content/data. The data that support the findings of this study are available from UK Biobank (http://www.ukbiobank.ac.uk/about-biobank-uk/). Restrictions apply to the availability of these data, which were used under license for the current study (ID: 12505). Data are available for bonafide researchers upon application to the UK Biobank. We also used peptic ulcer disease and irritable bowel syndrome GWAS summary statistics (https://cnsgenomics.com/content/data) from the Resource for the Genetic Epidemiology Research on Adult Health and Aging (GERA: dbGaP phs000674.v2.p2, https://www.ncbi.nlm.nih.gov/projects/gap/cgi-bin/study.cgi?study_id=phs000674.v2.p2) study. We used GWAS summary statistics for major depression that include data from 23andMe. These data can be obtained by qualified researchers under an agreement with 23andMe that protects the privacy of the 23andMe participant 23andMe. Researchers can perform meta-analysis of 23andMe summary statistics and the other five-cohort results file, as described in Wray et al., to get major depression GWAS summary statistics (excluding UK Biobank cohort). The data for generating the figures are provided in the Supplementary Information and Supplementary Data.

## Code availability

All the code for the analyses in this study are available upon the request.

## References

[1] Peery AF (2019). Burden and cost of gastrointestinal, liver, and pancreatic diseases in the United States: update 2018. Gastroenterology.

[2] Williams JG (2007). Gastroenterology services in the UK. The burden of disease, and the organisation and delivery of services for gastrointestinal and liver disorders: a review of the evidence. Gut.

[3] Whitehead WE, Palsson O, Jones KR (2002). Systematic review of the comorbidity of irritable bowel syndrome with other disorders: What are the causes and implications?. Gastroenterology.

[4] Vakil N, van Zanten SV, Kahrilas P, Dent J, Jones R (2006). The Montreal definition and classification of gastroesophageal reflux disease: a global evidence-based consensus. Am. J. Gastroenterol..

[5] Lanas A, Chan FKL (2017). Peptic ulcer disease. Lancet.

[6] Charpignon C (2013). Peptic ulcer disease: one in five is related to neither Helicobacter pylori nor aspirin/NSAID intake. Aliment Pharm. Ther..

[7] Böhmer AC, Schumacher J (2017). Insights into the genetics of gastroesophageal reflux disease (GERD) and GERD-related disorders. Neurogastroenterol. Motil..

[8] El-Serag HB, Sweet S, Winchester CC, Dent J (2014). Update on the epidemiology of gastro-oesophageal reflux disease: a systematic review. Gut.

[9] Canavan C, West J, Card T (2014). The epidemiology of irritable bowel syndrome. Clin. Epidemiol..

[10] Camilleri M (2012). Peripheral mechanisms in irritable bowel syndrome. N. Engl. J. Med..

[11] Ananthakrishnan AN (2015). Epidemiology and risk factors for IBD. Nat. Rev. Gastroenterol. Hepatol..

[12] Ng SC (2017). Worldwide incidence and prevalence of inflammatory bowel disease in the 21st century: a systematic review of population-based studies. Lancet.

[13] Malaty HM, Graham DY, Isaksson I, Engstrand L, Pedersen NL (2000). Are genetic influences on peptic ulcer dependent or independent of genetic influences for helicobacter pylori infection?. Arch. Intern. Med..

[14] Mohammed I, Cherkas LF, Riley SA, Spector TD, Trudgill NJ (2003). Genetic influences in gastro-oesophageal reflux disease: a twin study. Gut.

[15] Saito YA (2011). The role of genetics in IBS. Gastroenterol. Clin. N. Am..

[16] Chen G-B (2014). Estimation and partitioning of (co)heritability of inflammatory bowel disease from GWAS and immunochip data. Hum. Mol. Genet..

[17] Verstockt B, Smith KGC, Lee JC (2018). Genome-wide association studies in Crohn’s disease: past, present and future. Clin. Transl. Immunol..

[18] Tanikawa C (2012). A genome-wide association study identifies two susceptibility loci for duodenal ulcer in the Japanese population. Nat. Genet..

[19] Bonfiglio, F. et al. A meta-analysis of reflux genome-wide association studies in 6750 Northern Europeans from the general population. *Neurogastroenterol. Motil.* **29**, e12923 (2017). 10.1111/nmo.12923 27485664

[20] An J (2019). Gastroesophageal reflux GWAS identifies risk loci that also associate with subsequent severe esophageal diseases. Nat. Commun..

[21] Ek WE (2015). Exploring the genetics of irritable bowel syndrome: a GWA study in the general population and replication in multinational case-control cohorts. Gut.

[22] Holliday EG (2014). Genome-wide association study identifies two novel genomic regions in irritable bowel syndrome. Am. J. Gastroenterol..

[23] Bonfiglio F (2018). Female-specific association between variants on chromosome 9 and self-reported diagnosis of irritable bowel syndrome. Gastroenterology.

[24] Vich Vila A (2018). Gut microbiota composition and functional changes in inflammatory bowel disease and irritable bowel syndrome. Sci. Transl. Med..

[25] Mayer EA (2011). Gut feelings: the emerging biology of gut–brain communication. Nat. Rev. Neurosci..

[26] Breit, S., Kupferberg, A., Rogler, G. & Hasler, G. Vagus nerve as modulator of the brain–gut axis in psychiatric and inflammatory disorders. *Front. Psychiatry* **9**, 44 (2018). 10.3389/fpsyt.2018.00044 PMC5859128 29593576

[27] Furness JB (2012). The enteric nervous system and neurogastroenterology. Nat. Rev. Gastroenterol. Hepatol..

[28] Mayer EA (2000). The neurobiology of stress and gastrointestinal disease. Gut.

[29] Hsu CC (2015). Depression and the risk of peptic ulcer disease: a Nationwide Population-based study. Medicine.

[30] Yang X-J, Jiang H-M, Hou X-H, Song J (2015). Anxiety and depression in patients with gastroesophageal reflux disease and their effect on quality of life. World J. Gastroenterol..

[31] Fond G (2014). Anxiety and depression comorbidities in irritable bowel syndrome (IBS): a systematic review and meta-analysis. Eur. Arch. Psychiatry Clin. Neurosci..

[32] Frolkis, A. D. et al. Depression increases the risk of inflammatory bowel disease, which may be mitigated by the use of antidepressants in the treatment of depression. *Gut*10.1136/gutjnl-2018-317182 (2018). 10.1136/gutjnl-2018-317182 30337374

[33] Richter JE (2004). Effect of Helicobacter pylori eradication on the treatment of gastro-oesophageal reflux disease. Gut.

[34] Yang J, Lee SH, Goddard ME, Visscher PM (2011). GCTA: a tool for genome-wide complex trait analysis. Am. J. Hum. Genet..

[35] Yang J (2012). Conditional and joint multiple-SNP analysis of GWAS summary statistics identifies additional variants influencing complex traits. Nat. Genet..

[36] Banda Y (2015). Characterizing race/ethnicity and genetic Ancestry for 100,000 subjects in the Genetic Epidemiology Research on Adult Health and Aging (GERA) Cohort. Genetics.

[37] Zhu Z (2018). Causal associations between risk factors and common diseases inferred from GWAS summary data. Nat. Commun..

[38] Watanabe K, Taskesen E, van Bochoven A, Posthuma D (2017). Functional mapping and annotation of genetic associations with FUMA. Nat. Commun..

[39] MacArthur J (2017). The new NHGRI-EBI Catalog of published genome-wide association studies (GWAS Catalog). Nucleic Acids Res..

[40] Bulik-Sullivan BK (2015). LD Score regression distinguishes confounding from polygenicity in genome-wide association studies. Nat. Genet..

[41] Zheng J (2017). LD Hub: a centralized database and web interface to perform LD score regression that maximizes the potential of summary level GWAS data for SNP heritability and genetic correlation analysis. Bioinformatics.

[42] Demontis D (2019). Discovery of the first genome-wide significant risk loci for attention deficit/hyperactivity disorder. Nat. Genet..

[43] Pardiñas AF (2018). Common schizophrenia alleles are enriched in mutation-intolerant genes and in regions under strong background selection. Nat. Genet..

[44] Otowa T (2016). Meta-analysis of genome-wide association studies of anxiety disorders. Mol. Psychiatry.

[45] Duncan LE (2017). Largest GWAS of PTSD (N = 20,070) yields genetic overlap with schizophrenia and sex differences in heritability. Mol. Psychiatry.

[46] Stahl EA (2019). Genome-wide association study identifies 30 loci associated with bipolar disorder. Nat. Genet..

[47] Grove J (2019). Identification of common genetic risk variants for autism spectrum disorder. Nat. Genet..

[48] Wray NR (2018). Genome-wide association analyses identify 44 risk variants and refine the genetic architecture of major depression. Nat. Genet..

[49] Marioni RE (2018). GWAS on family history of Alzheimer’s disease. Transl. Psychiatry.

[50] Nalls MA (2019). Identification of novel risk loci, causal insights, and heritable risk for Parkinson’s disease: a meta-analysis of genome-wide association studies. Lancet Neurol..

[51] Okbay A (2016). Genetic variants associated with subjective well-being, depressive symptoms, and neuroticism identified through genome-wide analyses. Nat. Genet..

[52] Hammerschlag AR (2017). Genome-wide association analysis of insomnia complaints identifies risk genes and genetic overlap with psychiatric and metabolic traits. Nat. Genet..

[53] Lane JM (2017). Genome-wide association analyses of sleep disturbance traits identify new loci and highlight shared genetics with neuropsychiatric and metabolic traits. Nat. Genet..

[54] Locke AE (2015). Genetic studies of body mass index yield new insights for obesity biology. Nature.

[55] Shungin D (2015). New genetic loci link adipose and insulin biology to body fat distribution. Nature.

[56] Nikpay M (2015). A comprehensive 1000 Genomes-based genome-wide association meta-analysis of coronary artery disease. Nat. Genet..

[57] Morris AP (2012). Large-scale association analysis provides insights into the genetic architecture and pathophysiology of type 2 diabetes. Nat. Genet..

[58] Okbay A (2016). Genome-wide association study identifies 74 loci associated with educational attainment. Nature.

[59] Finucane HK (2018). Heritability enrichment of specifically expressed genes identifies disease-relevant tissues and cell types. Nat. Genet..

[60] GTEx Consortium. (2015). The Genotype-Tissue Expression (GTEx) pilot analysis: multitissue gene regulation in humans. Science.

[61] Fehrmann RSN (2015). Gene expression analysis identifies global gene dosage sensitivity in cancer. Nat. Genet..

[62] Lee JJ (2018). Gene discovery and polygenic prediction from a genome-wide association study of educational attainment in 1.1 million individuals. Nat. Genet..

[63] Liu M (2019). Association studies of up to 1.2 million individuals yield new insights into the genetic etiology of tobacco and alcohol use. Nat. Genet..

[64] Zhu Z (2016). Integration of summary data from GWAS and eQTL studies predicts complex trait gene targets. Nat. Genet..

[65] McRae AF (2018). Identification of 55,000 replicated DNA methylation QTL. Sci. Rep..

[66] Roadmap Epigenomics C (2015). Integrative analysis of 111 reference human epigenomes. Nature.

[67] de Leeuw CA, Mooij JM, Heskes T, Posthuma D (2015). MAGMA: generalized gene-set analysis of GWAS data. PLOS Comput. Biol..

[68] Cai N (2020). Minimal phenotyping yields genome-wide association signals of low specificity for major depression. Nat. Genet..

[69] O’Connor LJ, Price AL (2018). Distinguishing genetic correlation from causation across 52 diseases and complex traits. Nat. Genet..

[70] Toyoshima O (2017). Decrease in PSCA expression caused by Helicobacter pylori infection may promote progression to severe gastritis. Oncotarget.

[71] Edgren G (2010). Risk of gastric cancer and peptic ulcers in relation to ABO blood type: a cohort study. Am. J. Epidemiol..

[72] Melzer D (2008). A Genome-Wide Association Study Identifies protein quantitative trait loci (pQTLs). PLOS Genet..

[73] Ikehara Y (2001). Polymorphisms of two fucosyltransferase genes (Lewis and Secretor genes) involving type I Lewis antigens are associated with the presence of anti-Helicobacter pylori IgG antibody. Cancer Epidemiol. Biomark. Prev..

[74] Magalhães A (2016). Muc5ac gastric mucin glycosylation is shaped by FUT2 activity and functionally impacts Helicobacter pylori binding. Sci. Rep..

[75] Azad MB, Wade KH, Timpson NJ (2018). FUT2 secretor genotype and susceptibility to infections and chronic conditions in the ALSPAC cohort. Wellcome Open Res..

[76] McGuckin MA (2007). Muc1 mucin limits both Helicobacter pylori colonization of the murine gastric mucosa and associated gastritis. Gastroenterology.

[77] Niv Y (2015). Helicobacter pylori and gastric mucin expression: a systematic review and meta-analysis. World J. Gastroenterol..

[78] Boltin D, Niv Y (2013). Mucins in gastric cancer—an update. J. Gastrointest. Dig. Syst..

[79] Asano N (2016). Cdx2 expression and intestinal metaplasia induced by H. pylori infection of gastric cells is regulated by NOD1-mediated innate immune responses. Cancer Res..

[80] Lenka A, Arumugham SS, Christopher R, Pal PK (2016). Genetic substrates of psychosis in patients with Parkinson’s disease: a critical review. J. Neurol. Sci..

[81] Murrough JW, Yaqubi S, Sayed S, Charney DS (2015). Emerging drugs for the treatment of anxiety. Expert Opin. Emerg. Drugs.

[82] Levine DM (2013). A genome-wide association study identifies new susceptibility loci for esophageal adenocarcinoma and Barrett’s esophagus. Nat. Genet..

[83] Fröhlich H (2019). Gastrointestinal dysfunction in autism displayed by altered motility and achalasia in Foxp1+/− mice. Proc. Natl Acad. Sci..

[84] Avetisyan M, Schill EM, Heuckeroth RO (2015). Building a second brain in the bowel. J. Clin. Invest..

[85] Fass R, Tougas G (2002). Functional heartburn: the stimulus, the pain, and the brain. Gut.

[86] Lagoo J, Pappas TN, Perez A (2014). A relic or still relevant: the narrowing role for vagotomy in the treatment of peptic ulcer disease. Am. J. Surg..

[87] Kim SY (2018). Bidirectional association between gastroesophageal reflux disease and depression: two different nested case-control studies using a national sample cohort. Sci. Rep..

[88] Wu Y (2019). Genome-wide association study of medication-use and associated disease in the UK Biobank. Nat. Commun..

[89] van Rheenen, W., Peyrot, W. J., Schork, A. J., Lee, S. H. & Wray, N. R. Genetic correlations of polygenic disease traits: from theory to practice. *Nat. Rev. Genet.*10.1038/s41576-019-0137-z (2019). 10.1038/s41576-019-0137-z 31171865

[90] Kamolz T, Velanovich V (2002). Psychological and emotional aspects of gastroesophageal reflux disease. Dis. Esophagus.

[91] MartÍN-Merino E, RuigÓMez A, GarcÍA RodrÍGuez LA, Wallander MA, Johansson S (2010). Depression and treatment with antidepressants are associated with the development of gastro-oesophageal reflux disease. Aliment. Pharmacol. Ther..

[92] Huang WS (2018). Use of proton pump inhibitors and risk of major depressive disorder: a nationwide population-based study. Psychother. Psychosom..

[93] Momen NC (2020). Association between mental disorders and subsequent medical conditions. N. Engl. J. Med..

[94] Nojkov B (2008). The influence of co-morbid IBS and psychological distress on outcomes and quality of life following PPI therapy in patients with gastro-oesophageal reflux disease. Aliment. Pharmacol. Ther..

[95] Khandaker GM, Dantzer R, Jones PB (2017). Immunopsychiatry: important facts. Psychol. Med..

[96] Munafò MR, Tilling K, Taylor AE, Evans DM, Davey Smith G (2017). Collider scope: when selection bias can substantially influence observed associations. Int. J. Epidemiol..

[97] Bycroft C (2018). The UK Biobank resource with deep phenotyping and genomic data. Nature.

[98] Yengo L (2018). Meta-analysis of genome-wide association studies for height and body mass index in ∼700,000 individuals of European ancestry. Hum. Mol. Genet..

[99] Chang CC (2015). Second-generation PLINK: rising to the challenge of larger and richer datasets. Gigascience.

[100] Mowat C (2011). Guidelines for the management of inflammatory bowel disease in adults. Gut.

[101] Santos R (2017). A comprehensive map of molecular drug targets. Nat. Rev. Drug Discov..

[102] Manichaikul A (2010). Robust relationship inference in genome-wide association studies. Bioinformatics.

[103] Wray NR, Gottesman II (2012). Using summary data from the danish national registers to estimate heritabilities for schizophrenia, bipolar disorder, and major depressive disorder. Front. Genet..

[104] Falconer DS (1965). The inheritance of liability to certain diseases, estimated from the incidence among relatives. Ann. Hum. Genet..

[105] Reich T, James JW, Morris CA (1972). The use of multiple thresholds in determining the mode of transmission of semi-continuous traits*. Ann. Hum. Genet..

[106] Loh P-R, Kichaev G, Gazal S, Schoech AP, Price AL (2018). Mixed-model association for biobank-scale datasets. Nat. Genet..

[107] Lloyd-Jones LR, Robinson MR, Yang J, Visscher PM (2018). Transformation of summary statistics from linear mixed model association on all-or-none traits to odds ratio. Genetics.

[108] Pruim RJ (2010). LocusZoom: regional visualization of genome-wide association scan results. Bioinformatics.

[109] Jiang W, Yu W (2016). Power estimation and sample size determination for replication studies of genome-wide association studies. BMC Genomics.

[110] Bulik-Sullivan B (2015). An atlas of genetic correlations across human diseases and traits. Nat. Genet..

[111] Finucane HK (2015). Partitioning heritability by functional annotation using genome-wide association summary statistics. Nat. Genet..

[112] Kent WJ (2002). The human genome browser at UCSC. Genome Res..

[113] Lindblad-Toh K (2011). A high-resolution map of human evolutionary constraint using 29 mammals. Nature.

[114] The EPC (2012). An integrated encyclopedia of DNA elements in the human genome. Nature.

[115] Võsa, U. et al. Unraveling the polygenic architecture of complex traits using blood eQTL metaanalysis. Preprint at *bioRxiv*10.1101/447367 (2018).

[116] Qi T (2018). Identifying gene targets for brain-related traits using transcriptomic and methylomic data from blood. Nat. Commun..

[117] Subramanian A (2005). Gene set enrichment analysis: a knowledge-based approach for interpreting genome-wide expression profiles. Proc. Natl Acad. Sci..

[118] Liberzon A (2015). The molecular signatures database hallmark gene set collection. Cell Syst..

[119] Benjamini Y, Hochberg Y (1995). Controlling the false discovery rate: a practical and powerful approach to multiple test. J. R. Stat. Soc. Ser. B.

[120] Burgess S, Butterworth A, Thompson SG (2013). Mendelian randomization analysis with multiple genetic variants using summarized data. Genet. Epidemiol..

[121] Bowden J, Davey Smith G, Burgess S (2015). Mendelian randomization with invalid instruments: effect estimation and bias detection through Egger regression. Int. J. Epidemiol..

[122] Bowden J, Davey Smith G, Haycock PC, Burgess S (2016). Consistent estimation in Mendelian randomization with some invalid instruments using a weighted median estimator. Genet. Epidemiol..

[123] Verbanck M, Chen C-Y, Neale B, Do R (2018). Detection of widespread horizontal pleiotropy in causal relationships inferred from Mendelian randomization between complex traits and diseases. Nat. Genet..

[124] Davey Smith G (2019). STROBE-MR: Guidelines for strengthening the reporting of Mendelian randomization studies. PeerJ Prepr..

[125] Robin X (2011). pROC: an open-source package for R and S+ to analyze and compare ROC curves. BMC Bioinform..

