# Supplementary material for: GWAS of peptic ulcer disease implicates Helicobacter pylori infection, other gastrointestinal disorders and depression
Source: Nat Commun. 2021 Feb 19;12:1146. doi: 10.1038/s41467-021-21280-7 (PMC7895976; doi:10.1038/s41467-021-21280-7)
Supplement: Supplementary file 1 — Supplementary Information [file 41467_2021_21280_MOESM1_ESM.pdf]

SUPPLEMENTARY NOTE, TABLES & FIGURES

GWAS of peptic ulcer disease implicates Helicobacter pylori infection, other gastrointestinal disorders and depression

Yeda Wu*, Graham K. Murray, Enda M. Byrne, Julia Sidorenko, Peter M. Visscher, Naomi R. Wray*

* Correspondence should be addressed to Y.W. (yeda.wu@uq.edu.au) N.R.W. (naomi.wray@uq.edu.au)

Table of Contents

Supplementary Notes 4

Supplementary Note 1 4
Supplementary Note 2 4
Supplementary Note 3 4
Supplementary Note 4 5
Supplementary Note 5 5

Supplementary Tables 7

Supplementary Table 1 7
Supplementary Table 2 8
Supplementary Table 3 10
Supplementary Table 4 11
Supplementary Table 5 13
Supplementary Table 6 14
Supplementary Table 7 15
Supplementary Table 8 16
Supplementary Table 9 17
Supplementary Table 10 19
Supplementary Table 11 21
Supplementary Table 12 23
Supplementary Table 13 24
Supplementary Table 14 25
Supplementary Table 15 26
Supplementary Table 16 28
Supplementary Table 17 29
Supplementary Table 18 30
Supplementary Table 19 31
Supplementary Table 20 32
Supplementary Table 21 33
Supplementary Table 22 34
Supplementary Table 23 35
Supplementary Table 24 36
Supplementary Table 25 37
Supplementary Table 26 38
Supplementary Table 27 39
Supplementary Table 28 40
Supplementary Table 29 41

Supplementary Figures 42

Supplementary Fig. 1 42
Supplementary Fig. 2 43
Supplementary Fig. 3 44
Supplementary Fig. 4 45
Supplementary Fig. 5 46
Supplementary Fig. 6 47
Supplementary Fig. 7 50
Supplementary Fig. 8 51
Supplementary Fig. 9 55
Supplementary Fig. 10 57
Supplementary Fig. 11 59
Supplementary Fig. 12 60

Supplementary Fig. 13 61
Supplementary Fig. 14 62
Supplementary Fig. 15 63
Supplementary Fig. 16 64

References 65

Supplementary Note

Supplementary Note 1: Detailed instruction for Panel b and c of Fig. 1.

Panel b explanation. Cells represent the ratio of the odds of being a disease case for the column disease in those from each row and the odds of each row disease cases in unrelated European-ancestry individuals. For example, the odds of person in the UKB with a GORD phenotype is 12 fold higher in those with PUD, compared to those without PUD. However, GORD is more common than PUD (11.7% vs. 3.5%) and so the odds of PUD is less common than in those without GORD, odds 0.77. **Panel c explanation.** Taking PUD as an example index disease. There are 7,545 PUD cases without any comorbidity for GORD, IBS and IBD. Among the GORD, IBS and PUD cases, we first removed individuals with at least two of the three diseases, e.g., there are 34,729 individuals who have a GORD diagnosis and not IBS or IBD, amongst whom 3,124 individuals also have a PUD diagnosis. Similarly, there are 16,337 individuals who have a IBS diagnosis but not GORD or IBD, amongst whom 743 individuals also have a PUD diagnosis. Lastly, and there are 3,839 individuals who have an IBD diagnosis but not GORD or IBS, among whom 201 individuals also have a PUD diagnoses. We then used a two proportion *Z* test to test which disease is more prone to be comorbid with PUD by comparing the proportion in pairs (i.e., 3124/34729, 743/16337, 201/3839).

Supplementary Note 2: Discussion of SNP-based heritability and genetic correlation of subgroup phenotypes for each of PUD, GORD and IBS.

A previous study reported rs10512344 to be the only SNP associated at the level of genome-wide significance (reported P = 3.6E-8) in females with IBS using UKB self-reported illness (Data Field: 20003) data[1]. However, the P value for this SNP in our analyses is 5.0E-5 (4.4E-7 in females, **Supplementary Fig. 10b**). Given that they used a self-report phenotype while we used a combination of self-reported, hospital admission and primary care diseases diagnoses, we conducted a sensitivity analysis. We first removed individuals with diagnosis records from at least two resources and identified individuals with a diagnosis record from only one resource. For each of the IBS, PUD and GORD, We then regenerated three subgroup phenotypes using cases from one-resource diagnosis record and controls from the original phenotype (**Supplementary Fig. 10a**). We conducted GWAS analyses (**Methods**) and investigated the summary statistics for rs10512344 among the GWAS analyses of the original IBS phenotype and the three IBS subgroup phenotypes (**Supplementary Fig. 10b**). We also investigated the SNP-based heritability and genetic correlation among the three subgroup phenotypes for each of the IBS, PUD and GORD (**Supplementary Fig. 10c-d** and **Supplementary Table 8-9**). Results showed that the three subgroup phenotypes for each of IBS, PUD and GORD are highly genetically correlated with each other (**Supplementary Fig. 10d** and **Supplementary Table 9**) (and from simulation and theory this overlap is not a reflection of the use of shared controls[2]). Our results do not support a robust association of rs10512344 with IBS.

Supplementary Note 3: Sensitivity analysis for SNP-based heritability and genetic correlation analyses.

As a sensitivity analysis, SNP-based heritability (h^2_{SNP}) and genetic correlation (r_g) analyses were conducted for PUD, GORD, IBS and IBD using the phenotypes generated after excluding individuals with more than one of the four gastrointestinal (GI) disorders (defined as sensitivity analysis phenotypes). The number of overlapped individuals for PUD, GORD, IBS and IBD case are in **Supplementary Fig. 9a**. As expected, the h^2_{SNP} estimates on the observed scale for these disorders were lower due to the excluded individuals from the cases of original phenotypes but still significantly different from zero (**Supplementary Fig. 9b**); conversion to liability scale is difficult after case exclusion as it contravenes underlying assumptions of the transformation. We then calculated the r_g within sensitivity analysis phenotypes, between sensitivity analysis phenotypes with traits from LD Hub and nine psychiatric and neurologic diseases from published studies (**Supplementary Fig. 9c**) (genetic correlations are robust to case/control ascertainment strategies[2]). The r_g within sensitivity analysis phenotypes showed high r_g among PUD, GORD, IBS and all low non-statistically significant r_g with IBD. The r_g between GORD and PUD is 0.38 (SE = 0.08, P = 3.6E-6) and the r_g between GORD and IBS is 0.47 (SE = 0.08, P = 3.4E-10), which are lower to the original results. The r_g between PUD and IBS is 0.25 (SE = 0.12, P = 0.034), of which the original r_g is 0.49 (SE = 0.08, P = 2.0E-10). As shown in **Supplementary Fig. 9d**, the number of overlapped individuals for PUD and IBS is 1,740, however, we over-removed 4,751 individuals for PUD and

6,323 individuals for IBS due to the overlap with the other two GI disorders (GORD and IBD, shown in **Supplementary Fig. 9a**). The number of total PUD and IBS cases is 16,666 and 29,524 and these over-removed individuals occupy ~1/4 for PUD cases and ~1/5 for IBS cases, resulting in reducing power to estimate the r_g. Thus we only removed the only 1,740 overlapped individuals for PUD and IBS and re-calculated the r_g between PUD and IBS, as shown in **Supplementary Fig. 9e**. The r_g is 0.33 (SE = 0.09, P = 3.0E-4). PUD, GORD and IBS sensitivity analyses phenotypes showed statistically significantly r_g with depressive symptoms while there is no statistically significant r_g between IBD and depressive symptoms. The h^2_{SNP} for sensitivity analyses phenotypes are in **Supplementary Table 6**. The r_g within sensitivity analysis phenotypes and between sensitivity analysis phenotypes and traits from the nine published psychiatric and neurologic studies are in **Supplementary Table 7** and **11**. The r_g between sensitivity analysis phenotypes with traits from LD Hub are in **Supplementary Data 4**.

Supplementary Note 4: Sensitivity analysis for Mendelian Randomization between major depression and PG+M.

Given the statistically significant results between MD and PG+M from bidirectional GSMR analyses, we also conducted bidirectional MR analyses between MD and PG+M using the TwoSampleMR package (https://github.com/MRCIEU/TwoSampleMR). For each of MD and PG+M GWAS summary statistics, we first generated the independent loci using PLINK(v1.90b)[3] (--clump-p1 5.0E-8 –clump-p2 5.0E-8 –clump-r2 0.01 –clump-kb 1000) and the genotype data (8,545,065 SNPs with MAF > 0.01) of 20,000 randomly sampled unrelated European individuals from UKB as a LD reference. Only the most significant SNP across the MHC region was retained. For each of the genetic instruments (i.e., SNPs), we extracted the allele, effect size, standard error and P value from the exposure GWAS summary statistics. We also extracted the corresponding information from the outcome GWAS summary statistics for these genetic instruments. If a SNP was unavailable in the outcome GWAS summary statistics, we identified proxy SNP with a minimum LD r^2 = 0.7. For each direction of potential influence, we combined MR estimates using inverse variance-weighted (IVW)[4] analysis, which essentially translates to a weighted regression of SNP-outcome effects on SNP-exposure effects where the intercept is constrained to zero. The IVW method will return an unbiased estimate if there is no or balanced horizontal pleiotropy. To account for this, we compared results from IVW method with results from MR Egger[5] and weighted median method[6], from which the estimates are known to be relatively robust to horizontal pleiotropy, though at the cost of reduced statistical power. To assess robustness of significant results, we also conducted the MR Egger intercept test for horizontal pleiotropy. We also applied MR-PRESSO[7] (Pleiotropy Residual Sum and Outlier) to detect and correct for any outliers reflecting likely pleiotropic biases for all reported results. The IVW results showed bidirectionally statistically significant results, of which the pattern is similar as the GSMR results (**Supplementary Fig. 15**). The MR-Egger intercept test showed no statistical significance, suggesting that there is no horizontal pleiotropy (**Supplementary Table 22**). There is no outliers being removed after MR-PRESSO analyses. LCV method[8] is designed to separate confounders from causality and hence is more likely to differ from MR where there is a unidirectional MR result. We used the LCV method to explore the relationship between MD and PG+M, following the instructions from https://github.com/lukejoconnor/LCV. Briefly, we used the munged file from LDSC for MD and PG+M, together with the provided LD score from 1000 Genomes Europeans data (eur_w_ld_chr, MHC region removed), as input. After selecting SNPs with MAF > 0.05 and sorted the SNPs from GWAS summary statistics by genomic region, the RunLCV() function was used for analysis. As expected ,the genetic causality proportion is not significant for PG+M and MD because of the strong bidirectional significance (**Supplementary Table 24**).

Supplementary Note 5: Sensitivity analysis for Mendelian Randomization between major depression and depression-removed sensitivity GI phenotypes.

As sensitivity analyses, we removed the depression cases (the combined cases from the UKB eight depression phenotypes) from the five GI disorder phenotypes (defined as GI-DepComRMV phenotypes) and conducted GWAS analyses. We repeated LDSC genetic correlation and MR analyses between major depression and these five GI-DepComRMV phenotypes. All genetic correlation results retained a pattern that did not change our interpretation of the original results (**Supplementary Table 25**). The MR results showed similar pattern results for four GI-DepComRMV phenotypes (GORD, PG+M, IBS and IBD), although the major depression and PUD-DepComRMV became non-significant (**Supplementary Table 26**). These analyses removed a very high number of cases and controls based on the combined eight depression phenotype cases from PUD and hence the magnitude of the standard errors of the estimates from these sensitivity analyses were large. To gain further insight, we regenerated eight PUD sensitivity phenotypes after removing the cases of each of the eight

depression phenotypes from PUD in turn and repeated MR analyses. The MR results were significant for seven of them, as shown in **Supplementary Table 27**. The MR result were non-significant when cases and controls were removed based on the GPpsy-seen a GP for nerves, anxiety, tension or depression in which only 59% of cases and 65% of controls were retained for analysis.

Supplementary Tables

Supplementary Table 1. Case definition and number of cases for each phenotype in UK Biobank.

Phenotypes	UKB data field	UKB data field description	UKB data coding for case definition	Number of cases
PUD	131591	Source of report of K25 (gastric ulcer)	20: Death register only<br>21: Death register and other source(s)<br>30: Primary care only<br>31: Primary care and other source(s)<br>40: Hospital admissions data only<br>41: Hospital admissions data and other source(s)<br>50: Self-report only<br>51: Self-report and other source(s)	16666
	131593	Source of report of K26 (duodenal ulcer)		
	131595	Source of report of K27 (peptic ulcer, site unspecified)		
	131597	Source of report of K28 (gastrojejunal ulcer)		
GORD	131585	Source of report of K21 (gastro-oesophageal reflux disease)		54854
IBD	131627	Source of report of K50 (crohn's disease [regional enteritis])		7045
	131629	Source of report of K51 (ulcerative colitis)		
IBS (removed IBD cases)	131639	Source of report of K58 (irritable bowel syndrome)		28518
PG$_+$M	131585	Source of report of K21 (gastro-oesophageal reflux disease)		90175
	131591	Source of report of K25 (gastric ulcer)		
	131593	Source of report of K26 (duodenal ulcer)		
	131595	Source of report of K27 (peptic ulcer, site unspecified)		
	131597	Source of report of K28 (gastrojejunal ulcer)		
	41272	Operative procedures - OPCS4	G24: Antireflux operations<br>G25: Revision of antireflux operations	
	6154	Medication for pain relief, constipation, heartburn	4:Ranitidine (e.g. Zantac)<br>5:Omeprazole (e.g. Zanprol)	
	20003	Treatment/medication code	Supplementary Table 1	

Supplementary Table 2. Medications for peptic ulcer disease (PUD) and gastro-oesophageal reflux disease (GORD) in UK Biobank.

Category	Coding	Active ingredient	ATC code*
Maalox tablet	1140865358	Aluminum Hydroxide, Magnesium Hydroxide, Simethicone	A02A
Mucogel suspension	1140865368	Aluminum Hydroxide, Magnesium Hydroxide	A02A
Co-magaldrox	1140881318	Aluminum Hydroxide, Magnesium Hydroxide	A02A
Magnesium trisilicate	1140881324	Magnesium Trisilicate	A02A
Milk of magnesia suspension	1140881550	Magnesium Hydroxide	A02AA
Magnesium carbonate	1140881320	Magnesium Carbonate	A02AA01
Magnesium hydroxide	1140881330	Magnesium Hydroxide	A02AA04
Rennie duo oral suspension	1141166086	Calcium Carbonate	A02AC01
Asilone liquid	1140881422	Magnesium Oxide, Aluminum Hydroxide, Dimethicone	A02AF
Maalox plus suspension	1140865366	Aluminum Hydroxide, Magnesium Hydroxide	A02AF02
Gastrocote liquid	1140881414	Sodium Bicarbonate, Aluminum Hydroxide, Alginic Acid, Magnesium Trisilicate	A02AX
Gastrocote s/f liquid	1140928346	Sodium Bicarbonate, Aluminum Hydroxide, Alginic Acid, Magnesium Trisilicate	A02AX
Peptac liquid	1141168752	Sodium Bicarbonate, Calcium Carbonate, Alginic Acid	A02AX
Gavilast-p 75mg tablet	1141188426	Ranitidine	A02BA
Cimetidine	1140865426	Cimetidine	A02BA01
Tagamet 100 tablet	1140909500	Cimetidine	A02BA01
Ranitidine	1140879406	Ranitidine	A02BA02
Zantac 75 tablet	1140916980	Ranitidine	A02BA02
Famotidine	1140865608	Famotidine	A02BA03
Pepcid ac indigestion tablet	1140909496	Famotidine	A02BA03
Nizatidine	1140865618	Nizatidine	A02BA04
Misoprostol	1140865628	Misoprostol	A02BB01
Omeprazole	1140865634	Omeprazole	A02BC01
Losec 10mg capsule	1140909578	Omeprazole	A02BC01
Pantoprazole	1140929012	Pantoprazole	A02BC02
Protium 20mg e/c tablet	1141164616	Pantoprazole	A02BC02
Lansoprazole	1140864752	Lansoprazole	A02BC03
Zoton 15mg capsule	1140923688	Lansoprazole	A02BC03
Rabeprazole sodium	1141168584	Rabeprazole	A02BC04

Pariet 10mg e/c tablet	1141168590	Rabeprazole	A02BC04
Esomeprazole	1141177526	Esomeprazole	A02BC05
Nexium 20mg tablet	1141177532	Esomeprazole	A02BC05
Acidex oral suspension	1141172224	Sodium Bicarbonate, Calcium Carbonate, Alginic Acid	A02BX
Sucralfate	1140865536	Sucralfate	A02BX02
Antepsin 1g tablet	1140865538	Sucralfate	A02BX02
Gaviscon liquid	1140865354	Sodium Bicarbonate, Calcium Carbonate, Alginic Acid	A02BX13
Topal tablet	1140865370	Aluminum Hydroxide, Magnesium Carbonate, Alginic Acid	A02BX13

[*] The Supplementary Data 1 of Wu *et al.*[9] provides UKB medication classification based on Anatomical Therapeutic Chemical (ATC) Classification System[10] and we extracted medications for PUD and GORD (the first two ATC level: A02) as listed above.

Supplementary Table 3. Genome-wide significant SNPs associated with inflammatory bowel disease (IBD) in UK Biobank from BOLT-LMM[11] association analyses.

SNP	CHR.	BP	A1/A2	A1 Frequency	OR	P	Reported SNP*	PMID
rs148844907	6	31628397	T/A	0.99	0.47	9.7E-41	rs17207986	20848476
rs11581607	1	67707690	G/A	0.93	1.51	2.2E-24	rs11209026	21102463
rs4655529	1	67698909	C/T	0.69	0.84	1.0E-21	rs7517847	17435756
rs2836878	21	40465534	G/A	0.73	1.21	2.1E-21	rs2836878	18758464
rs10737482	1	20173858	T/C	0.39	0.86	3.4E-16	rs6426833	19915572
rs3024505	1	206939904	G/A	0.85	0.84	8.7E-15	rs3024505	21102463
rs6017342	20	43065028	A/C	0.48	0.88	1.6E-14	rs6017342	19915572
rs905634	1	200884985	C/T	0.69	1.14	7.8E-13	rs11584383	18587394
rs6671847	1	161478810	G/A	0.49	1.13	1.6E-12	rs1801274	19915573
rs1297261	21	16812623	T/C	0.57	1.13	1.6E-12	rs1736020	21102463
rs10761659	10	64445564	A/G	0.46	0.89	9.7E-12	rs10761659	21102463
rs11403745	10	101282604	C/CA	0.48	1.12	2.1E-11	rs4409764	21102463
rs35788599	12	68476749	G/C	0.62	0.89	3.2E-11	rs7134599	21297633
rs7936312	11	76293726	G/T	0.52	0.90	6.7E-11	rs7927997	21102463
rs10799837	1	20135612	G/A	0.43	1.12	1.6E-10	rs1317209	20228799
9:5057580	9	5057580	CTTT/C	0.56	0.90	5.4E-10	rs10758669	21102463
rs4551125	5	40438684	G/A	0.39	0.90	7.5E-10	rs11742570	21102463
rs59998884	21	45618114	T/C	0.40	1.11	1.0E-09	rs2838519	21102463
rs2066847	16	50763778	G/GC	0.98	0.71	1.7E-09	rs2066847	20570966
rs17264332	6	138005515	A/G	0.78	0.89	2.5E-09	rs6920220	21297633
rs3806306	1	20143100	A/G	0.62	1.11	2.6E-09	rs3806308	19122664
rs55722650	5	131607300	C/T	0.58	0.90	4.5E-09	rs12521868	21102463
rs6961243	7	107521404	G/A	0.59	1.11	5.0E-09	rs886774	19915572
rs1521186	1	151784547	G/A	0.55	0.91	7.8E-09	-	-
rs13384671	2	182311594	A/G	0.69	0.90	9.0E-09	rs6740847	28067908
5:17098189	5	17098189	AT/A	0.78	1.13	1.1E-08	-	-
rs73370726	10	90852873	C/T	0.90	1.19	1.5E-08	-	-
rs12720356	19	10469975	A/C	0.90	0.86	2.3E-08	rs12720356	21102463
rs12568930	1	22702231	T/C	0.82	1.14	2.6E-08	rs7524102	21297633
rs142738614	7	128577914	C/CGCGGG	0.55	0.91	3.3E-08	rs4728142	21297633
rs10188217	2	61217542	T/C	0.48	0.91	4.3E-08	rs10181042	21102463

* The associations between SNPs from "Reported SNP" column and IBD have been reported by other studies (corresponding PMID column). These SNPs are either same as IBD-associated SNPs in our UK Biobank analysis or in linkage disequilibrium with our IBD-associated SNPs in UK Biobank. For the detailed statistics for those reported SNPs, please refer to **Supplementary Data 1**.

Supplementary Table 4. Genome-wide significant SNPs associated with GORD and PG$_{+}$M in the UK Biobank from BOLT-LMM[11] association analyses.

Digestion phenotypes	SNP[*]	CHR.	BP	A1/A2	A1 frequency	OR[†]	P	Digestive diseases pleiotropy[‡]	Mental health pleiotropy[‡]
GORD	rs2523589	6	31327334	G/T	0.50	0.95	4.6E-13	-	§
	rs967823	17	50317276	A/G	0.61	0.96	1.6E-10	-	-
	rs1430788	2	67868412	C/T	0.31	1.04	8.4E-09	-	-
PG$_{+}$M	rs200964	6	27866943	G/C	0.81	1.05	3.0E-13	-	§
	rs967823	17	50317276	A/G	0.61	0.96	2.4E-12	-	-
	rs10500661	11	6273744	T/C	0.80	0.96	9.7E-12	-	-
	19:18793695	19	18793695	AAAAG/A	0.67	1.04	2.6E-11	Barrett's esophagus, Esophageal adenocarcinoma	-
	rs2861694	2	67845739	A/G	0.31	1.04	1.0E-10	-	-
	rs12631337	3	50198537	A/G	0.45	1.03	1.5E-10	-	SCZ, depressive symptoms and depressed affect
	rs61787782	1	98310239	A/G	0.78	0.96	8.1E-10	-	SCZ, ADHD, ASD, anorexia nervosa, etc.
	2:100485494	2	100485494	CCCTCTG/C	0.64	1.03	1.9E-09	-	-
	rs10641969	4	140940097	T/TCAA	0.62	1.03	3.1E-09	-	-
	rs62435650	7	1823265	A/G	0.80	1.04	3.9E-09	-	Depression, depressed affect and BIP
	rs12064884	1	66364651	A/G	0.50	1.03	5.2E-09	-	ADHD, stress-related disorders, etc.
	rs13097265	3	70943143	G/A	0.71	0.97	7.5E-09	Barrett's esophagus, Esophageal adenocarcinoma	-

	rs1873914	12	56379427	G/C	0.58	1.03	4.8E-08	-	Anorexia nervosa

* Locus zoom plot for SNPs are in Supplementary **Fig. 5-6**.
† Odds ratio (OR) is for risk of A1 allele compared to A2 allele.
‡ We only annotated SNPs if there are SNPs reported associated with either mental health-related traits or digestive diseases from GWAS Catalog[12] in linkage disequilibrium with our UKB digestion SNPs (see **Methods** and **Supplementary Data 1** for detailed description). "§" represents that SNP is within MHC region. For SNPs within MHC region see **Supplementary Data 1** for details given the complexity of MHC region.
Abbreviations: ASD: Autism spectrum disorder; BIP: Bipolar disorder; SCZ: Schizophrenia; ADHD: Attention deficit hyperactivity disorder.

Supplementary Table 5. GERA peptic ulcer and irritable bowel syndrome (IBS) GWAS summary statistics for SNPs associated with UKB peptic ulcer diseases and IBS (P < 5e-8).

Trait	SNP	CHR.	BP	UKB statistics				Power for replication at GERA sample size			GERA statistics			
				**A1/A2**	**A1 frequency**	**OR***	**P**	**Alpha level**	**No. of cases/controls**	**Power†**	**A1/A2**	**A1 frequency**	**OR***	**P**
PUD	rs681343	19	49206462	C/T	0.49	0.92	1.9E-15	0.05/8	1004/60843	0.26	C/T	0.51	0.84	5E-4
	rs2976388	8	143760256	G/A	0.58	1.09	1.8E-14			0.27	G/A	0.57	1.07	0.12
	rs10500661	11	6273744	T/C	0.80	0.90	4.1E-14			0.29	T/C	0.79	0.90	0.08
	rs147048677	1	155161794	C/T	0.94	0.86	9.0E-12			0.20	C/T	0.95	0.87	0.20
	rs78459074	11	1029905	A/G	0.89	1.12	2.6E-10			0.17	-	-	-	-
	rs34074411	17	39867248	C/T	0.56	0.93	2.6E-10			0.19	-	-	-	-
	rs687621	9	136137065	A/G	0.68	1.08	1.3E-09			0.18	A/G	0.66	1.13	0.02
	rs9581957	13	28557889	C/T	0.68	0.93	3.6E-09			0.17	C/T	0.68	0.96	0.41
IBS	rs7947502	11	112909396	C/T	0.41	1.05	2.5E-08	0.05/2	3359/58488	0.48	C/T	0.41	1.04	0.14
	rs2523599	6	31241092	C/G	0.39	0.95	4.4E-08			0.51	-	-	-	-

* Odds ratio (OR) is for risk of A1 allele compared to A2 allele.

† Power to detect SNPs with statistical significance at an alpha level and the GERA sample size. The power for each SNP were calculated using RPower package[13]. Since the average power for the PUD SNPs is 20%, identifying 1 in 6 available for testing in line with these power calculations.

Supplementary Table 6. SNP-based heritability estimates and other parameters from LD score regression analysis[14].

Digestion Phenotypes	λ_{GC}*	Intercept (s.e.)	h2_observed (s.e.)	Population lifetime risk†	h2_liability (s.e.)
PUD	1.10	1.01 (0.007)	0.01 (0.001)	0.037	0.06 (0.007)
GORD	1.25	1.02 (0.008)	0.03 (0.002)	0.120	0.08 (0.004)
$PG_{+}M$	1.43	1.04 (0.008)	0.05 (0.002)	0.198	0.09 (0.003)
IBS	1.15	1.00 (0.007)	0.02 (0.001)	0.063	0.06 (0.005)
IBD	1.11	1.03 (0.008)	0.01 (0.002)	0.015	0.11 (0.016)
PUD (RO)‡	1.05	1.01 (0.006)	0.006 (0.001)	0.023	0.046 (0.008)
GORD (RO)	1.20	1.02 (0.008)	0.022 (0.001)	0.095	0.066 (0.004)
IBS (RO)	1.05	1.00 (0.007)	0.008 (0.001)	0.046	0.038 (0.006)
IBD (RO)	1.10	1.03 (0.008)	0.008 (0.002)	0.012	0.096 (0.018)
PUD (RTO)	1.10	1.01 (0.007)	0.009 (0.001)	0.033	0.050 (0.007)
IBS (RTO)	1.10	1.01 (0.007)	0.013 (0.001)	0.059	0.052 (0.005)

* The calculation for λ_{GC} is from LD score regression.

† We used the proportion of sample that are cases as the estimates of population lifetime.

‡ We note that the case ascertainment for RO and RTO ascertainment means that assumptions underlying conversion of SNP-based heritabilities to the liability scale may be violated.

Abbreviation: s.e.: standard error; RO: remove individuals with more than one of PUD, GORD, IBS and IBD disorders; RTO: remove the overlapped individuals with both PUD and IBS.

Supplementary Table 7. Genetic correlation estimates for each pair of the five original digestion phenotypes and each pair of the five sensitivity digestion phenotypes from bivariate LD score regression analysis[15]. (see **Fig. 4b** and **Supplementary Fig. 9c$_1$**)

Trait1	Trait2	rg	se	p	gcov int[*]	gcov int se
PUD	GORD	0.65	0.052	4.9E-36	0.12	0.005
PUD	PG$_{+}$M	0.76	0.036	5.1E-101	0.40	0.006
PUD	IBS	0.49	0.077	2.0E-10	0.04	0.005
PUD	IBD	0.07	0.084	3.7E-01	0.03	0.006
GORD	PUD	0.65	0.052	4.9E-36	0.12	0.005
GORD	PG$_{+}$M	0.97	0.008	0.0E+00	0.77	0.007
GORD	IBS	0.65	0.045	1.1E-46	0.10	0.005
GORD	IBD	0.19	0.056	9.0E-04	0.02	0.005
PG$_{+}$M	PUD	0.76	0.036	5.1E-101	0.40	0.006
PG$_{+}$M	GORD	0.97	0.008	0.0E+00	0.77	0.007
PG$_{+}$M	IBS	0.64	0.040	1.4E-57	0.11	0.006
PG$_{+}$M	IBD	0.19	0.048	1.0E-04	0.03	0.006
IBS	PUD	0.49	0.077	2.0E-10	0.04	0.005
IBS	GORD	0.65	0.045	1.1E-46	0.10	0.005
IBS	PG$_{+}$M	0.64	0.040	1.4E-57	0.11	0.006
IBS	IBD	0.02	0.070	7.6E-01	-0.02	0.005
IBD	PUD	0.07	0.084	3.7E-01	0.03	0.006
IBD	GORD	0.19	0.056	9.0E-04	0.02	0.005
IBD	PG$_{+}$M	0.19	0.048	1.0E-04	0.03	0.006
IBD	IBS	0.02	0.070	7.6E-01	-0.02	0.005
PUD (RO)[†]	GORD (RO)	0.38	0.082	3.6E-06	-0.05	0.005
PUD (RO)	IBS (RO)	0.25	0.117	3.4E-02	-0.03	0.005
PUD (RO)	IBD (RO)	-0.12	0.120	3.4E-01	-0.02	0.005
GORD (RO)	PUD (RO)	0.38	0.082	3.6E-06	-0.05	0.005
GORD (RO)	IBS (RO)	0.47	0.075	3.4E-10	-0.08	0.005
GORD (RO)	IBD (RO)	0.02	0.075	7.8E-01	-0.04	0.005
IBS (RO)	PUD (RO)	0.25	0.117	3.4E-02	-0.03	0.005
IBS (RO)	GORD (RO)	0.47	0.075	3.4E-10	-0.08	0.005
IBS (RO)	IBD (RO)	-0.08	0.101	4.4E-01	-0.01	0.005
IBD (RO)	PUD (RO)	-0.12	0.120	3.4E-01	-0.02	0.005
IBD (RO)	GORD (RO)	0.02	0.075	7.8E-01	-0.04	0.005
IBD (RO)	IBS (RO)	-0.08	0.101	4.4E-01	-0.01	0.005

[*] In LD score regression the sample overlap is partitioned into the gcov_int (the genetic covariance intercept) for which the expected value is phenotypic correlation * proportion of shred individuals between the two GWAS datasets contributing to the LDSC genetic correlation analysis.

[†] RO: remove individuals with more than one of PUD, GORD, IBS and IBD disorders.

Supplementary Table 8. SNP-based heritability estimates and other parameters from LD score regression[14] for self-report, primary care and hospital admission subgroup phenotypes for each of PUD, GORD and IBS. (see **Supplementary Fig. 10c**)

Digestion Phenotypes	λ_{GC}*	Intercept (s.e.)	h2_observed (s.e.)	Population lifetime risk†	h2_liability (s.e.)
PUD	1.10	1.01 (0.007)	0.011 (0.001)	0.037	0.058 (0.007)
PUD Hospital Admission	1.05	1.01 (0.006)	0.003 (0.001)	0.016	0.052 (0.017)
PUD Self Report	1.05	1.01 (0.007)	0.004 (0.001)	0.008	0.068 (0.020)
PUD Primary Care	1.05	1.01 (0.006)	0.003 (0.001)	0.007	0.028 (0.010)
GORD	1.25	1.02 (0.008)	0.029 (0.002)	0.120	0.077 (0.004)
GORD Hospital Admission	1.10	1.00 (0.007)	0.011 (0.001)	0.054	0.065 (0.008)
GORD Self Report	1.05	1.02 (0.006)	0.004 (0.001)	0.032	0.038 (0.011)
GORD Primary Care	1.15	1.01 (0.007)	0.015 (0.001)	0.018	0.063 (0.006)
IBS	1.15	1.00 (0.007)	0.015 (0.001)	0.063	0.057 (0.005)
IBS Hospital Admission	1.05	1.00 (0.007)	0.005 (0.001)	0.009	0.049 (0.012)
IBS Self Report	1.05	1.01 (0.006)	0.005 (0.001)	0.016	0.035 (0.008)
IBS Primary Care	1.05	1.00 (0.007)	0.004 (0.001)	0.029	0.052 (0.015)

* The calculation for λ_{GC} is from LD score regression.

† We used the proportion of sample that are cases as the estimates of population lifetime.

Supplementary Table 9. Genetic correlation among self-report, primary care and hospital admission subgroup phenotypes for each of PUD, GORD and IBS from bivariate LD score regression analysis[15]. (see **Supplementary Fig. 10d**)

Trait1	Trait2	rg	se	p	gcov_int*	gcov_int_se
PUD Hospital Admission	PUD Primary Care	0.94	0.299	1.6E-03	0.01	0.005
PUD Hospital Admission	PUD Self Report	0.81	0.312	9.7E-03	0.02	0.005
PUD Hospital Admission	PUD	1.06	0.111	2.2E-21	0.66	0.005
PUD Primary Care	PUD Hospital Admission	0.94	0.299	1.6E-03	0.01	0.005
PUD Primary Care	PUD Self Report	0.49	0.230	3.3E-02	0.02	0.005
PUD Primary Care	PUD	0.89	0.099	2.9E-19	0.44	0.005
PUD Self Report	PUD Hospital Admission	0.81	0.312	9.7E-03	0.02	0.005
PUD Self Report	PUD Primary Care	0.49	0.230	3.3E-02	0.02	0.005
PUD Self Report	PUD	0.82	0.104	2.8E-15	0.48	0.005
PUD	PUD Hospital Admission	1.06	0.111	2.2E-21	0.66	0.005
PUD	PUD Primary Care	0.89	0.099	2.9E-19	0.44	0.005
PUD	PUD Self Report	0.82	0.104	2.8E-15	0.48	0.005
GORD Hospital Admission	GORD Primary Care	0.80	0.146	4.1E-08	0.03	0.005
GORD Hospital Admission	GORD Self Report	0.78	0.079	1.8E-23	0.05	0.005
GORD Hospital Admission	GORD	0.97	0.020	0.0E+00	0.68	0.006
GORD Primary Care	GORD Hospital Admission	0.80	0.146	4.1E-08	0.03	0.005
GORD Primary Care	GORD Self Report	0.62	0.161	1.0E-04	0.03	0.005
GORD Primary Care	GORD	0.86	0.105	3.4E-16	0.40	0.005
GORD Self Report	GORD Hospital Admission	0.78	0.079	1.8E-23	0.05	0.005
GORD Self Report	GORD Primary Care	0.62	0.161	1.0E-04	0.03	0.005
GORD Self Report	GORD	0.89	0.037	7.9E-129	0.53	0.006
GORD	GORD Hospital Admission	0.97	0.020	0.0E+00	0.68	0.006
GORD	GORD Primary Care	0.86	0.105	3.4E-16	0.40	0.005
GORD	GORD Self Report	0.89	0.037	7.9E-129	0.53	0.006
IBS Hospital Admission	IBS Primary Care	0.85	0.212	5.8E-05	0.01	0.005
IBS Hospital Admission	IBS Self Report	0.71	0.210	7.0E-04	0.02	0.005
IBS Hospital Admission	IBS	1.01	0.119	1.8E-17	0.35	0.005
IBS Primary Care	IBS Hospital Admission	0.85	0.212	5.8E-05	0.01	0.005
IBS Primary Care	IBS Self Report	0.63	0.158	5.8E-05	0.02	0.005
IBS Primary Care	IBS	0.94	0.052	1.2E-74	0.67	0.005
IBS Self Report	IBS Hospital Admission	0.71	0.210	7.0E-04	0.02	0.005
IBS Self Report	IBS Primary Care	0.63	0.158	5.8E-05	0.02	0.005
IBS Self Report	IBS	0.79	0.068	1.1E-31	0.50	0.006
IBS	IBS Hospital Admission	1.01	0.119	1.8E-17	0.35	0.005
IBS	IBS Primary Care	0.94	0.052	1.2E-74	0.67	0.005

IBS	IBS Self Report	0.79	0.068	1.1E-31	0.50	0.006

[*] In LD score regression the sample overlap is partitioned into the gcov_int (the genetic covariance intercept) for which the expected value is phenotypic correlation * proportion of shred individuals between the two GWAS datasets contributing to the LDSC genetic correlation analysis.

Supplementary Table 10. Genetic correlation estimates between each of the five digestion phenotypes and 9 psychiatric and neurological traits from bivariate LD score regression analysis[15]. (see **Fig. 4b**)

Trait1	Trait2	rg	se	p	h2 (obs)	h2 se (obs)	h2 int	h2 int se	gcov int*	gcov int se
PUD	ADHD†	0.48	0.057	9.1E-17	0.24	0.016	1.03	0.010	0.01	0.006
	Schizophrenia	0.13	0.040	1.0E-03	0.42	0.015	1.05	0.012	0.00	0.006
	Anxiety	0.26	0.179	1.5E-01	0.06	0.026	1.00	0.007	0.00	0.005
	PTSD	0.30	0.164	7.2E-02	0.05	0.022	0.99	0.007	0.00	0.005
	Bipolar disorder	0.01	0.046	8.0E-01	0.34	0.015	1.02	0.008	0.00	0.005
	Autism spectrum disorder	0.00	0.066	9.7E-01	0.20	0.015	1.00	0.008	0.00	0.006
	Alzheimer's disease	0.08	0.093	4.0E-01	0.01	0.005	1.06	0.052	0.00	0.005
	Parkinson's disease	-0.03	0.062	5.8E-01	0.02	0.002	0.98	0.008	0.00	0.005
	Major depression 2018	0.37	0.045	2.1E-16	0.06	0.003	1.01	0.010	0.01	0.006
GORD	ADHD	0.49	0.036	3.1E-42	0.24	0.016	1.03	0.010	0.00	0.006
	Schizophrenia	0.03	0.027	2.4E-01	0.42	0.015	1.05	0.012	0.01	0.006
	Anxiety	0.30	0.127	1.7E-02	0.06	0.026	1.00	0.007	0.01	0.005
	PTSD	0.23	0.119	4.8E-02	0.05	0.022	0.99	0.007	0.00	0.005
	Bipolar disorder	-0.04	0.034	2.5E-01	0.34	0.015	1.02	0.008	0.00	0.006
	Autism spectrum disorder	-0.01	0.043	8.6E-01	0.20	0.015	1.00	0.008	0.01	0.006
	Alzheimer's disease	0.01	0.059	9.0E-01	0.01	0.005	1.06	0.052	0.01	0.006
	Parkinson's disease	-0.07	0.041	1.0E-01	0.02	0.002	0.98	0.008	0.00	0.005
	Major depression 2018	0.37	0.027	3.3E-41	0.06	0.003	1.01	0.010	0.01	0.006
PG+M	ADHD	0.56	0.032	5.0E-66	0.24	0.016	1.03	0.010	0.01	0.007
	Schizophrenia	0.04	0.024	1.1E-01	0.42	0.015	1.05	0.012	0.01	0.007
	Anxiety	0.37	0.132	5.5E-03	0.06	0.026	1.00	0.007	0.01	0.005
	PTSD	0.30	0.107	4.3E-03	0.05	0.022	0.99	0.007	0.00	0.005
	Bipolar disorder	-0.02	0.030	5.2E-01	0.34	0.015	1.02	0.008	0.00	0.006
	Autism spectrum disorder	0.06	0.039	1.4E-01	0.20	0.015	1.00	0.008	0.01	0.006
	Alzheimer's disease	-0.03	0.050	6.0E-01	0.01	0.005	1.06	0.052	0.01	0.006
	Parkinson's disease	-0.06	0.037	1.2E-01	0.02	0.002	0.98	0.008	0.00	0.005
	Major depression 2018	0.43	0.024	1.4E-74	0.06	0.003	1.01	0.010	0.01	0.006

IBS	ADHD	0.30	0.057	1.8E-07	0.24	0.016	1.03	0.010	0.00	0.006
	Schizophrenia	0.15	0.036	3.2E-05	0.42	0.015	1.05	0.012	0.00	0.007
	Anxiety	0.30	0.152	4.9E-02	0.06	0.026	1.00	0.007	0.00	0.005
	PTSD	0.34	0.182	6.2E-02	0.05	0.022	0.99	0.007	0.00	0.005
	Bipolar disorder	0.07	0.048	1.4E-01	0.34	0.015	1.02	0.008	0.01	0.006
	Autism spectrum disorder	0.21	0.061	7.0E-04	0.20	0.015	1.00	0.008	-0.01	0.005
	Alzheimer’s disease	0.05	0.085	5.7E-01	0.01	0.005	1.06	0.052	0.01	0.005
	Parkinson's disease	0.09	0.057	1.3E-01	0.02	0.002	0.98	0.008	0.00	0.005
	Major depression 2018	0.49	0.042	1.1E-31	0.06	0.003	1.01	0.010	0.01	0.006
IBD	ADHD	0.00	0.059	9.6E-01	0.24	0.016	1.03	0.010	0.01	0.006
	Schizophrenia	0.04	0.040	3.7E-01	0.42	0.015	1.05	0.012	0.00	0.006
	Anxiety	0.23	0.148	1.2E-01	0.06	0.026	1.00	0.007	0.00	0.005
	PTSD	0.20	0.161	2.1E-01	0.05	0.022	0.99	0.007	0.00	0.005
	Bipolar disorder	0.02	0.053	7.6E-01	0.34	0.015	1.02	0.008	0.00	0.006
	Autism spectrum disorder	-0.05	0.059	3.8E-01	0.20	0.015	1.00	0.008	0.01	0.005
	Alzheimer’s disease	-0.05	0.092	5.9E-01	0.01	0.005	1.06	0.052	0.00	0.005
	Parkinson's disease	-0.07	0.065	2.5E-01	0.02	0.002	0.98	0.008	0.00	0.005
	Major depression 2018	0.15	0.043	3.0E-04	0.06	0.003	1.01	0.010	0.00	0.005

[*] In LD score regression the sample overlap is partitioned into the gcov_int (the genetic covariance intercept) for which the expected value is phenotypic correlation * proportion of shred individuals between the two GWAS datasets contributing to the LDSC genetic correlation analysis.

[†] Cells highlighted with yellow represent that genetic correlation are still significant after Bonferroni correction.

Supplementary Table 11. Genetic correlation estimates between each of the five sensitivity analyses phenotypes and 9 psychiatric and neurologic traits from bivariate LD score regression analysis[15]. (see **Supplementary Fig. 9c**)

Trait1*	Trait2	rg	se	p	gcov int†	gcov int se
PUD (RO)	ADHD	0.40	0.077	1.8E-07	0.01	0.006
	Schizophrenia	0.06	0.050	2.0E-01	0.00	0.006
	Anxiety	0.34	0.228	1.4E-01	0.00	0.005
	PTSD	0.36	0.223	1.1E-01	0.00	0.005
	Bipolar disorder	-0.05	0.061	4.0E-01	0.00	0.005
	Autism spectrum disorder	0.03	0.073	7.1E-01	0.00	0.005
	Alzheimer’s disease	-0.02	0.112	8.6E-01	0.01	0.005
	Parkinson's disease	-0.02	0.074	7.6E-01	0.01	0.004
	Major depression 2018	0.34	0.059	1.2E-08	0.00	0.005
GORD (RO)	ADHD	0.47	0.042	3.1E-29	0.00	0.006
	Schizophrenia	-0.01	0.029	6.9E-01	0.01	0.006
	Anxiety	0.27	0.133	3.9E-02	0.01	0.005
	PTSD	0.12	0.123	3.1E-01	0.00	0.005
	Bipolar disorder	-0.08	0.037	4.0E-02	0.00	0.005
	Autism spectrum disorder	0.00	0.048	9.3E-01	0.01	0.006
	Alzheimer’s disease	-0.05	0.064	4.1E-01	0.01	0.006
	Parkinson's disease	-0.08	0.044	6.7E-02	0.00	0.005
	Major depression 2018	0.33	0.031	1.4E-27	0.01	0.005
IBS (RO)	ADHD	0.20	0.071	4.3E-03	0.00	0.006
	Schizophrenia	0.16	0.048	1.0E-03	-0.01	0.006
	Anxiety	0.31	0.193	1.1E-01	0.00	0.005
	PTSD	0.14	0.208	5.1E-01	0.01	0.005
	Bipolar disorder	0.05	0.060	3.9E-01	0.01	0.005
	Autism spectrum disorder	0.33	0.074	6.7E-06	-0.01	0.005
	Alzheimer’s disease	-0.04	0.119	7.2E-01	0.01	0.005
	Parkinson's disease	0.14	0.073	5.9E-02	0.00	0.005
	Major depression 2018	0.49	0.057	6.5E-18	0.00	0.006
IBD (RO)	ADHD	-0.09	0.069	1.8E-01	0.01	0.006
	Schizophrenia	0.02	0.043	6.8E-01	0.00	0.006
	Anxiety	0.25	0.168	1.4E-01	0.00	0.005

	PTSD	0.10	0.186	6.0E-01	0.00	0.005
	Bipolar disorder	-0.01	0.059	9.1E-01	0.00	0.005
	Autism spectrum disorder	-0.06	0.065	3.7E-01	0.00	0.005
	Alzheimer’s disease	-0.06	0.110	5.8E-01	0.00	0.005
	Parkinson's disease	-0.08	0.074	2.6E-01	0.00	0.005
	Major depression 2018	0.11	0.050	3.4E-02	0.00	0.006

[*] RO: remove individuals with more than one of PUD, GORD, IBS and IBD disorders.

[†] In LD score regression the sample overlap is partitioned into the gcov_int (the genetic covariance intercept) for which the expected value is phenotypic correlation * proportion of shred individuals between the two GWAS datasets contributing to the LDSC genetic correlation analysis.

Supplementary Table 12. Results of partitioning SNP-based heritability analysis[16] for GORD, PG+M, IBS and IBD by functional annotation based on the variants within each category after Bonferroni correction. (see **Supplementary Fig. 11**)

Trait	Category	Prop._SNPs	Prop. h2	Prop. h2 s.e.	Enrichment	Enrichment s.e.	Enrichment p
GORD	Conserved	0.03	0.39	0.067	14.90	2.553	1.2E-07
	Conserved (extend 500)	0.33	0.58	0.063	1.75	0.190	1.2E-04
	H3K4me1 (extend 500)	0.61	0.93	0.061	1.53	0.100	2.1E-06
PG+M	Conserved	0.03	0.38	0.045	14.60	1.722	1.6E-13
	Conserved (extend 500)	0.33	0.61	0.048	1.85	0.145	2.2E-08
	DHS (extend 500)	0.50	0.85	0.067	1.71	0.134	8.0E-07
	Fetal DHS	0.08	0.34	0.067	4.05	0.792	1.4E-04
	Fetal DHS (extend 500)	0.29	0.59	0.064	2.06	0.223	5.5E-06
	H3K27ac	0.39	0.51	0.031	1.31	0.078	1.6E-04
	H3K27ac PGC2 (extend 500)	0.34	0.52	0.045	1.53	0.134	1.0E-04
	H3K4me1 (extend 500)	0.61	0.91	0.042	1.49	0.068	1.0E-10
	H3K4me3	0.13	0.34	0.048	2.54	0.361	2.0E-05
	H3K9ac	0.13	0.37	0.051	2.90	0.404	2.6E-06
	H3K9ac (extend 500)	0.23	0.48	0.051	2.07	0.223	4.8E-06
	Intron	0.39	0.49	0.023	1.25	0.059	1.9E-05
	Intron (extend 500)	0.40	0.47	0.020	1.19	0.050	1.6E-04
	Repressed (extend 500)	0.72	0.61	0.028	0.85	0.039	1.7E-04
	Super Enhancer	0.17	0.24	0.018	1.45	0.108	5.4E-05
	Super Enhancer (extend 500)	0.17	0.25	0.019	1.48	0.109	2.2E-05
IBS	Conserved	0.03	0.51	0.098	19.61	3.761	1.2E-06
	Conserved (extend 500)	0.33	0.82	0.110	2.47	0.331	8.8E-06
IBD	Enhancer (extend 500)	0.15	0.71	0.137	4.64	0.888	5.7E-05
	H3K27ac	0.39	0.95	0.100	2.43	0.256	5.9E-10
	H3K27ac (extend 500)	0.42	1.00	0.108	2.37	0.255	7.5E-09
	H3K4me1 (extend 500)	0.61	1.03	0.095	1.69	0.156	4.0E-05
	H3K4me3	0.13	0.76	0.178	5.71	1.334	1.4E-04
	Repressed (extend 500)	0.72	0.24	0.085	0.33	0.118	1.1E-10
	Super Enhancer	0.17	0.54	0.068	3.20	0.404	9.0E-09
	Super Enhancer (extend 500)	0.17	0.62	0.062	3.64	0.362	7.7E-14

Supplementary Table 13. Results of partitioning SNP-based heritability analysis for PUD, GORD, PG$_{+}$M, IBS, IBD and PG$_{+}$M (sensitivity analysis after conditioning on other traits) by cell group functional annotations[16]. (see **Fig. 5a**)

Trait	Category	Prop. SNPs	Prop. h2	Prop. h2 s.e.	Enrichment	Enrichment s.e.	Enrichment p
PUD	Adrenal Pancreas	0.09	0.36	0.104	3.83	1.108	1.2E-02
	Central Nervous System	0.15	0.47	0.109	3.16	0.734	3.5E-03
	Gastrointestinal	0.17	0.54	0.129	3.24	0.767	4.2E-03
	Immune	0.23	0.44	0.122	1.90	0.521	8.7E-02
	Liver	0.07	0.25	0.084	3.53	1.160	2.9E-02
GORD	Adrenal Pancreas	0.09	0.26	0.047	2.81	0.504	5.2E-04
	Central Nervous System*	0.15	0.38	0.046	2.52	0.310	2.1E-06
	Gastrointestinal	0.17	0.31	0.053	1.87	0.315	7.7E-03
	Immune	0.23	0.35	0.056	1.52	0.241	3.4E-02
	Liver	0.07	0.15	0.034	2.05	0.470	2.8E-02
PG$_{+}$M	Adrenal Pancreas	0.09	0.27	0.037	2.91	0.394	2.1E-06
	Central Nervous System	0.15	0.41	0.034	2.75	0.230	2.2E-13
	Gastrointestinal	0.17	0.30	0.040	1.79	0.237	1.2E-03
	Immune	0.23	0.39	0.040	1.65	0.171	1.9E-04
	Liver	0.07	0.16	0.027	2.22	0.374	1.2E-03
IBS	Adrenal Pancreas	0.09	0.31	0.085	3.35	0.908	8.2E-03
	Central Nervous System	0.15	0.55	0.089	3.70	0.596	7.8E-06
	Gastrointestinal	0.17	0.31	0.106	1.84	0.631	1.8E-01
	Immune	0.23	0.39	0.114	1.67	0.487	1.6E-01
	Liver	0.07	0.20	0.068	2.82	0.936	5.0E-02
IBD	Adrenal Pancreas	0.09	0.42	0.105	4.51	1.123	9.9E-04
	Central Nervous System	0.15	0.34	0.114	2.28	0.767	8.9E-02
	Gastrointestinal	0.17	0.73	0.140	4.37	0.833	3.3E-05
	Immune	0.23	1.00	0.139	4.30	0.596	5.7E-08
	Liver	0.07	0.24	0.078	3.26	1.078	3.6E-02
PG$_{+}$M (Conditional on EA, BMI and smoking traits)	Adrenal Pancreas	0.09	0.28	0.039	3.04	0.42	2.4E-06
	Central Nervous System	0.15	0.40	0.037	2.66	0.25	7.3E-11
	Gastrointestinal	0.17	0.31	0.046	1.87	0.28	2.2E-03
	Immune	0.23	0.39	0.051	1.69	0.22	1.7E-03
	Liver	0.07	0.16	0.032	2.18	0.44	8.2E-03

* Cells highlighted with yellow represent that tissue are still significant after Bonferroni correction.

Supplementary Table 14. Statistically significant results of partitioning SNP-based heritability of PG$_{+}$M and IBD to 205 tissues/cell types after Bonferroni correction and PG$_{+}$M (sensitivity analyses for 3 tissues after conditioning on other traits) using LDSC cell type specific expressed genes analysis[17]. (see **Fig. 5b**)

Trait	Name	Tissue category for figures	Coefficient	Coefficient s.e.	Coefficient P value	-log10(P)
IBD	Leukocytes	Blood/Immune	4.1E-09	1.0E-09	2.4E-05	4.62
	Blood Cells		3.8E-09	9.7E-10	3.8E-05	4.42
PG$_{+}$M	Brain Hippocampus	CNS	2.5E-09	6.4E-10	5.8E-05	4.24
	Brain Frontal Cortex (BA9)		2.3E-09	6.2E-10	7.2E-05	4.14
	Brain Anterior cingulate cortex (BA24)		2.4E-09	6.4E-10	1.0E-04	3.98
PG$_{+}$M (Conditional on EA, BMI and smoking traits)	Brain Anterior cingulate cortex (BA24)	CNS	1.7E-09	5.9E-10	0.002	2.70
	Brain Frontal Cortex (BA9)		1.6E-09	5.7E-10	0.002	2.70
	Brain Hippocampus		1.7E-09	5.7E-10	0.002	2.70

Supplementary Table 15. Results of partitioning SNP-based heritability of five digestion phenotypes and PG$_{+}$M (sensitivity analyses after conditioning on other traits) to final-scale GTEx brain expression data using LDSC cell type specific expressed genes analysis[17]. (see **Fig. 5c**)

Trait	Name	Coefficient	Coefficient s.e.	Coefficient P value
GORD	Cortex*	2.1E-09	6.1E-10	3.7E-04
	Frontal Cortex (BA9)	1.4E-09	5.0E-10	2.2E-03
	Anterior cingulate cortex (BA24)	9.1E-10	5.0E-10	3.3E-02
	Cerebellum	1.1E-09	6.5E-10	4.6E-02
	Cerebellar Hemisphere	7.3E-10	6.4E-10	1.3E-01
	Amygdala	-2.7E-11	5.8E-10	5.2E-01
	Hippocampus	-1.2E-10	5.1E-10	5.9E-01
	Caudate (basal ganglia)	-1.5E-10	5.7E-10	6.0E-01
	Putamen (basal ganglia)	-3.5E-10	5.6E-10	7.3E-01
	Substantia nigra	-4.9E-10	5.8E-10	8.0E-01
	Hypothalamus	-4.5E-10	5.2E-10	8.1E-01
	Nucleus accumbens (basal ganglia)	-5.0E-10	5.1E-10	8.4E-01
	Spinal cord (cervical c-1)	-1.1E-09	5.8E-10	9.7E-01
PG$_{+}$M	Cortex	3.1E-09	7.5E-10	2.3E-05
	Frontal Cortex (BA9)	2.2E-09	6.3E-10	2.4E-04
	Anterior cingulate cortex (BA24)	1.4E-09	6.4E-10	1.5E-02
	Cerebellum	1.5E-09	7.9E-10	2.5E-02
	Cerebellar Hemisphere	1.3E-09	7.6E-10	4.7E-02
	Nucleus accumbens (basal ganglia)	5.5E-10	6.6E-10	2.0E-01
	Caudate (basal ganglia)	-8.9E-11	7.2E-10	5.5E-01
	Spinal cord (cervical c-1)	-2.7E-10	7.6E-10	6.4E-01
	Amygdala	-3.8E-10	7.3E-10	7.0E-01
	Hippocampus	-5.6E-10	6.4E-10	8.1E-01
	Hypothalamus	-5.8E-10	6.3E-10	8.2E-01
	Substantia nigra	-1.0E-09	6.8E-10	9.3E-01
	Putamen (basal ganglia)	-1.4E-09	6.7E-10	9.8E-01
IBS	Cerebellum	7.4E-10	5.3E-10	8.1E-02
	Frontal Cortex (BA9)	5.9E-10	4.5E-10	9.7E-02
	Cortex	5.2E-10	5.1E-10	1.5E-01
	Cerebellar Hemisphere	2.9E-10	4.7E-10	2.6E-01
	Putamen (basal ganglia)	2.4E-10	4.8E-10	3.1E-01
	Hippocampus	1.4E-10	4.6E-10	3.8E-01
	Anterior cingulate cortex (BA24)	-8.3E-12	4.5E-10	5.1E-01
	Caudate (basal ganglia)	-5.6E-11	4.4E-10	5.5E-01
	Amygdala	-2.8E-10	4.3E-10	7.4E-01
	Spinal cord (cervical c-1)	-3.4E-10	4.2E-10	7.9E-01
	Nucleus accumbens (basal ganglia)	-3.6E-10	4.3E-10	8.0E-01

	Substantia nigra	-6.5E-10	4.3E-10	9.3E-01
	Hypothalamus	-1.0E-09	4.5E-10	9.9E-01
PG$_{+}$M (Conditional on EA, BMI and smoking traits)	Cortex	2.6E-09	6.7E-10	4.2E-05
	Frontal Cortex (BA9)	1.9E-09	6.0E-10	7.1E-04
	Cerebellum	1.9E-09	7.0E-10	4.0E-03
	Anterior cingulate cortex (BA24)	1.3E-09	5.8E-10	1.5E-02
	Cerebellar Hemisphere	9.2E-10	6.7E-10	8.5E-02
	Nucleus accumbens (basal ganglia)	-1.9E-13	5.7E-10	5.0E-01
	Amygdala	-1.9E-10	6.4E-10	6.2E-01
	Spinal cord (cervical c-1)	-3.5E-10	6.9E-10	6.9E-01
	Hypothalamus	-3.6E-10	6.6E-10	7.1E-01
	Caudate (basal ganglia)	-5.6E-10	6.1E-10	8.2E-01
	Hippocampus	-5.9E-10	6.2E-10	8.3E-01
	Substantia nigra	-7.6E-10	6.0E-10	9.0E-01
	Putamen (basal ganglia)	-1.3E-09	5.6E-10	9.9E-01

* Cells highlighted with yellow represent that tissue are still significant after Bonferroni correction.

Supplementary Table 16. Statistically significant results for the 3 digestion traits using GTEx and eQTLGen data and SMR analysis[18].

eQTL Data	Trait	Tissue	Probe ID*	Probe Chr.	Probe base pair position	Gene	Top SNP	A1/ A2	Freq	P_{GWAS}	P_{eQTL}	b_{SMR}	P_{SMR}	P_{HEIDI}	HEIDI pass
GTEx	PUD	Stomach	ENSG00000167653.4	8	143757934	*PSCA*	rs2976388	A/G	0.42	1.8E-14	8.8E-41	-0.12	3.0E-11	0.59	Yes
			ENSG00000176920.10	19	49204217	*FUT2*	rs601338	A/G	0.51	2.4E-15	2.8E-20	-0.31	1.9E-09	0.89	Yes
	IBD	Colon Transverse	ENSG00000257582.1	10	101288520	*RP11-129J12.2*	rs1548962	C/G	0.51	9.7E-11	7.0E-22	0.20	7.9E-08	0.02	Yes
eQTL Gen	PG+M	Blood	ENSG00000139531	12	56395694	*SUOX*	rs1873914	C/G	0.42	4.8E-08	0.0E+00	0.08	6.2E-08	0.74	Yes
	IBD		ENSG00000224203	1	161507154	*RPS23P10*	rs9427403	C/A	0.18	3.1E-08	0.0E+00	0.28	4.1E-08	0.06	Yes

* All listed results are significant after Bonferroni correction at P < 0.05/131,295.

Supplementary Table 17. Statistically significant results for PUD using blood mQTL data from McRae *et al.*[19] and SMR analysis[18].

mQTL Data	Trait	Probe ID*	Probe Chr.	Probe base pair position	Top SNP	A1/ A2	Freq	P_{GWAS}	P_{eQTL}	b_{SMR}	P_{SMR}	P_{HEIDI}	HEIDI pass
Blood mQTL data from McRae *et al.*[19]	PUD	cg08900798	11	1029938	rs59257210	T/C	0.12	9.6E-10	8.6E-42	-0.16	2.5E-08	0.06	Yes
	PUD	cg01656853	19	49199172	rs633372	G/A	0.47	1.2E-12	7.7E-56	0.16	9.6E-11	0.07	Yes
	PUD	cg04660111	19	49199234	rs507855	A/G	0.47	6.1E-12	1.5E-57	0.16	2.6E-10	0.05	Yes
	PUD	cg08873673	19	49199217	rs2548459	T/C	0.47	1.2E-12	1.8E-40	0.19	3.7E-10	0.19	Yes
	PUD	cg11773468	19	49216319	rs12978752	A/C	0.45	8.4E-11	9.5E-52	-0.15	2.4E-09	0.04	Yes
	PUD	cg15673069	19	49232542	rs838144	T/C	0.50	1.8E-10	1.0E-40	-0.17	8.6E-09	0.01	Yes
	PUD	cg21653641	19	49222892	rs35866622	C/T	0.50	7.4E-13	1.9E-16	0.31	6.4E-08	0.04	Yes
	PUD	cg01652021	8	143851375	rs4736300	A/G	0.31	2.1E-07	4.5E-167	0.07	3.3E-07	0.08	Yes
	PUD	cg09147065	8	143815906	rs2257796	C/T	0.48	1.0E-10	2.5E-16	-0.27	3.9E-07	0.01	Yes
	PUD	cg09552631	8	143763326	rs2585183	G/C	0.42	6.9E-14	4.3E-21	-0.28	4.5E-09	0.19	Yes
	PUD	cg14304364	8	143807962	rs2164308	T/C	0.49	1.4E-10	4.4E-20	-0.24	1.4E-07	0.08	Yes
	PUD	cg24023258	8	143781297	rs2585175	G/C	0.51	3.3E-10	1.5E-73	-0.12	2.9E-09	0.03	Yes
	PUD	cg11879188	9	136149908	rs582094	T/A	0.32	3.2E-09	2.1E-183	-0.08	6.6E-09	0.59	Yes
	PUD	cg13506600	9	136150361	rs687621	G/A	0.32	1.3E-09	1.5E-79	-0.11	7.5E-09	0.26	Yes
	PUD	cg13531387	9	136078657	rs2769071	G/A	0.32	3.0E-09	1.7E-38	0.15	6.9E-08	0.30	Yes
	PUD	cg14271713	9	136153846	rs529565	C/T	0.32	2.7E-09	4.2E-45	0.14	4.2E-08	0.65	Yes
	PUD	cg21160290	9	136149941	rs554833	T/C	0.32	3.6E-09	0.0E+00	-0.05	4.4E-09	0.48	Yes
	PUD	cg22535403	9	136150032	rs554833	T/C	0.32	3.6E-09	0.0E+00	-0.05	4.5E-09	0.37	Yes
	PUD	cg24267699	9	136151359	rs582094	T/A	0.32	3.2E-09	1.5E-162	-0.08	7.2E-09	0.43	Yes

* All listed results are significant after Bonferroni correction at P < 0.05/92,715.

Supplementary Table 18. Results of gene sets enrichment analysis at FDR < 5% for each of $PG_{+}M$ and IBD using MAGMA[20].

Trait	Gene sets name	No. of genes[*]	beta[†]	SE[‡]	P[§]	-log10(P)
$PG_{+}M$	GO POSTSYNAPSE	582	0.19	0.040	9.3E-07	6.03
	GO POSTSYNAPTIC MEMBRANE	302	0.24	0.057	1.3E-05	4.89
	GO SYNAPSE PART	891	0.13	0.032	1.6E-05	4.78
	GO SYNAPSE	1116	0.12	0.029	1.8E-05	4.75
	GO AXON PART	358	0.19	0.052	1.0E-04	4.00
	GO SYNAPTIC MEMBRANE	404	0.18	0.049	1.0E-04	4.00
	GO NEURON PROJECTION	1233	0.10	0.028	1.0E-04	3.98
	GO DENDRITIC TREE	558	0.15	0.041	1.1E-04	3.97
	GO NEURON PART	1629	0.09	0.024	1.4E-04	3.85
	GO GLUTAMATERGIC SYNAPSE	331	0.18	0.052	2.6E-04	3.58
	GO SOMATODENDRITIC COMPARTMENT	778	0.11	0.035	4.4E-04	3.36
IBD	GO MHC CLASS II PROTEIN COMPLEX	14	1.27	0.290	6.4E-06	5.19
	GO MHC PROTEIN COMPLEX	22	0.85	0.203	1.5E-05	4.82
	GO SIDE OF MEMBRANE	480	0.17	0.043	5.8E-05	4.24
	GO LUMENAL SIDE OF MEMBRANE	29	0.68	0.193	2.0E-04	3.70
	GO HOST CELL CYTOPLASM	6	1.52	0.433	2.3E-04	3.64
	GO ER TO GOLGI TRANSPORT VESICLE MEMBRANE	56	0.45	0.130	2.7E-04	3.58

[*] The number of genes in the gene-based summary statistics that are also in the gene sets.
[†] The regression coefficient of the gene set.
[‡] The standard error of the regression coefficient.
[§] The competitive gene set P value.

Supplementary Table 19. Number of cases and controls for the 4 digestion phenotypes and 8 depression phenotypes and 2 by 2 contingency table for number of cases and controls of each of the 32 digestion-depression phenotype pairs*.

		GORD		PUD		IBS		IBD	
GPpsy		Case	Control	Case	Control	Case	Control	Case	Control
		40514	305450	12194	333770	22138	323826	5263	340701
Case	118966	18117	100849	5076	113890	12195	106771	1897	117069
Control	226998	22397	204601	7118	219880	9943	217055	3366	223632
Psypsy		Case	Control	Case	Control	Case	Control	Case	Control
		40646	306156	12227	334575	22218	324584	5268	341534
Case	40262	6571	33691	2056	38206	4381	35881	689	39573
Control	306540	34075	272465	10171	296369	17837	288703	4579	301961
DepAll		Case	Control	Case	Control	Case	Control	Case	Control
		9458	74053	2672	80839	5096	78415	1167	82344
Case	22414	3272	19142	875	21539	2162	20252	327	22087
Control	61097	6186	54911	1797	59300	2934	58163	840	60257
SelfRepDep		Case	Control	Case	Control	Case	Control	Case	Control
		36975	223316	11195	249096	19868	240423	4983	255308
Case	19914	3624	16290	1052	18862	2543	17371	333	19581
Control	240377	33351	207026	10143	230234	17325	223052	4650	235727
ICD10Dep		Case	Control	Case	Control	Case	Control	Case	Control
		38095	232382	11536	258941	20032	250445	5052	265425
Case	9947	2424	7523	804	9143	1461	8486	249	9698
Control	260530	35671	224859	10732	249798	18571	241959	4803	255727
LifetimeMDD		Case	Control	Case	Control	Case	Control	Case	Control
		6614	60205	1763	65056	3749	63070	865	65954
Case	16945	2252	14693	527	16418	1717	15228	264	16681
Control	49874	4362	45512	1236	48638	2032	47842	601	49273
MDDRecur		Case	Control	Case	Control	Case	Control	Case	Control
		5790	54207	1560	58437	3167	56830	766	59231
Case	10216	1437	8779	330	9886	1144	9072	166	10050
Control	49781	4353	45428	1230	48551	2023	47758	600	49181
GPNoDep		Case	Control	Case	Control	Case	Control	Case	Control
		6150	54652	1786	59016	2905	57897	834	59968
Case	9066	1289	7777	341	8725	813	8253	132	8934
Control	51736	4861	46875	1445	50291	2092	49644	702	51034

* Cells with blue colour represent eight depression phenotypes following definition of Cai *et al.*[21] and corresponding number of cases and controls and cells with orange colour represent four digestion phenotypes and corresponding number of cases and controls. Cells with red colour represent the number of overlapped cases and controls for each of 32 digestion-depression phenotype pairs.

Abbreviation: Seen general practice (GP) for nerves, anxiety, tension or depression (GPpsy); Seen psychiatrist for nerves, anxiety, tension or depression (Psypsy); Probable recurrent major depression or single probable major depression episode (DepAll); Self-reported depression (SelfRepDep); ICD10 defined depression (ICD10Dep); DSM-V clinical guideline defined major depression (LifetimeMDD); Major depression recurrence (MDDRecur); Seen GP for depression but no cardinal symptoms (GPNoDep); Peptic ulcer disease (PUD); Gastro-oesophageal reflux disease (GORD); Irritable bowel syndrome (IBS) and Inflammatory bowel disease (IBD).

Supplementary Table 20. Results of odds ratio and corresponding P value from 2 by 2 contingency table (**Supplementary Table 19**) of each of the 32 digestion-depression phenotype pairs using fmsb R package[22].

Depression phenotypes	Digestion phenotypes	Beta	s.e.	P	OR	CIL*	CIU*
GPpsy	PUD	0.32	0.019	0.0E+00	1.38	1.33	1.43
GPpsy	GORD	0.50	0.011	0.0E+00	1.64	1.61	1.68
GPpsy	IBS	0.91	0.014	0.0E+00	2.49	2.43	2.56
GPpsy	IBD	0.07	0.029	1.1E-02	1.08	1.02	1.14
Psypsy	PUD	0.45	0.025	0.0E+00	1.57	1.49	1.65
Psypsy	GORD	0.44	0.015	0.0E+00	1.56	1.52	1.60
Psypsy	IBS	0.68	0.018	0.0E+00	1.98	1.91	2.05
Psypsy	IBD	0.14	0.041	7.9E-04	1.15	1.06	1.24
DepAll	PUD	0.29	0.042	2.5E-12	1.34	1.23	1.46
DepAll	GORD	0.42	0.023	0.0E+00	1.52	1.45	1.59
DepAll	IBS	0.75	0.029	0.0E+00	2.12	2.00	2.24
DepAll	IBD	0.06	0.066	3.6E-01	1.06	0.93	1.21
SelfRepDep	PUD	0.24	0.033	1.2E-12	1.27	1.19	1.35
SelfRepDep	GORD	0.32	0.019	0.0E+00	1.38	1.33	1.43
SelfRepDep	IBS	0.63	0.023	0.0E+00	1.88	1.80	1.97
SelfRepDep	IBD	-0.15	0.057	9.4E-03	0.86	0.77	0.96
ICD10Dep	PUD	0.72	0.038	0.0E+00	2.05	1.90	2.21
ICD10Dep	GORD	0.71	0.024	0.0E+00	2.03	1.94	2.13
ICD10Dep	IBS	0.81	0.029	0.0E+00	2.24	2.12	2.38
ICD10Dep	IBD	0.31	0.066	1.8E-06	1.37	1.20	1.56
LifetimeMDD	PUD	0.23	0.053	9.3E-06	1.26	1.14	1.40
LifetimeMDD	GORD	0.47	0.028	0.0E+00	1.60	1.51	1.69
LifetimeMDD	IBS	0.98	0.034	0.0E+00	2.65	2.48	2.84
LifetimeMDD	IBD	0.26	0.074	4.5E-04	1.30	1.12	1.50
MDDRecur	PUD	0.28	0.063	1.1E-05	1.32	1.16	1.49
MDDRecur	GORD	0.54	0.033	0.0E+00	1.71	1.60	1.82
MDDRecur	IBS	1.09	0.039	0.0E+00	2.98	2.76	3.21
MDDRecur	IBD	0.30	0.088	5.8E-04	1.35	1.14	1.61
GPNoDep	PUD	0.31	0.061	4.7E-07	1.36	1.21	1.53
GPNoDep	GORD	0.47	0.034	0.0E+00	1.60	1.50	1.71
GPNoDep	IBS	0.85	0.043	0.0E+00	2.34	2.15	2.54
GPNoDep	IBD	0.07	0.096	4.5E-01	1.07	0.89	1.30

* CIL and CIU represent the lower and upper value for 95% confidence interval (CI), respectively.

Abbreviation: Seen general practice (GP) for nerves, anxiety, tension or depression (GPpsy); Seen psychiatrist for nerves, anxiety, tension or depression (Psypsy); Probable recurrent major depression or single probable major depression episode (DepAll); Self-reported depression (SelfRepDep); ICD10 defined depression (ICD10Dep); DSM-V clinical guideline defined major depression (LifetimeMDD); Major depression recurrence (MDDRecur); Seen GP for depression but no cardinal symptoms (GPNoDep); Peptic ulcer disease (PUD); Gastro-oesophageal reflux disease (GORD); Irritable bowel syndrome (IBS) and Inflammatory bowel disease (IBD).

Supplementary Table 21. Results of investigating the causality hypothesis between major depression and five digestion phenotypes using Generalised Summary-data-based Mendelian Randomisation[23]. (GSMR, see **Fig. 6b**)

Trait1	Trait2	Direction Trait1 -> Trait 2*				Direction Trait2 -> Trait 1†				
		b_{xy}‡	se	p	No. of SNPs	b_{xy}‡	se	p	No. of SNPs	Significance threshold
Major depression	PUD	0.18	0.063	3.9E-03	33	0.01§	0.020	8.0E-01	13	5.0E-07
						0.018	0.023	4.4E-01	8#	5.0E-08
Major depression	GORD	0.21	0.038	2.2E-08	32	0.18	0.036	7.3E-07	17	5.0E-07
Major depression	$PG_{+}M$	0.24	0.030	2.5E-15	33	0.23	0.038	2.7E-09	18	5.0E-08
Major depression	IBS	0.39	0.050	6.4E-15	33	0.11	0.033	8.9E-04	12	2.0E-06
Major depression	IBD	0.08	0.097	4.0E-01	33	0.01	0.008	1.1E-01	31	5.0E-08

* The direction represents using trait 1 as exposure to investigate the causality hypothesis on trait 2.

† The direction represents using trait 2 as exposure to investigate the causality hypothesis on trait 1.

‡ The unit represents per standard deviation change in liability to the exposure trait.

§ Yellow highlighted cells indicate use of a relaxed significance threshold for genetic instrument inclusion in the Trait2 -> Trait1 analysis, specified in the significance threshold column.

# Given the unidirectional statistically significant GSMR results between major depression and PUD (i.e., Major depression -> PUD) and relaxed significance threshold for the reverse direction (PUD -> Major depression) to obtain SNPs > 10, we also conducted GSMR analyses using 8 genome-wide significant SNPs for PUD. It remains unidirectional statistically significant for GSMR between major depression and PUD, suggesting that major depression is putatively causal for PUD. This analyses should be revisited when we have more genome-wide significant SNPs for PUD.

Supplementary Table 22. Results of investigating the causality hypothesis between major depression (MD) and $PG_{+}M$ using different Mendelian Randomization (MR) methods.

Exposure	Outcome	Method	No. of SNPs	b[*]	se	P	Egger intercept (se)	Egger intercept P
MD	$PG_{+}M$	Inverse variance weighted	34	0.24	0.03	1.9E-12	-	-
MD	$PG_{+}M$	MR Egger	34	0.10	0.14	4.8E-01	0.005 (0.005)	2.8E-1
MD	$PG_{+}M$	Weighted median	34	0.23	0.05	3.2E-07	-	-
MD	$PG_{+}M$	MR-PRESSO	34	0.24	0.03	4.7E-08[†]	-	-
$PG_{+}M$	MD	Inverse variance weighted	19	0.23	0.05	2.5E-06	-	-
$PG_{+}M$	MD	MR Egger	19	0.01	0.23	9.6E-01	0.008 (0.008)	3.4E-1
$PG_{+}M$	MD	Weighted median	19	0.19	0.06	1.9E-03	-	-
$PG_{+}M$	MD	MR-PRESSO	19	0.23	0.05	1.8E-04[†]	-	-

[*] The beta is the original value from the corresponding MR analysis. The unit represents per standard deviation change in liability to the exposure trait.

[†] No MR-PRESSO (Pleiotropy Residual Sum and Outlier) outliers were detected.

Abbreviation: MR: mendelian randomization; $PG_{+}M$: phenotype for peptic ulcer disease, gastro-oesophageal reflux disease with the combination of taking corresponding medications/treatments.

Supplementary Table 23. Results for the relationship between major depression (MD) and PG$_{+}$M after controlling EA, BMI and smoking-related traits using Generalised Summary-data-based Mendelian Randomisation (GSMR) analyses[23].

Exposure trait	Outcome trait	Conditional trait(s) for exposure trait	b_{xy}*	SE_{xy}	P	No. of SNPs
MD	PG$_{+}$M	EA	0.22	0.03	2.7e-13	32
		BMI	0.27	0.04	3.6e-14	26
		Smoking (Age of Initiation, Cigarettes Per Day, Smoking Cessation and Smoking Initiation)	0.24	0.03	2.7e-15	33
		EA + BMI + Smoking	0.26	0.04	4.4e-13	25
PG$_{+}$M	MD	EA	0.15	0.05	2.5e-03	11
		BMI	0.20	0.04	7.9e-07	16
		Smoking (Age of Initiation, Cigarettes Per Day, Smoking Cessation and Smoking Initiation)	0.23	0.04	2.7e-09	18
		EA + BMI + Smoking	0.19	0.05	4.7e-04	9†

* The unit represents per standard deviation change in liability to the exposure trait.

† GSMR requires at least 10 SNPs as genetic instrument for exposure. After controlling for EA, BMI and smoking-related traits, only 9 SNPs retained, we used these 9 SNPs as genetic instrument rather than relaxing the significance threshold to obtain more SNPs.

Supplementary Table 24. LCV[8] results for the relationship between $PG_{+}M$ (trait 1) and major depression (MD) (trait 2).

LCV output	Value	Meaning
zscore	-1.17	Z score for partial genetic causality. zscore>>0 implies gcp>0
pval.gcpzero.2tailed[*]	0.24	2-tailed p-value for null that gcp=0
gcp.pm	-0.11	genetic causality proportion (gcp)<br>posterior mean gcp (gcp=1: trait 1 -> trait 2; gcp=-1: trait 2-> trait 1)
gcp.pse	0.09	posterior standard error for gcp
rho.est	0.43	estimated genetic correlation
rho.err	0.03	standard error of rho estimate
pval.fullycausal[*]	3.5E-46 for MD -> $PG_{+}M$ and 8.1E-26 for $PG_{+}M$ -> MD	[2 entries], p-values for null that gcp=1 or that gcp=-1, respectively
h2.zscore	34.1 for $PG_{+}M$ and 33.6 for MD	[2 entries], z scores for trait 1 and trait 2 being heritable, respectively

[*] Since pval.gcpzero.2tailed is non-significant and pval.fullycausal is significant in both directions these results provide no evidence for causal directional relationships.

Supplementary Table 25. Genetic correlation estimates between major depression and each of the five GI-DepComRMV phenotypes from bivariate LD score regression analysis[15].

Trait1	Trait2	No. of Case	No. of Control	rg	se	p
Major depression 2018	PUD	16666	439661	0.37	0.045	2.1E-16
	PUD-DepComRMV	9345	279469	0.24	0.050	2.1E-6
	GORD	54854	401473	0.37	0.027	3.3E-41
	GORD-DepComRMV	29090	259724	0.29	0.036	4.1E-16
	PG$_+$M	90175	366152	0.43	0.024	1.4E-74
	PG$_+$M-DepComRMV	48180	240634	0.34	0.029	8.6E-29
	IBS	28518	426803	0.49	0.042	1.1E-31
	IBS-DepComRMV	12033	276269	0.37	0.066	1.6E-8
	IBD	7045	449282	0.15	0.043	0.0003
	IBD-DepComRMV	4272	284542	0.18	0.057	0.0018

Supplementary Table 26. Results of investigating the causality hypothesis between major depression and each of the five GI-DepComRMV phenotypes using Generalised Summary-data-based Mendelian Randomisation[23] (GSMR).

Trait1	Direction Trait1 -> Trait 2 Original Phenotype[*]					Direction Trait1 -> Trait 2 Sensitivity Phenotype[†]				
	Trait2 Original Phenotype	b_{xy}[‡]	se	p	No. of SNPs	Trait2 Sensitivity Phenotype	b_{xy}[‡]	se	p	No. of SNPs
Major depression	PUD	0.18	0.063	3.9E-03	33	PUD-DepComRMV	0.08	0.084	0.366	33
Major depression	GORD	0.21	0.038	2.2E-08	32	GORD-DepComRMV	0.25	0.050	8.3E-7	33
Major depression	$PG_{+}M$	0.24	0.030	2.5E-15	33	$PG_{+}M$-DepComRMV	0.20	0.040	6.1E-7	33
Major depression	IBS	0.39	0.050	6.4E-15	33	IBS-DepComRMV	0.36	0.076	2.3E-6	33
Major depression	IBD	0.08	0.097	4.0E-01	33	IBD-DepComRMV	0.12	0.124	0.342	33

[*] The direction represents using trait 1 as exposure to investigate the causality hypothesis on trait 2 original phenotype.

[†] The direction represents using trait 1 as exposure to investigate the causality hypothesis on trait 2 sensitivity phenotype.

[‡] The unit represents per standard deviation change in liability to the exposure trait.

Supplementary Table 27. Results of investigating the causality hypothesis between major depression and eight PUD sensitivity phenotypes using Generalised Summary-data-based Mendelian Randomisation[23] (GSMR).

Trait1	Direction Trait1 -> Trait 2*						
	Trait2	No. of cases	No. of Controls	b_{xy}†	se	p	No. of SNPs
Major depression	PUD	16666	439661	0.18	0.063	3.9E-03	33
Major depression	PUD-GPpsyRMV	9790	290105	0.09	0.083	0.30	33
Major depression	PUD-PsypsyRMV	13867	390048	0.17	0.070	0.018	33
Major depression	PUD-DepAllRMV	15533	411862	0.20	0.066	0.002	33
Major depression	PUD-SelfRepDepRMV	15235	414915	0.17	0.067	0.010	33
Major depression	PUD-ICD10DepRMV	15566	427451	0.18	0.066	0.008	33
Major depression	PUD-LifetimeMddRMV	15972	418474	0.18	0.066	0.005	33
Major depression	PUD-MddRecurRMV	16227	426918	0.17	0.065	0.008	33
Major depression	PUD-GPNoDepRMV	16196	428425	0.18	0.065	0.008	33

* The direction represents using trait 1 as exposure to investigate the causality hypothesis on trait 2.

† The unit represents per standard deviation change in liability to the exposure trait.

Supplementary Table 28. Polygenic score for major depression* predicts $PG_{+}M$ (peptic ulcer disease (PUD), gastro-oesophageal reflux disease (GORD) in combination with medications/treatments for PUD and GORD) risk using logistic regression model.

Clumping Range	Sample Prevalence (P)	Sample Size (N)	P value of case-control polygenic score difference	AUC	ORD_{10} †	ORD_{10} CI‡
0-5.0E-08	0.198	456327	4.7E-13	0.508	1.08	1.05-1.12
0-1.0E-05			1.5E-45	0.515	1.19	1.15-1.23
0-1.0E-04			2.6E-63	0.517	1.26	1.22-1.30
0-1.0E-03			4.0E-89	0.521	1.30	1.26-1.34
0-1.0E-02			2.2E-118	0.525	1.33	1.29-1.38
0-5.0E-02			3.0E-87	0.521	1.29	1.25-1.34
0-1.0E-01			1.6E-93	0.522	1.29	1.25-1.33
0-5.0E-01			8.6E-87	0.521	1.26	1.22-1.30

* The GWAS summary statistics data for major depression are from Wray *et al.*[24] (European ancestry and UK Biobank participants were excluded).

† The odds ratio of $PG_{+}M$ risk for participants with polygenic score at 10th decile compared with participants with polygenic score at 1st decile.

‡ 95% confidence interval for the odds ratio at 10th decile.

Supplementary Table 29. Summary statistics for UKB peptic ulcer diseases genome-wide associated significant SNPs (P < 5e-8) after mtCOJO analyses[23] conditional on UKB gastro-oesophageal reflux diseases GWAS summary statistics (P < 5e-8).

Trait	SNP	CHR.	BP	A1/A2	A1 frequency	UKB original statistics for PUD		UKB original statistics for GORD		UKB statistics after mtCOJO analyses[23] on GORD GWAS summary statistics	
						**OR***	**P**	**OR***	**P**	**OR***	**P**
PUD	rs681343	19	49206462	C/T	0.49	0.92	1.9E-15	1.00	7.8E-1	0.91	8.6E-16
	rs2976388	8	143760256	G/A	0.58	1.09	1.8E-14	1.02	2.3E-4	1.08	1.2E-12
	rs10500661	11	6273744	T/C	0.80	0.90	4.1E-14	0.98	2.1E-3	0.91	1.4E-12
	rs147048677	1	155161794	C/T	0.94	0.86	9.0E-12	1.00	7.6E-1	0.86	9.8E-12
	rs78459074	11	1029905	A/G	0.89	1.12	2.6E-10	0.99	1.6E-1	1.13	4.4E-11
	rs34074411	17	39867248	C/T	0.56	0.93	2.6E-10	1.00	7.4E-1	0.93	1.4E-10
	rs687621	9	136137065	A/G	0.68	1.08	1.3E-09	1.00	2.6E-1	1.07	3.0E-09
	rs9581957	13	28557889	C/T	0.68	0.93	3.6E-09	1.00	7.2E-1	0.93	2.0E-09

* Odds ratio (OR) is for risk of A1 allele compared to A2 allele.

Supplementary Figures

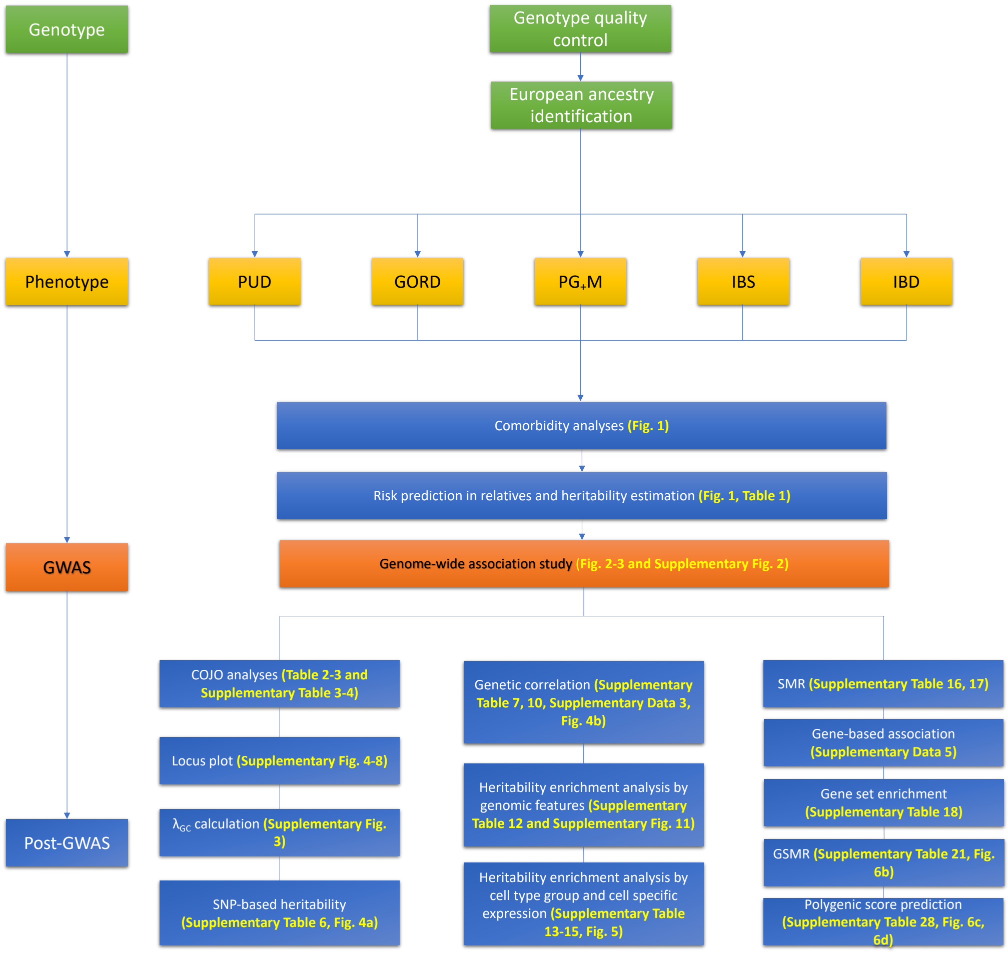

Supplementary Fig. 1. Full workflow of the study.

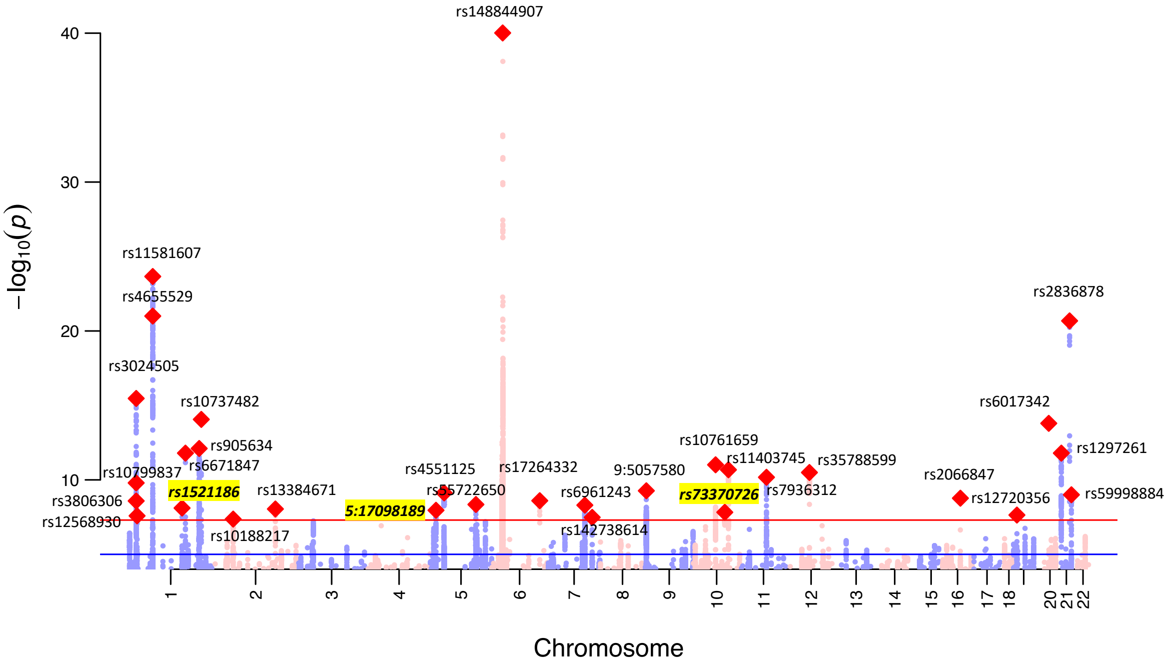

Supplementary Fig. 2. Manhattan plot for IBD. SNPs with red diamond represent genome-wide statistically significant independent loci (P < 5.0E-8) for each trait. SNPs highlighted with yellow colour, bold and italic font represent loci that haven't been reported to be associated with IBD.

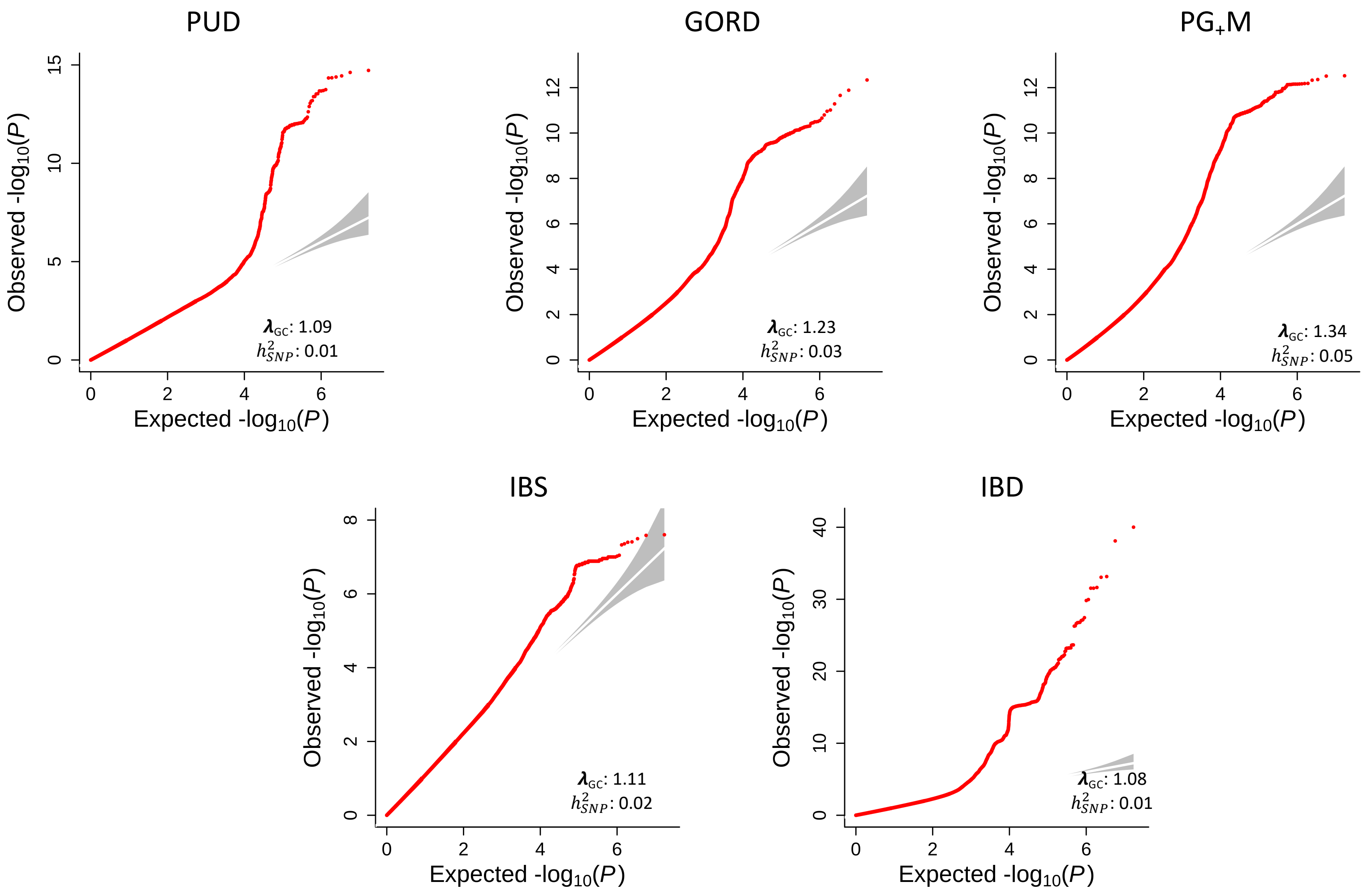

Supplementary Fig. 3. Quantile-Quantile (Q-Q) plots for the five digestion phenotypes. The λ_{GC} is directly calculated using median χ^2 on all the SNPs divided by 0.455 (the median χ^2 expected under the null hypothesis).

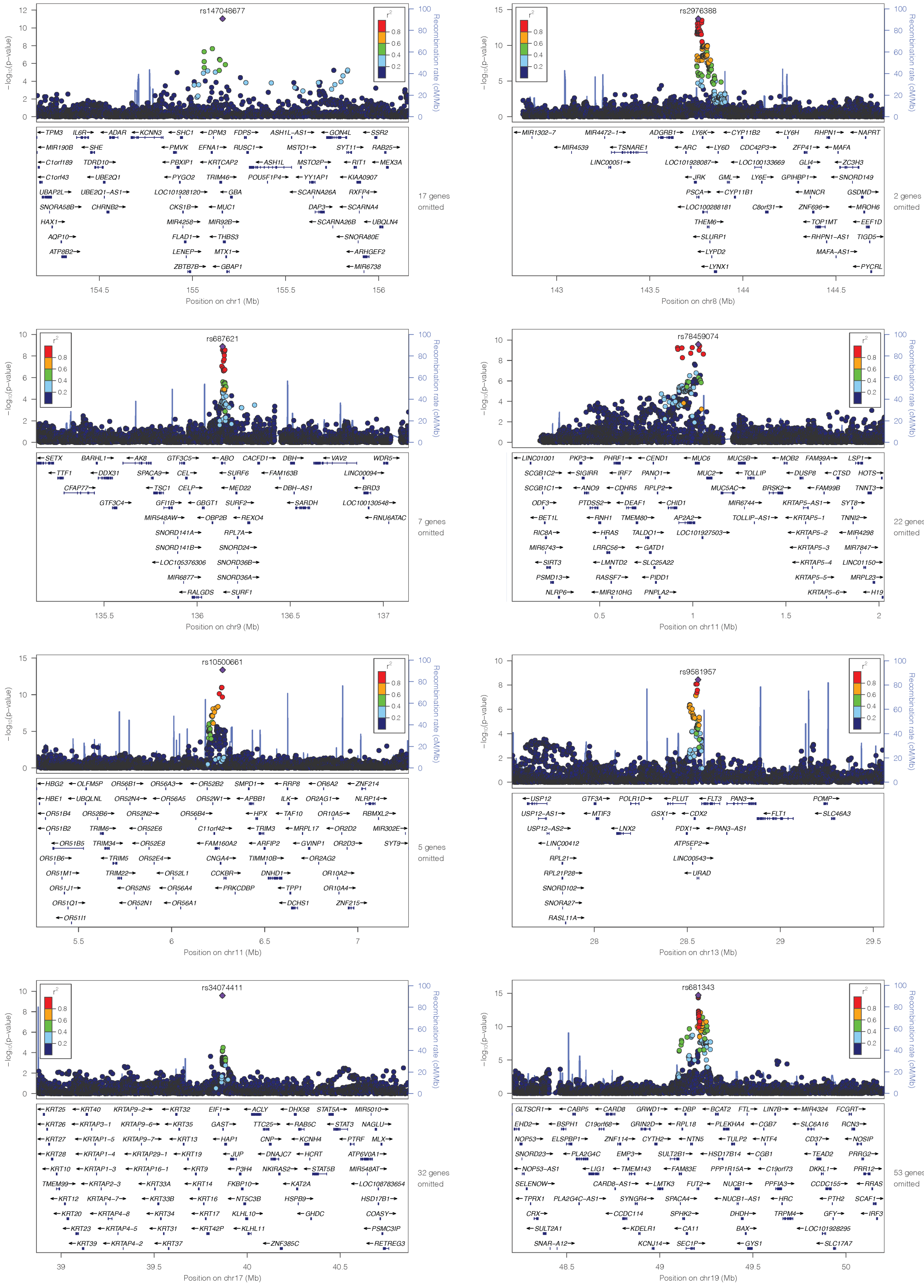

Supplementary Fig. 4. Regional association plot for peptic ulcer disease.

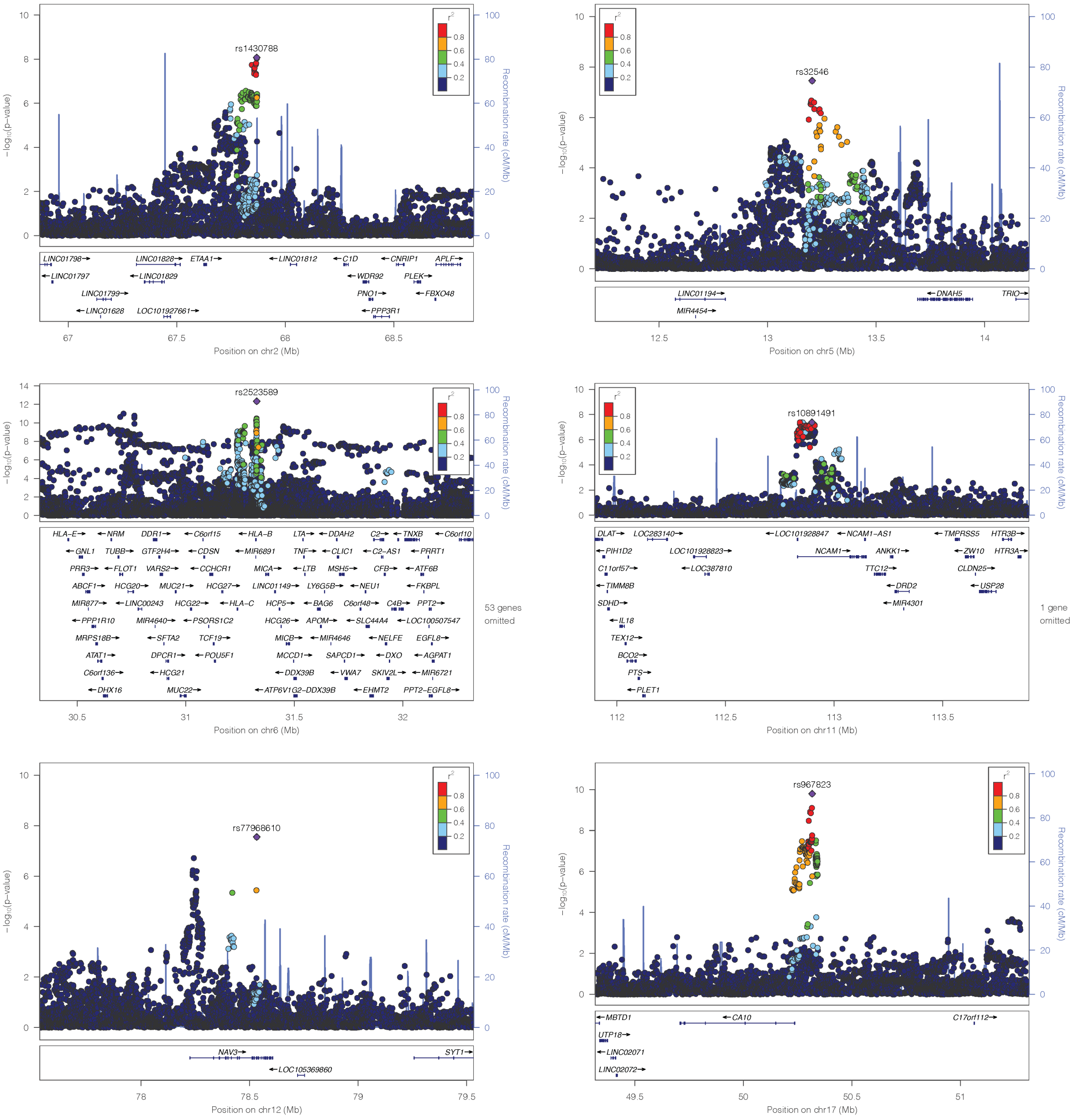

Supplementary Fig. 5. Regional association plots for gastroesophageal reflux disease.

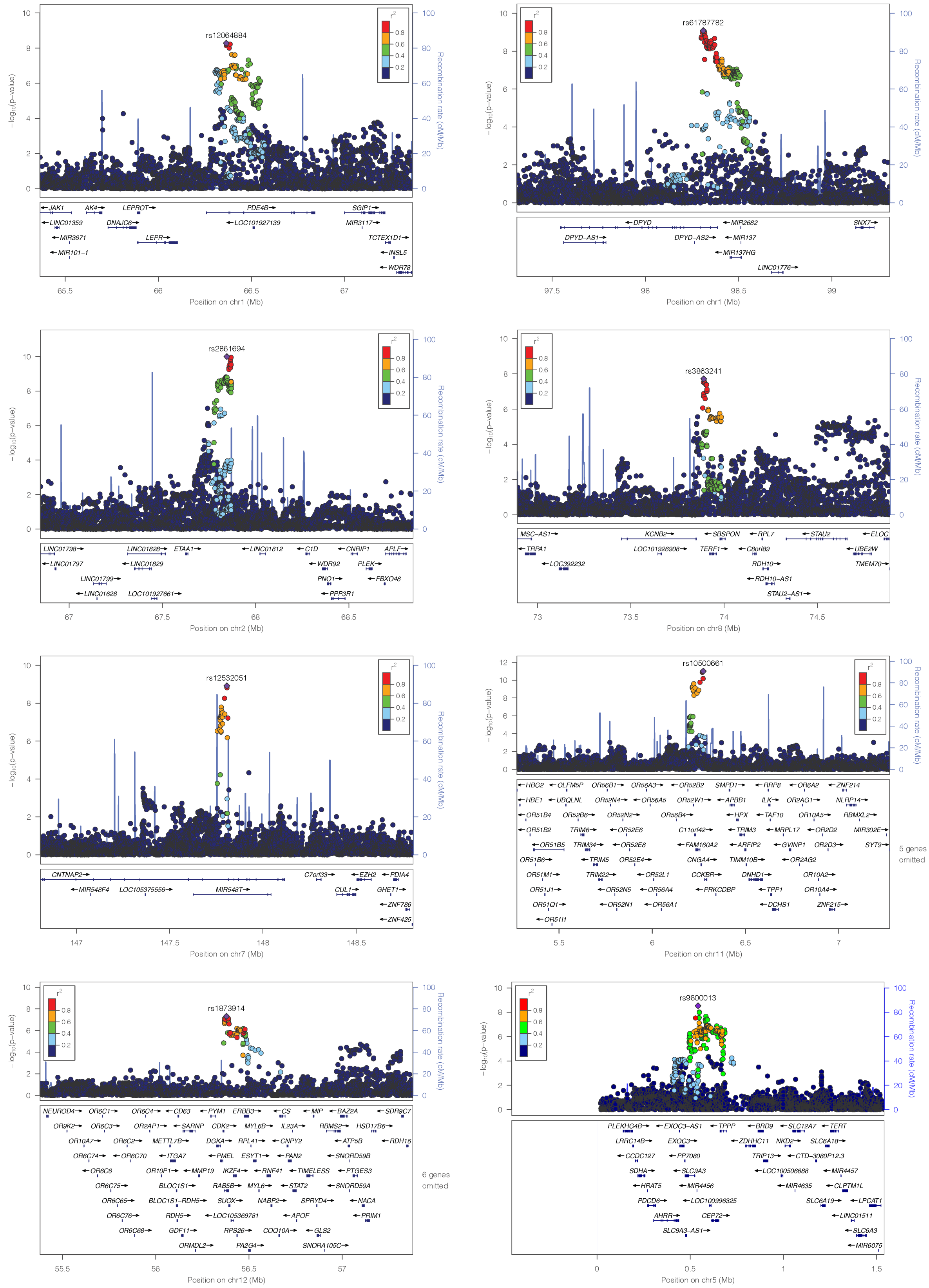
rs12064884
Position on chr1 (Mb)
JAK1 AK4 LEPROT PDE4B SGIP1
LINC01359 DNAJC6 LOC101927139 MIR3117
MIR3671 LEPR TCTEX1D1
MIR101-1 INSL5
WDR78
rs61787782
Position on chr1 (Mb)
DPYD SNX7
DPYD-AS1 DPYD-AS2 MIR2682 MIR137 MIR137HG
LINC01776
rs2861694
Position on chr2 (Mb)
LINC01798 LINC01828 ETAA1 LINC01812 C1D CNRIP1 APLF
LINC01797 LINC01829 WDR92 PLEK
LINC01799 PNO1 FBXO48
LINC01628 LOC101927661 PPP3R1
rs3863241
Position on chr8 (Mb)
MSC-AS1 KCNB2 SBSPON RPL7 STAU2 ELOC
TRPA1 LOC101926908 TERF1 C8orf89 UBE2W
LOC392232 RDH10 TMEM70
RDH10-AS1
STAU2-AS1
rs12532051
Position on chr7 (Mb)
CNTNAP2 C7orf33 EZH2 PDIA4
MIR548F4 LOC105375556 MIR548T CUL1 GHET1
ZNF786
ZNF425
rs10500661
Position on chr11 (Mb)
HBG2 OLFM5P OR56B1 OR56A3 OR52B2 SMPD1 RRP8 OR6A2 ZNF214
HBE1 UBQLNL OR52N4 OR56A5 OR52W1 APBB1 ILK OR2AG1 NLRP14
OR51B4 OR52B6 OR52N2 OR56B4 HPX TAF10 OR10A5 RBMXL2
OR51B2 TRIM6 OR52E6 C11orf42 TRIM3 MRPL17 OR2D2 MIR302E
OR51B5 TRIM34 OR52E8 FAM160A2 ARFIP2 GVINP1 OR2D3 SYT9
OR51B6 TRIM5 OR52E4 CNGA4 TIMM10B OR2AG2
OR51M1 TRIM22 OR52L1 CCKBR DNHD1 OR10A2
OR51J1 OR52N5 OR56A4 PRKCDBP TPP1 OR10A4
OR51Q1 OR52N1 OR56A1 DCHS1 ZNF215
OR51I1
5 genes omitted
rs1873914
Position on chr12 (Mb)
NEUROD4 OR6C1 OR6C4 CD63 PYM1 ERBB3 CS MIP BAZ2A SDR9C7
OR9K2 OR6C3 OR2AP1 SARNP CDK2 MYL6B IL23A RBMS2 HSD17B6
OR10A7 OR6C2 METTL7B DGKA RPL41 CNPY2 ATP5B RDH16
OR6C74 OR6C70 ITGA7 PMEL ESYT1 PAN2 SNORD59B
OR6C6 OR10P1 MMP19 IKZF4 RNF41 TIMELESS PTGES3
OR6C75 BLOC1S1 RAB5B MYL6 STAT2 SNORD59A
OR6C65 BLOC1S1-RDH5 SUOX NABP2 SPRYD4 NACA
OR6C76 RDH5 LOC105369781 APOF PRIM1
OR6C68 GDF11 RPS26 COQ10A GLS2
ORMDL2 PA2G4 SNORA105C
6 genes omitted
rs9800013
Position on chr5 (Mb)
PLEKHG4B EXOC3-AS1 TPPP BRD9 SLC12A7 TERT
LRRC14B EXOC3 ZDHHC11 NKD2 SLC6A18
CCDC127 PP7080 TRIP13 CTD-3080P12.3
SDHA SLC9A3 LOC100506688 MIR4457
HRAT5 MIR4456 MIR4635 CLPTM1L
PDCD6 LOC100996325 SLC6A19 LPCAT1
AHRR CEP72 LINC01511
SLC9A3-AS1 SLC6A3
MIR6075
−log10(p−value)
Recombination rate (cM/Mb)
r2

(Continued)

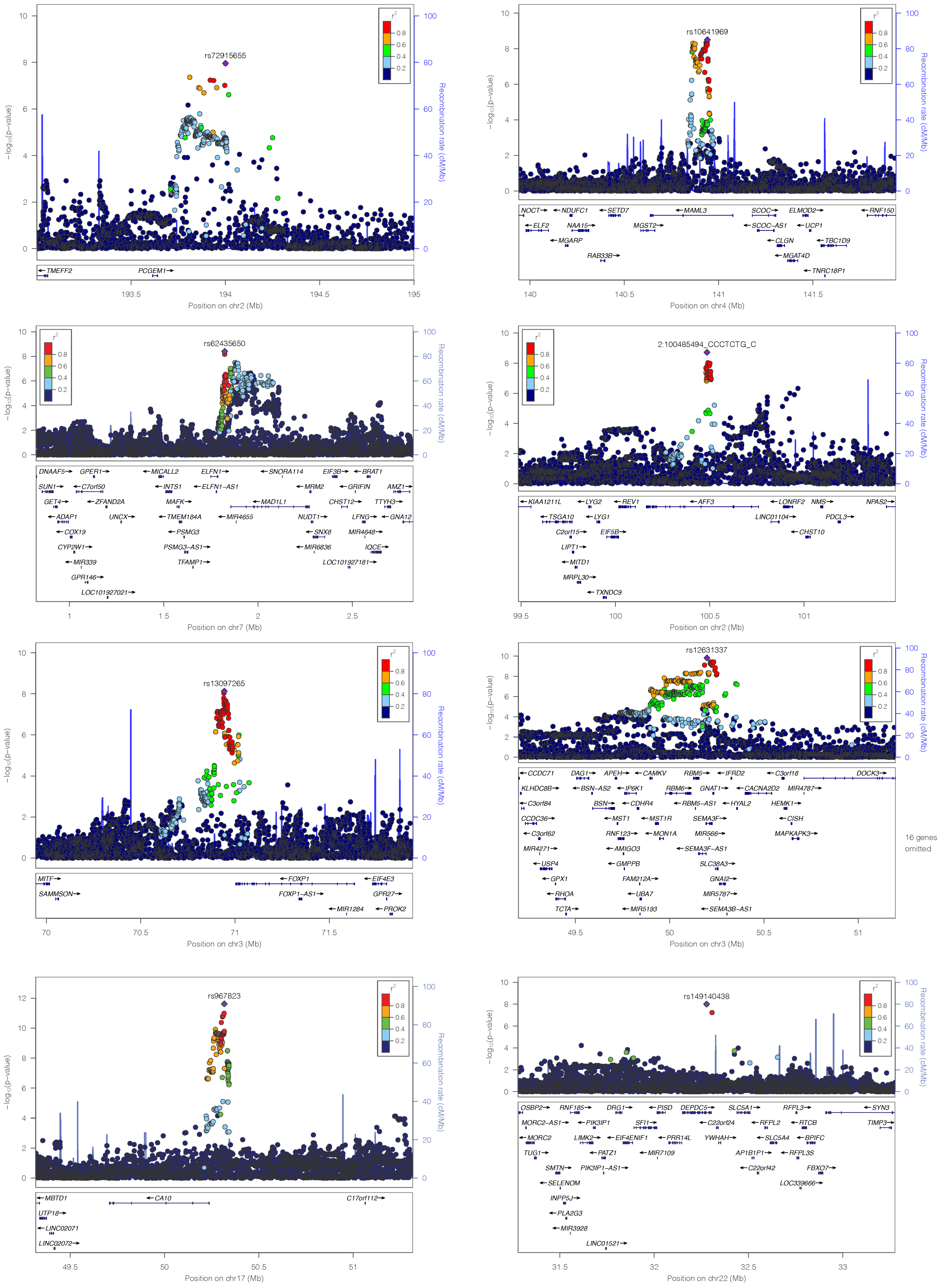
rs72915655
Position on chr2 (Mb)
TMEFF2
PCGEM1
rs10641969
Position on chr4 (Mb)
NOCT
NDUFC1
SETD7
MAML3
SCOC
ELMOD2
RNF150
ELF2
NAA15
MGST2
SCOC-AS1
UCP1
MGARP
CLGN
TBC1D9
RAB33B
MGAT4D
TNRC18P1
rs62435650
Position on chr7 (Mb)
DNAAF5
GPER1
MICALL2
ELFN1
SNORA114
EIF3B
BRAT1
SUN1
C7orf50
INTS1
ELFN1-AS1
MRM2
GRIFIN
AMZ1
GET4
ZFAND2A
MAFK
MAD1L1
CHST12
TTYH3
ADAP1
UNCX
TMEM184A
MIR4655
NUDT1
LFNG
GNA12
COX19
PSMG3
SNX8
MIR4648
CYP2W1
PSMG3-AS1
MIR6836
IQCE
MIR339
TFAMP1
LOC101927181
GPR146
LOC101927021
2:100485494_CCCTCTG_C
Position on chr2 (Mb)
KIAA1211L
LYG2
REV1
AFF3
LONRF2
NMS
NPAS2
TSGA10
LYG1
LINC01104
PDCL3
C2orf15
EIF5B
CHST10
LIPT1
MITD1
MRPL30
TXNDC9
rs13097265
Position on chr3 (Mb)
MITF
FOXP1
EIF4E3
SAMMSON
FOXP1-AS1
GPR27
MIR1284
PROK2
rs12631337
Position on chr3 (Mb)
CCDC71
DAG1
APEH
CAMKV
RBM5
IFRD2
C3orf18
DOCK3
KLHDC8B
BSN-AS2
IP6K1
RBM6
GNAT1
CACNA2D2
MIR4787
C3orf84
BSN
CDHR4
RBM5-AS1
HYAL2
HEMK1
CCDC36
MST1
MST1R
SEMA3F
CISH
C3orf62
RNF123
MON1A
MIR566
MAPKAPK3
MIR4271
AMIGO3
SEMA3F-AS1
USP4
GMPPB
SLC38A3
GPX1
FAM212A
GNAI2
RHOA
UBA7
MIR5787
TCTA
MIR5193
SEMA3B-AS1
16 genes omitted
rs967823
Position on chr17 (Mb)
MBTD1
CA10
C17orf112
UTP18
LINC02071
LINC02072
rs149140438
Position on chr22 (Mb)
OSBP2
RNF185
DRG1
PISD
DEPDC5
SLC5A1
RFPL3
SYN3
MORC2-AS1
PIK3IP1
SFI1
C22orf24
RFPL2
RTCB
TIMP3
MORC2
LIMK2
EIF4ENIF1
PRR14L
YWHAH
SLC5A4
BPIFC
TUG1
PATZ1
MIR7109
AP1B1P1
RFPL3S
SMTN
PIK3IP1-AS1
C22orf42
FBXO7
SELENOM
LOC339666
INPP5J
PLA2G3
MIR3928
LINC01521
−log10(p-value)
Recombination rate (cM/Mb)
r²

(Continued)

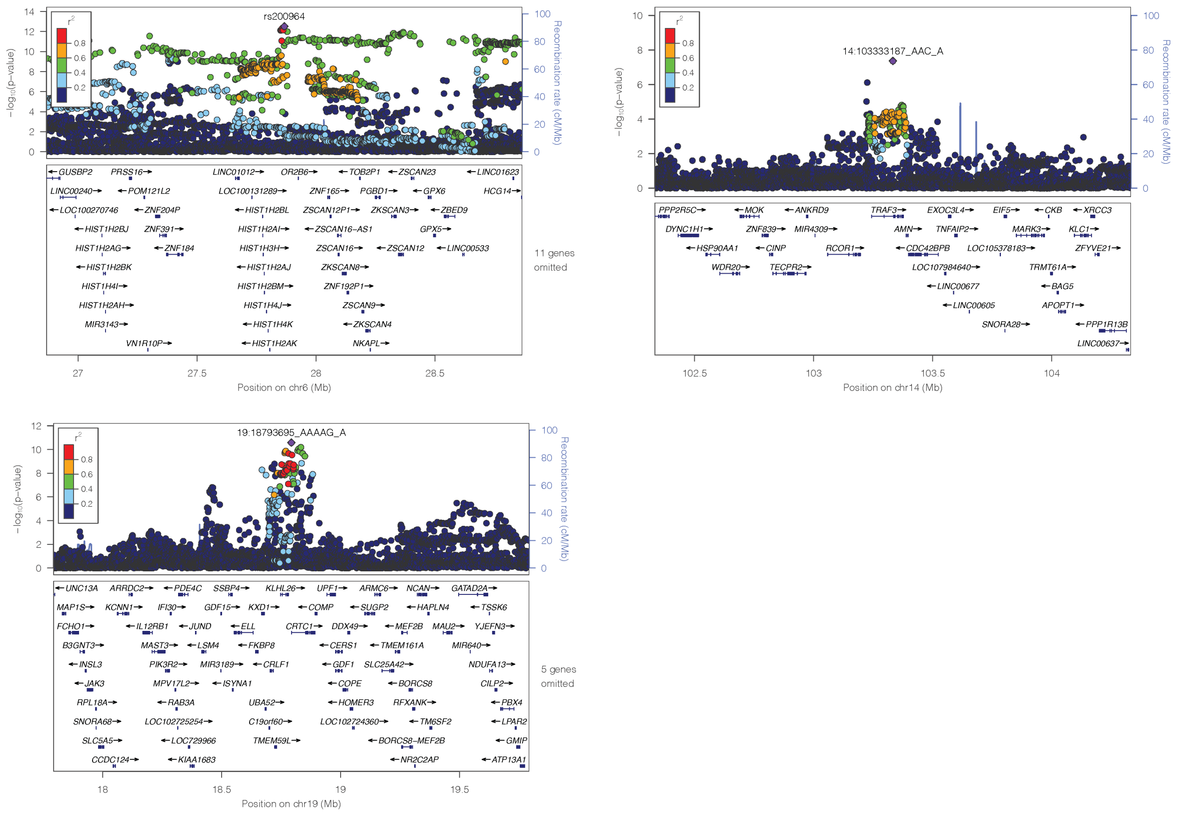

Supplementary Fig. 6. Regional association plots of peptic ulcer disease, gastroesophageal reflux disease and the corresponding medications .

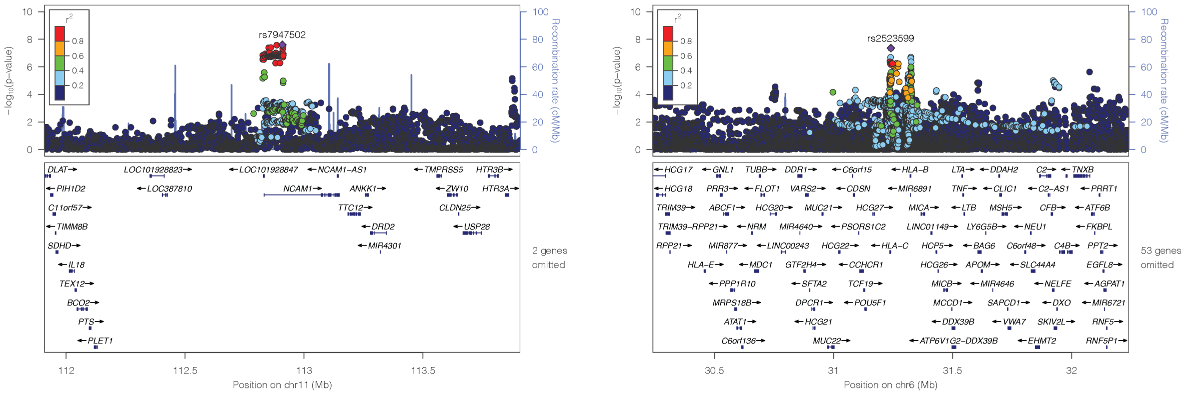

Supplementary Fig. 7. Regional association plot of irritable bowel syndrome.

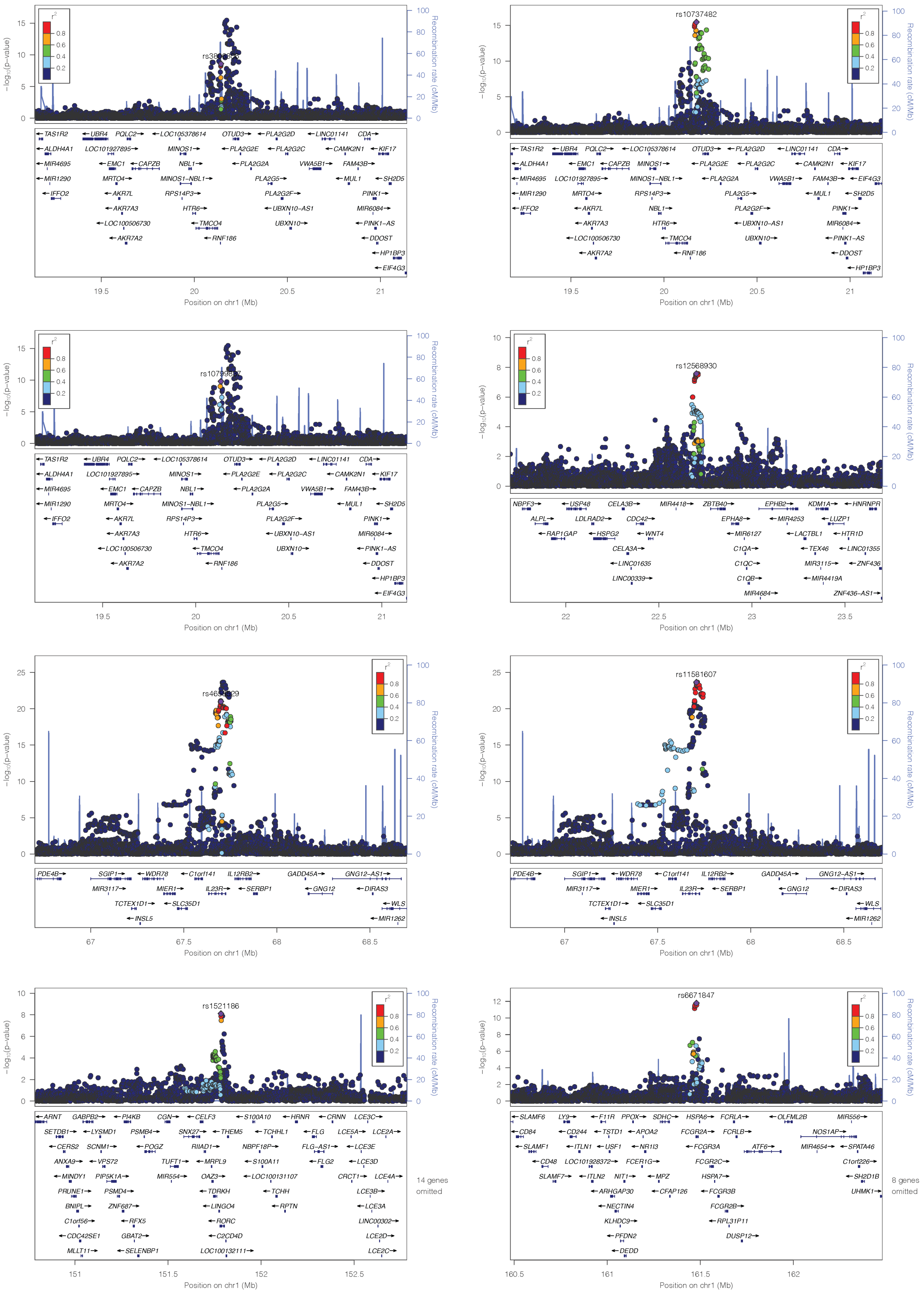
rs10737482
rs12568930
rs11581607
rs1521186
rs6671847
−log10(p−value)
Recombination rate (cM/Mb)
Position on chr1 (Mb)
14 genes omitted
8 genes omitted

(Continued)

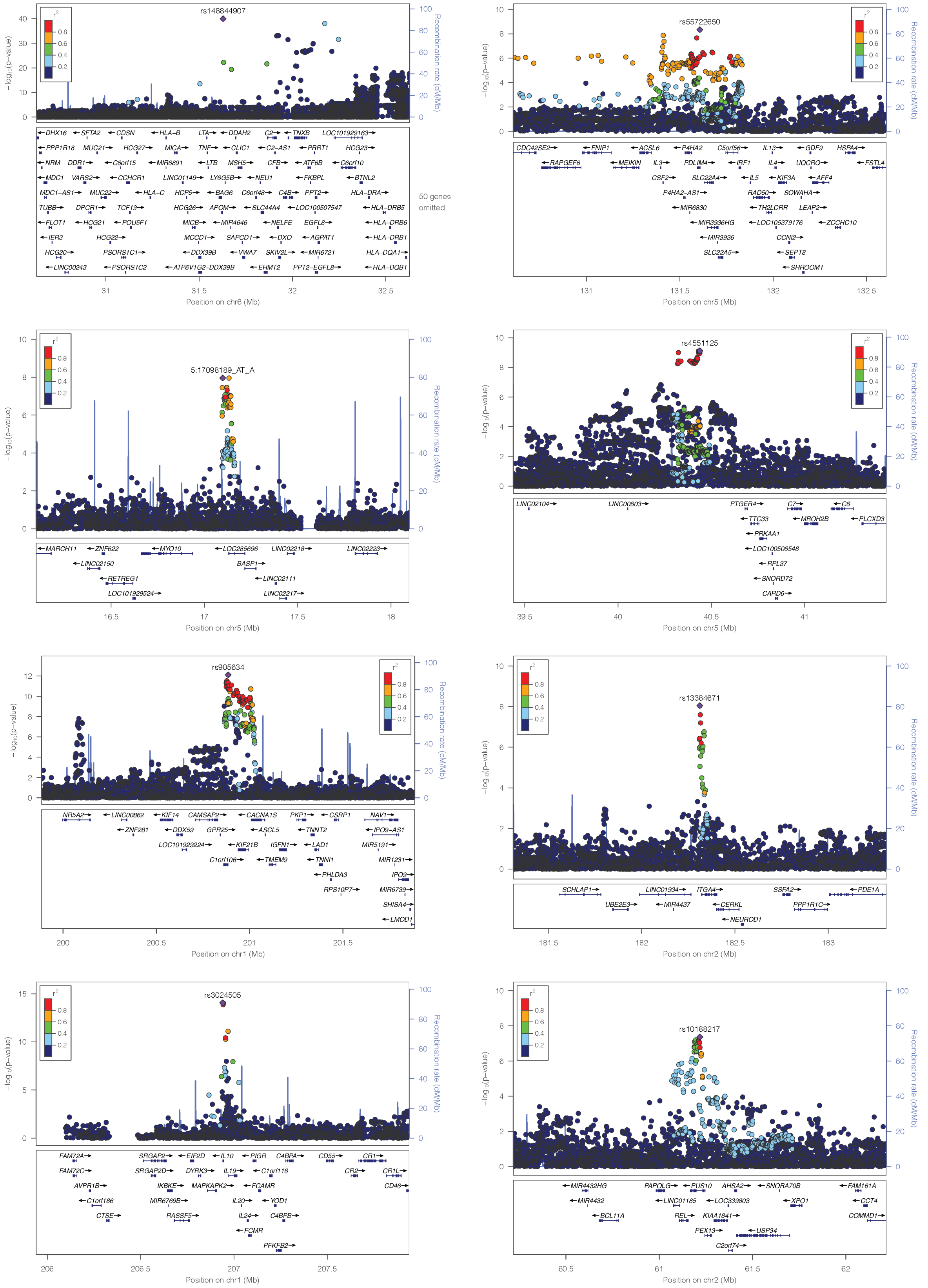

rs148844907
−log10(p-value)
Recombination rate (cM/Mb)
r2
0.8
0.6
0.4
0.2
50 genes omitted
Position on chr6 (Mb)
rs55722650
Position on chr5 (Mb)
5:17098189_AT_A
Position on chr5 (Mb)
rs4551125
Position on chr5 (Mb)
rs905634
Position on chr1 (Mb)
rs13384671
Position on chr2 (Mb)
rs3024505
Position on chr1 (Mb)
rs10188217
Position on chr2 (Mb)

(Continued)

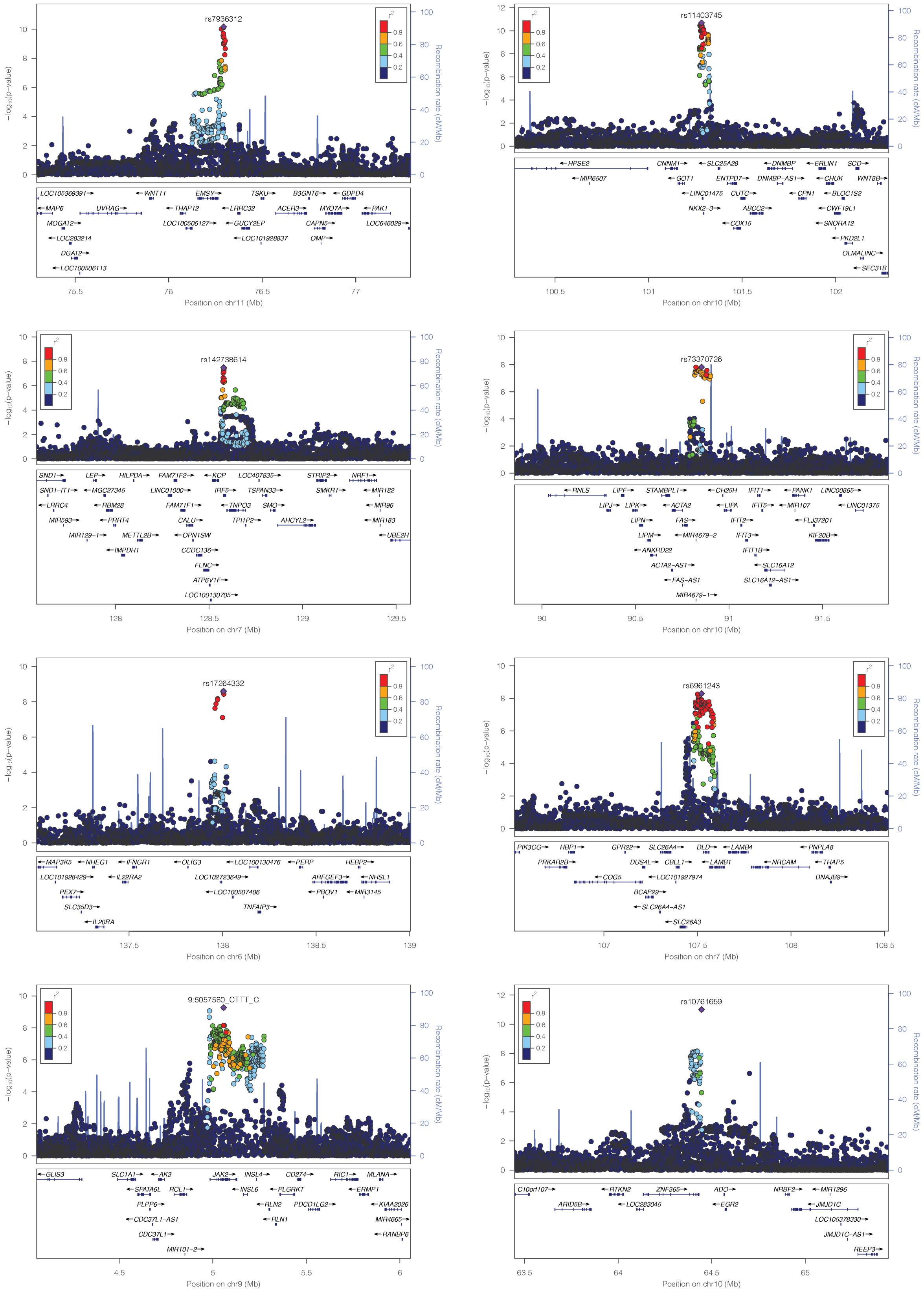
rs7936312
Position on chr11 (Mb)
rs11403745
Position on chr10 (Mb)
rs142738614
Position on chr7 (Mb)
rs73370726
Position on chr10 (Mb)
rs17264332
Position on chr6 (Mb)
rs6961243
Position on chr7 (Mb)
9:5057580_CTTT_C
Position on chr9 (Mb)
rs10761659
Position on chr10 (Mb)
−log10(p−value)
Recombination rate (cM/Mb)
r2

(Continued)

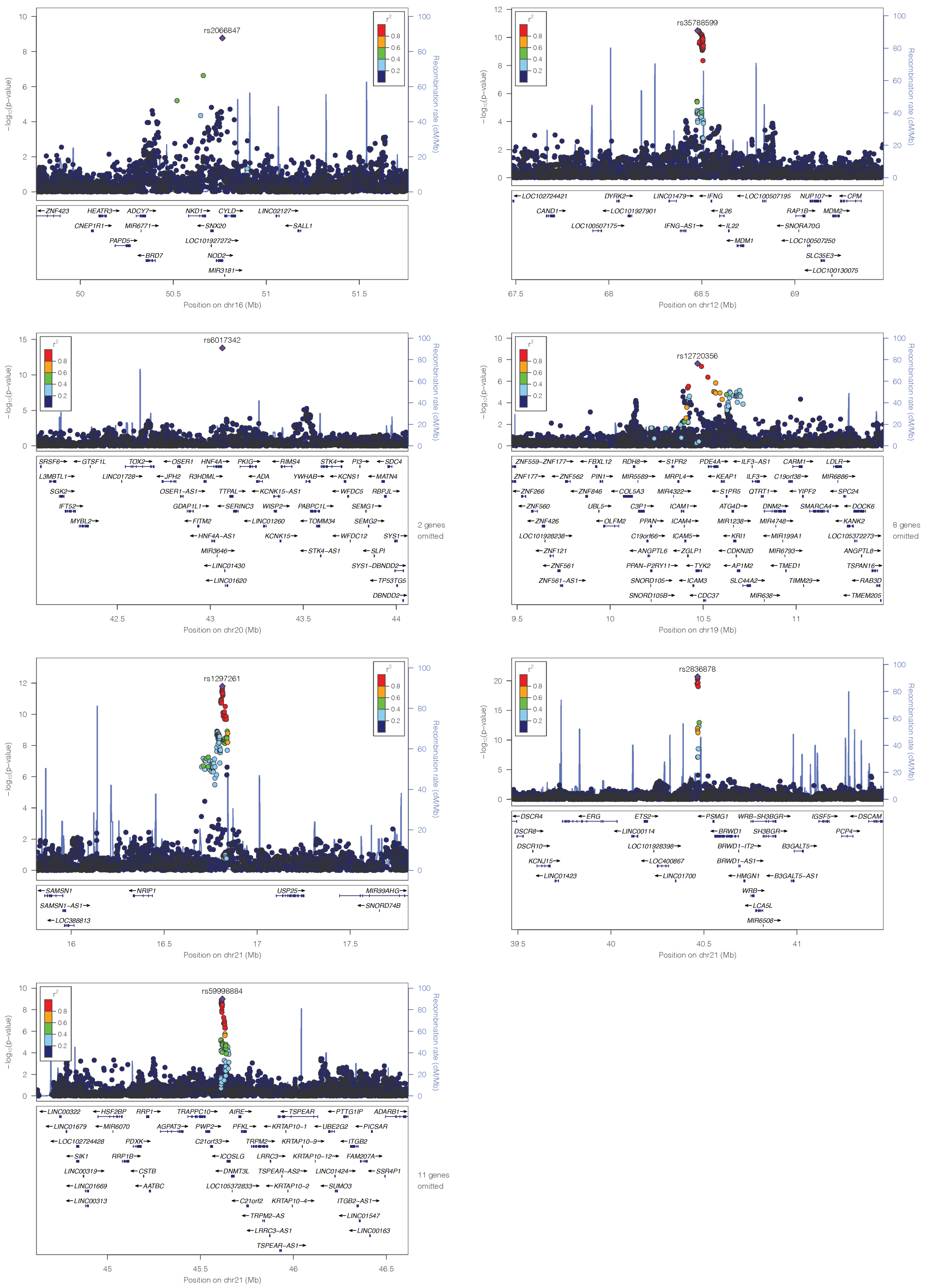

Supplementary Fig. 8. Regional association plot of inflammatory bowel diseases.

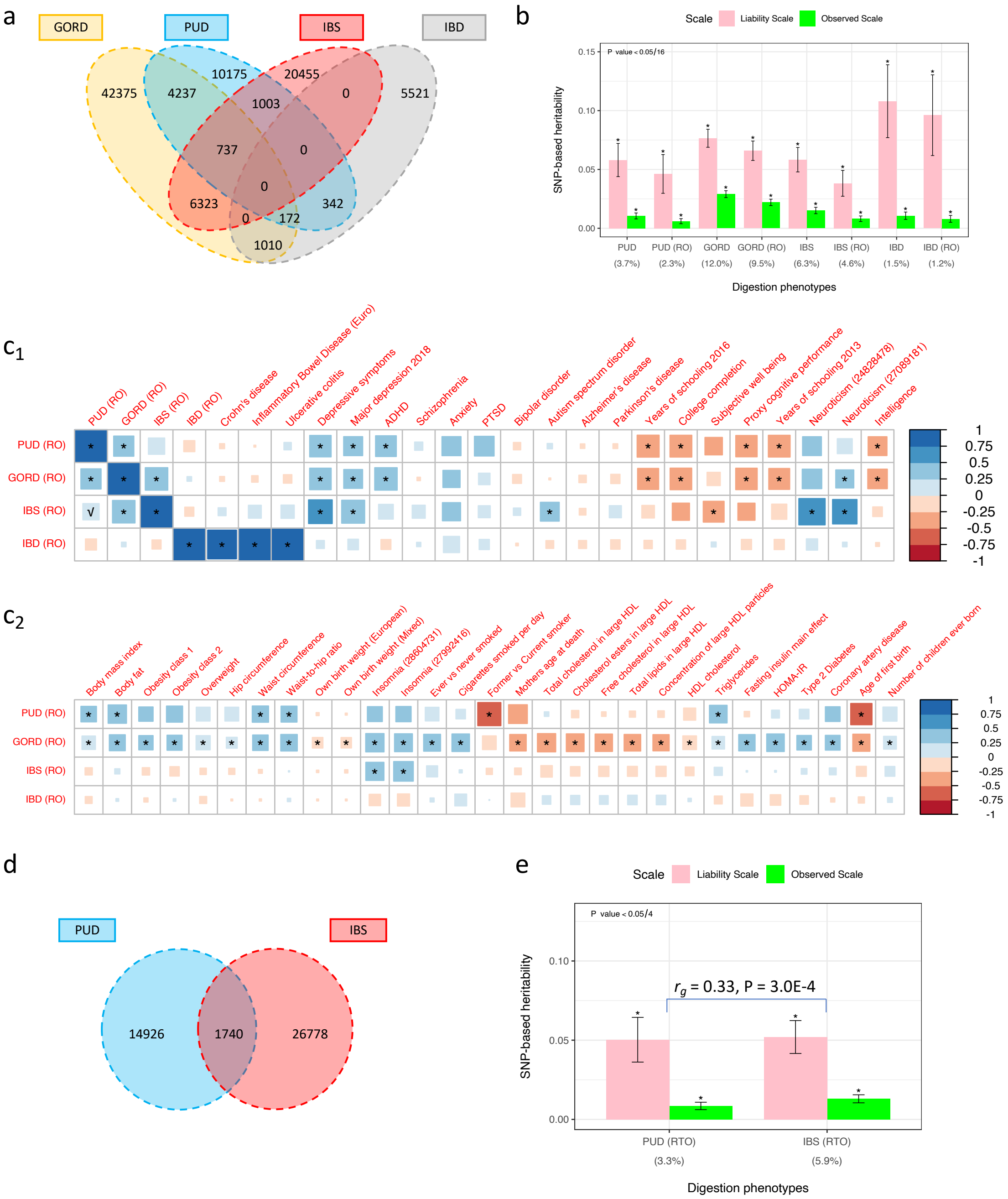

Supplementary Fig. 9. SNP-based heritability and genetic correlation for PUD, GORD, IBS and IBD after removing the overlapped individuals using LD score regression analyses[14,15]. **Panel a**. Venn diagram for the number of overlapped individuals among PUD, GORD, IBS and IBD cases. **Panel b**. Comparison of SNP-based heritability on observed and liability scale for PUD, GORD, IBS and IBD between the original phenotypes and phenotypes generated after removing individuals with more than one disorder (defined as sensitivity analysis phenotypes). "RO" represents removing overlapped individuals with more than one disorder. We took sample risk, i.e. the proportion cases for each phenotype in the UKB cohort, as the population lifetime risk to calculate the SNP-based heritability on the liability scale for each digestion phenotype; the sample risk percentage is shown below x axis. The error bars represent 95% confidence intervals for the estimated SNP-based heritability. "*" represents that the SNP-based heritability was still significant after Bonferroni correction ($P < 0.05/16$). **Panel c**. Genetic correlation between sensitivity analysis phenotypes with the original phenotypes, within sensitivity analysis phenotypes, between sensitivity analysis phenotypes with traits from LD Hub and published neuro-psychiatric disorder studies. "*" represents that genetic correlation estimate was still significant after Bonferroni correction ($P < 0.05/(4*4+4*258+4*9)$) while "√" represents the P value for genetic correlation between IBS (RO) and PUD (RO) estimates < 0.05. **Panel d**. The number of overlapped individuals between PUD and IBS cases. **Panel e**. SNP-based heritability on observed and liability scale for PUD and IBS

phenotypes generated after removing the 1740 individuals with both PUD and IBS. “RTO” represents removing the 1740 individuals with both PUD and IBS. The sample risk percentage, as shown below axis, was used as the population lifetime risk to calculate the SNP-based heritability on the liability scale. The error bars represent 95% CIs for the estimated SNP-based heritability.

a

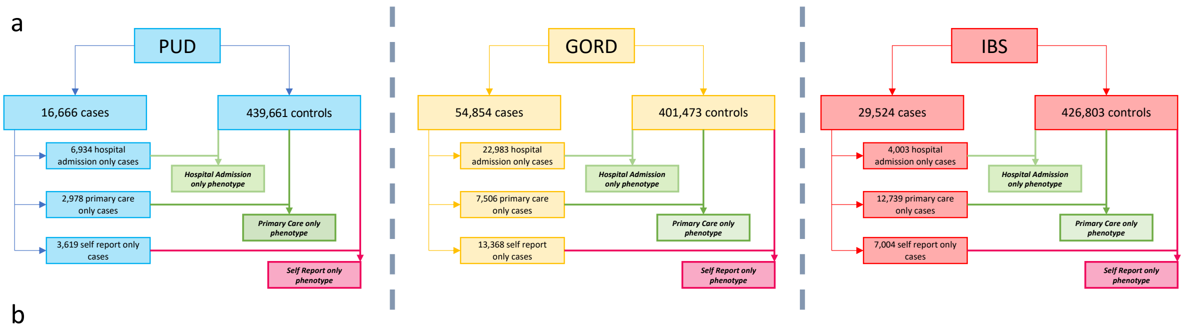

b

SNP	Trait	CHR	BP	A1/A2	A1 frequency	BETA	SE	P	P for male GWAS	P for female GWAS
rs10512344	IBS Hospital Admission	9	109009495	G/C	0.96	-0.038	0.056	5.0E-01	-	-
	IBS Primary Care					-0.030	0.032	3.5E-01	-	-
	IBS Self Report					-0.157	0.040	9.1E-05	-	-
	IBS					-0.085	0.021	5.0E-05	0.73	4.4E-07

c

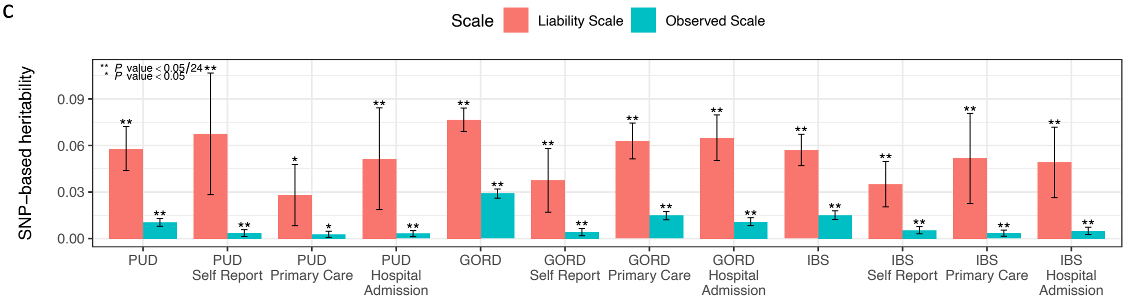

d

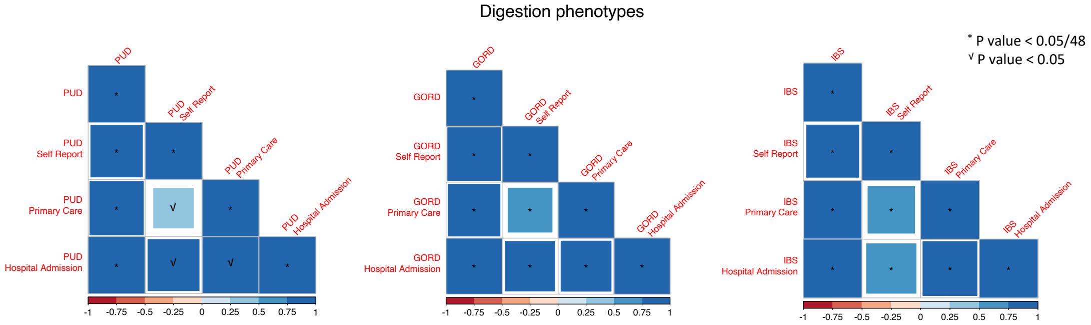

e

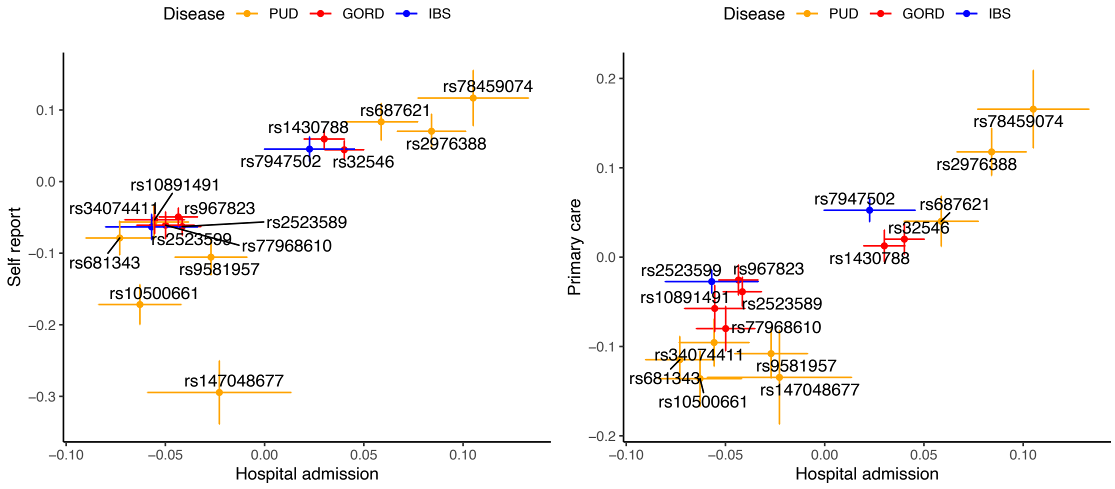

Supplementary Fig. 10a. The process for generation of subgroup phenotypes for each of PUD, GORD and IBS. **Supplementary Fig. 10b.** Summary statistics for rs10512344 from GWAS analyses of IBS and the three subgroup phenotypes of IBS using BOLT-LMM[11]. **Supplementary Fig. 10c.** SNP-based heritability estimates and 95% confidence intervals from LD score regression[14] for self-report, primary care and hospital admission subgroup phenotypes and the original phenotype for each of PUD, GORD and IBS. “*” represents that the

statistical P value for the corresponding SNP-based heritability less than 0.05 and "**" represents that the statistical P value for the corresponding SNP-based heritability less than 0.05/24 (after Bonferroni correction). **Supplementary Fig. 10d**. Genetic correlation among self-report, primary care, hospital admission subgroup phenotypes and the original phenotype for each of PUD, GORD and IBS using bivariate LD score regression[15]. **Supplementary Fig. 10e**. The effect size of genome-wide significant SNPs from broad definition of PUD, GORD and IBS in hospital admission subgroup phenotype versus those in self report subgroup phenotype and primary care subgroup phenotype, respectively. Due to the whole-part relationships for each of broadly defined phenotype and the corresponding three subgroup phenotypes, as expected, the effect size estimates for the SNPs associated with broadly defined phenotype showed high concordance in the GWAS summary statistics of the corresponding three subgroup phenotypes.

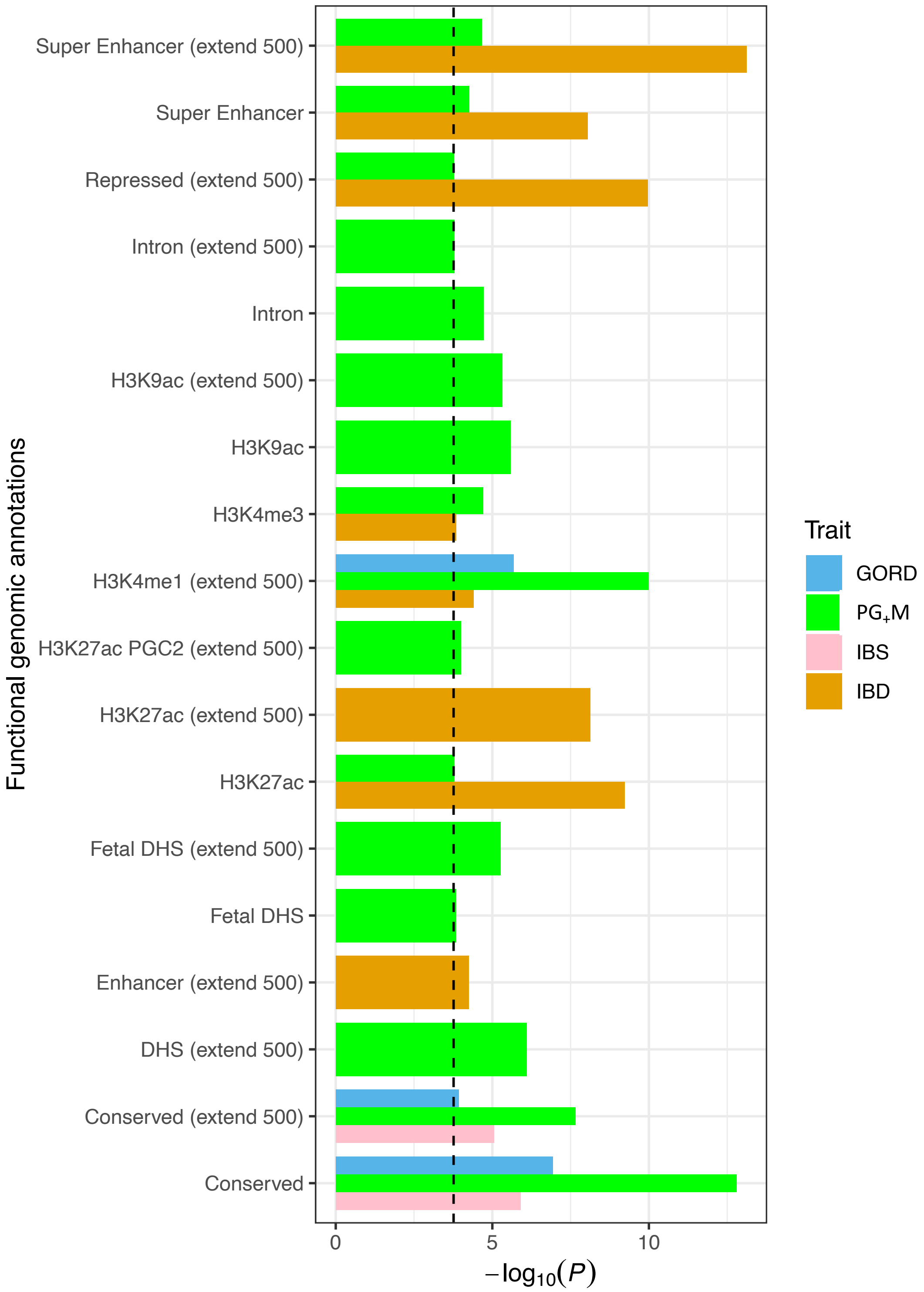

Supplementary Fig. 11. Significant heritability enrichment for GORD, PG+M and IBD of functional annotation based on the variants within each category after Bonferroni correction (P < 0.05/(53*5+5*5)). The non-significant annotations are not shown.

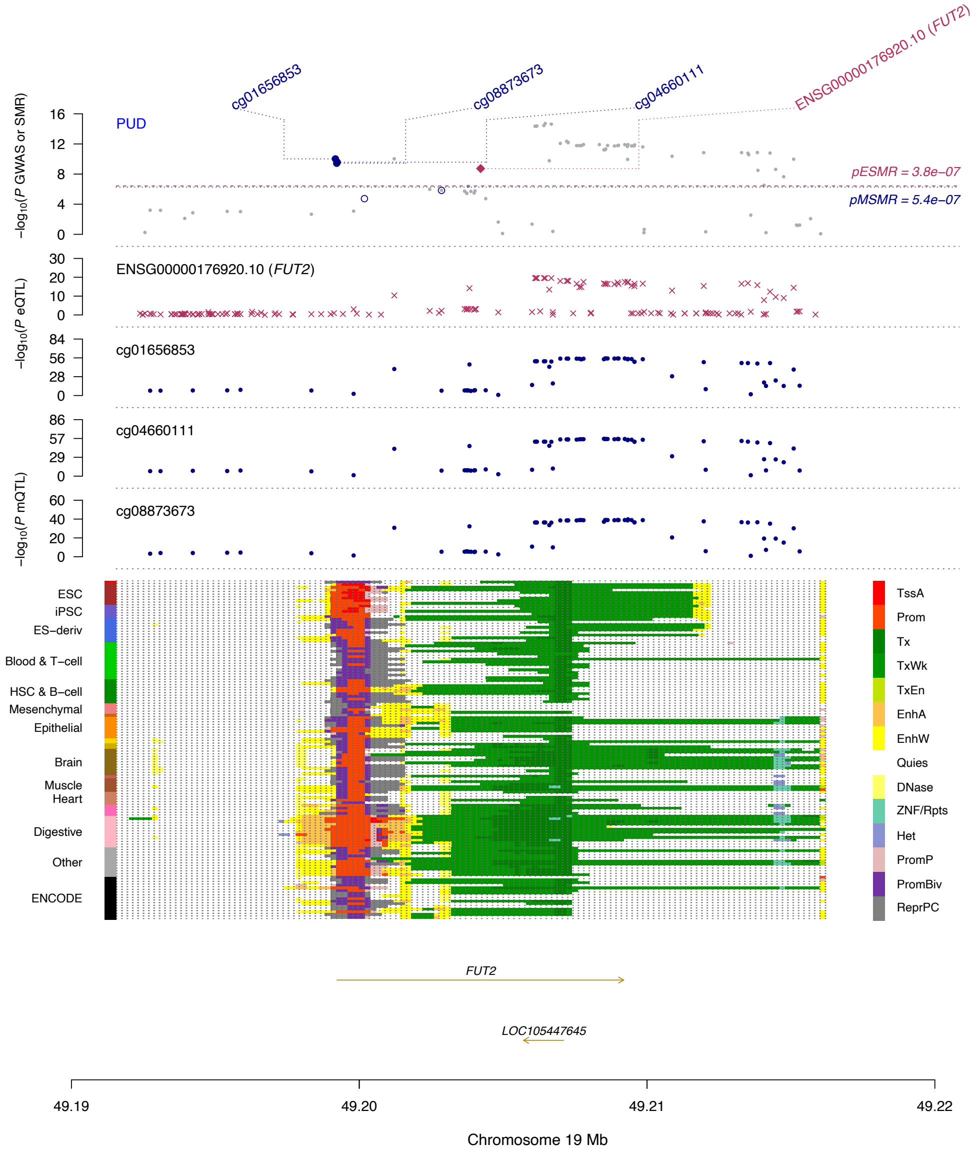

Supplementary Fig. 12. Results of SNP and SMR associations across mQTL, eQTL and GWAS for *FUT2* gene of PUD. The top plot of shows -$\log_{10}$(P) of SNPs from PUD GWAS. The red diamonds and blue circles represent -$\log_{10}$(P) from SMR test for associations of gene expression and DNAm probes with PUD, respectively. The solid diamonds and circles are probes not rejected by the HEIDI test. The second plot shows -$\log_{10}$(P) of the SNP association for gene expression *FUT2* from the stomach eQTL GTEx[25] data. The third plot shows -$\log_{10}$(P) of the SNP associations for DNA methylation probe cg01656853, cg04660111 and cg08873673 from blood mQTL data of McRae *et al.*[19] (there are no public digestive mQTL data at present). The bottom plot shows Roadmap epigenomics annotation (indicated by colours) for different primary cells and tissue types (rows).

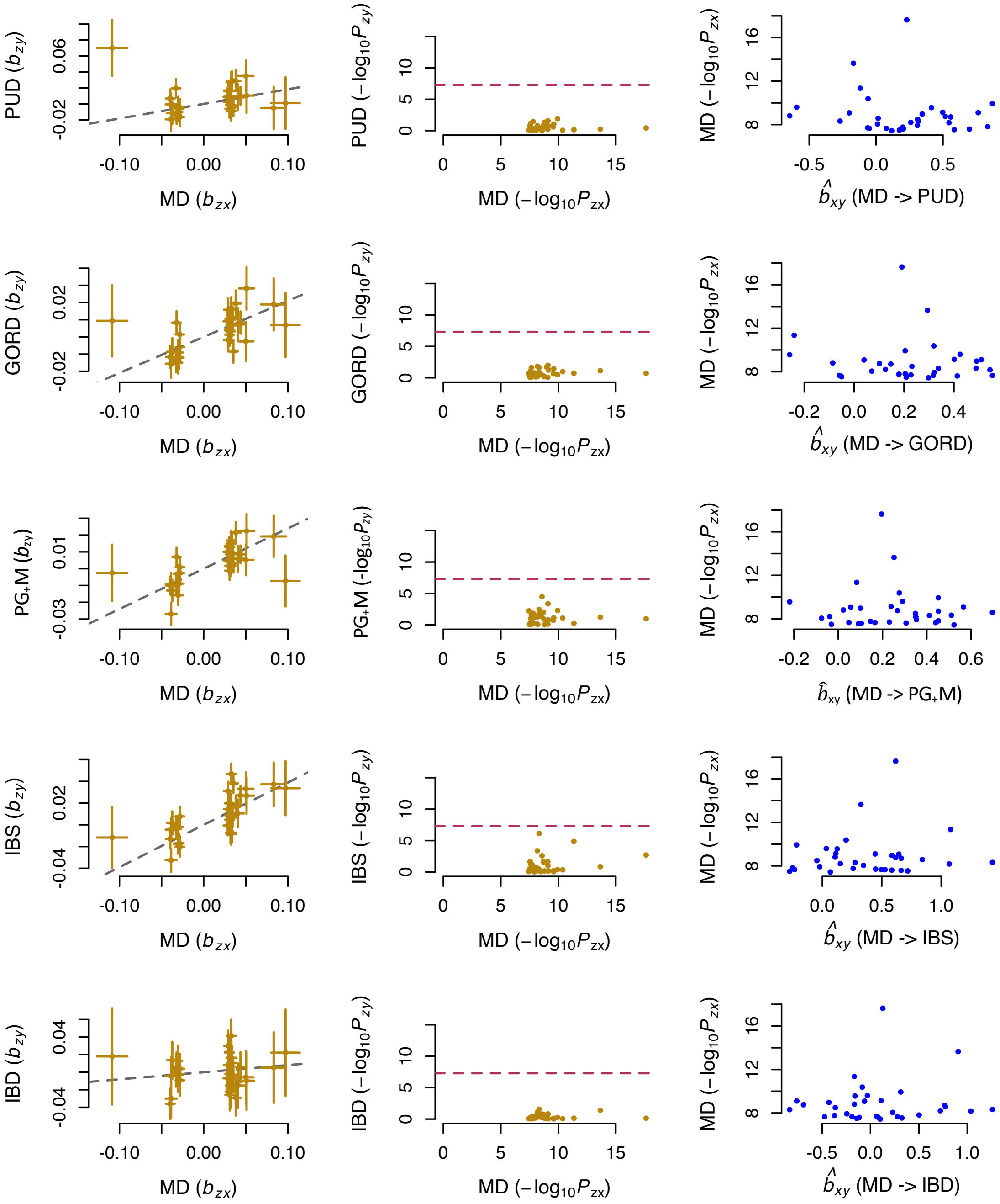

Supplementary Fig. 13. Plots of the effect sizes of exposure SNPs (x-axis) vs. outcome SNPs (y-axis) (first column) and association P values of all the genetic instruments from GWAS for major depression (MD) vs. those for each of the five digestion phenotypes (second column). Shown in the third column are the plots of b_{xy} vs. GWAS P value of MD at each genetic variant. All the exposure SNPs were obtained after HEIDI outlier test.

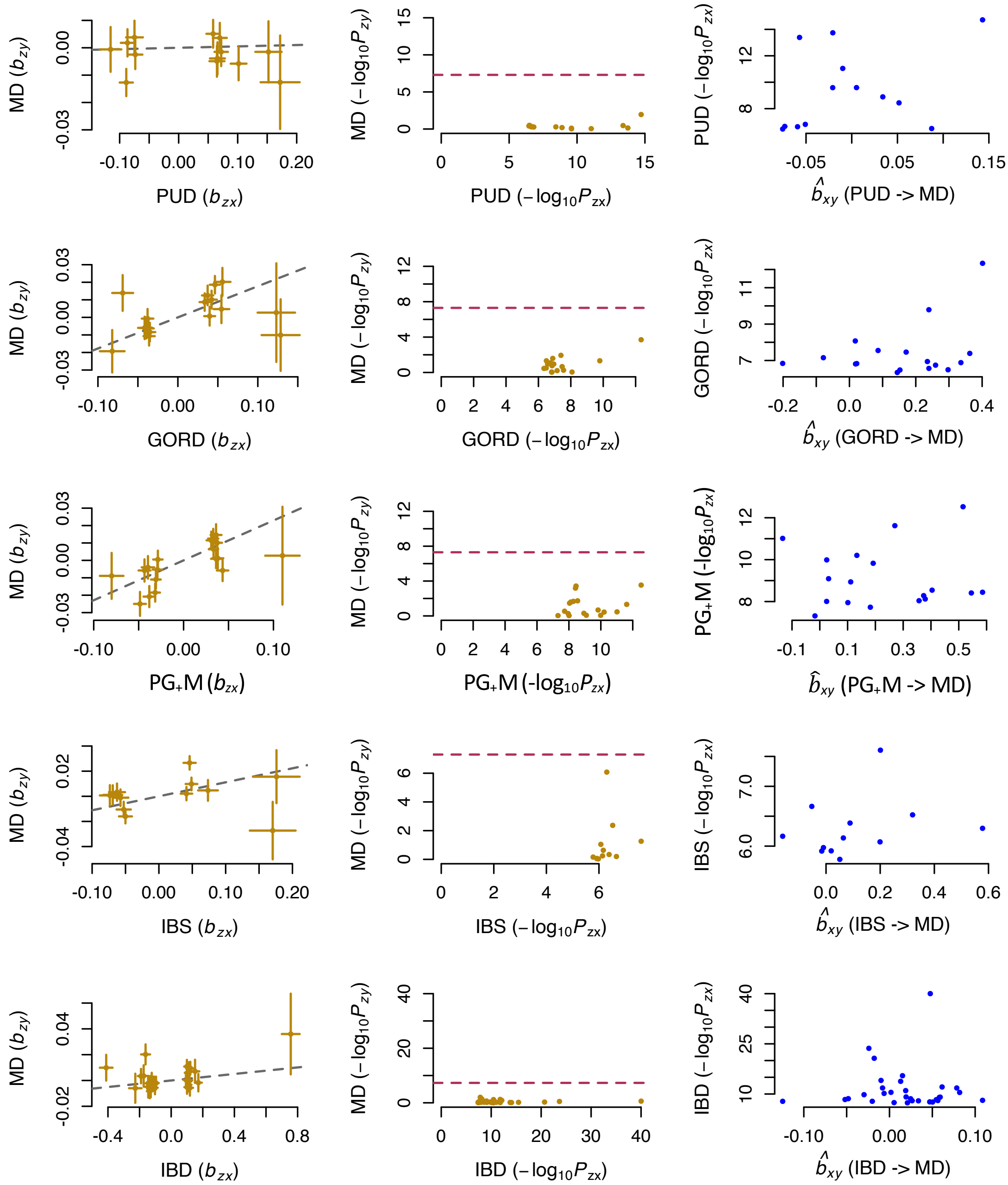

Supplementary Fig. 14. Plots of the effect sizes of exposure SNPs (x-axis) vs. outcome SNPs (y-axis) (first column) and association P values of all the genetic instruments from GWAS for each of the five digestion phenotypes vs. those for major depression (MD) (second column). Shown in the third column are the plots of b_{xy} vs. GWAS P value of the five digestion phenotypes at each genetic variant. All the exposure SNPs were obtained after HEIDI outlier test. For PUD, GORD and IBS, we relaxed the significance threshold to obtain more SNP instruments.

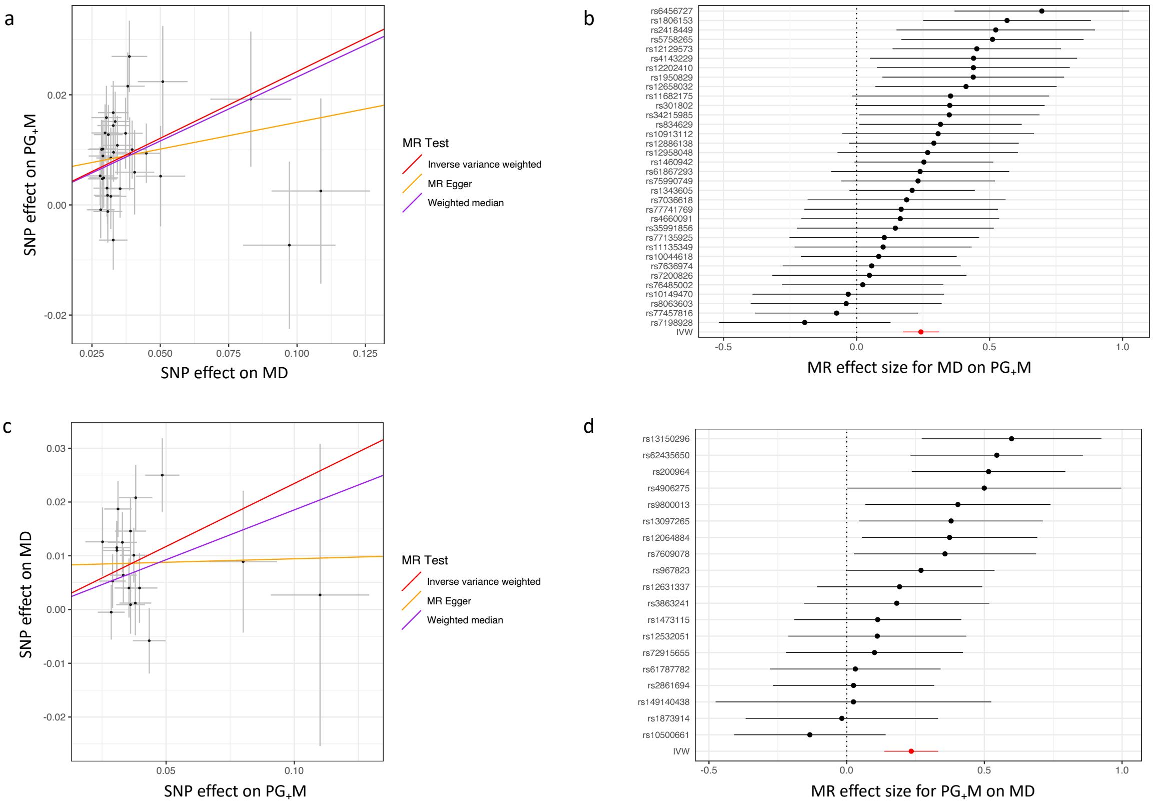

Supplementary Fig. 15. Mendelian Randomization (MR) plots between major depression and PG+M. **Panel a.** Scatterplot of single-nucleotide polymorphism (SNP) potential effects of major depression on PG+M, with the slope of each line corresponding to estimated MR effect per method. **Panel b.** Forest plot of individual and combined SNP MR-estimated effect sizes and 95% confidence intervals (CIs)of major depression on PG+M. The raw effect sizes with 95% confidence interval are presented as the dot and horizontal line. **Panel c.** Scatterplot of SNP potential effects of PG+M on major depression, with the slope of each line corresponding to estimated MR effect per method. **Panel d.** Forest plot of individual and combined SNP MR-estimated effect sizes and 95% CIs of PG+M on major depression. The raw effect sizes with 95% confidence interval are presented as the dot and horizontal line.

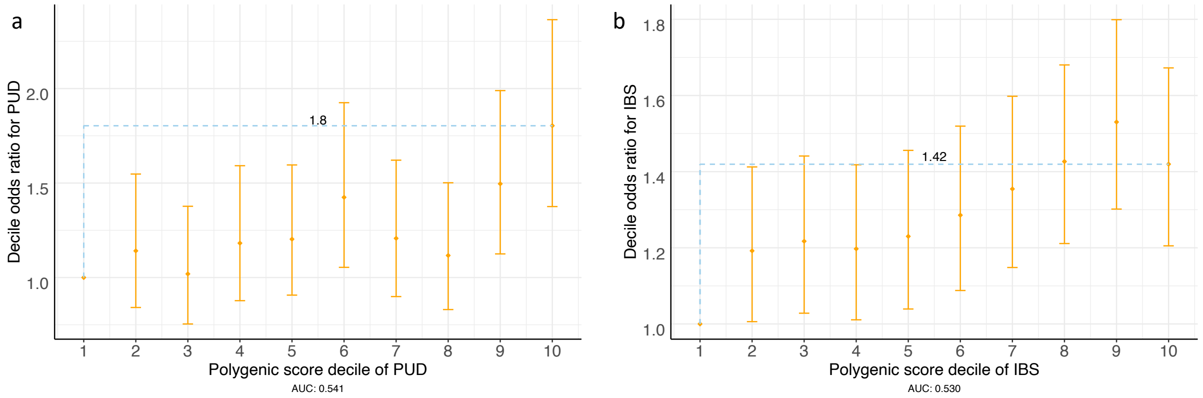

Supplementary Fig. 16. Polygenic score of peptic ulcer disease (PUD) and irritable bowel syndrome (IBS) predicts odds ratio (OR) for PUD (**Panel a**) and IBS (**Panel b**) respectively in individuals from GERA cohort using logistic regression model. Polygenic score of individuals from GERA cohort were calculated based on PUD associated SNPs with P < 5.0E-8 and IBS associated SNPs with P < 0.1 from UKB and converted to deciles (1 = lowest, 10 = highest). OR and 95% confidence intervals (CI, orange diamonds and bars) relative to decile 1 were estimated using logistic regression. The blue dashed lines in **a** represent that compared with the lowest decile, the highest decile have an OR of 1.80 for PUD. The number of PUD cases and controls from GERA cohort were 1,004 and 60,843, respectively. The P value for case-control PUD polygenic score difference from GERA cohort is 2.5E-6. The blue dashed lines in **b** represent that compared with the lowest decile, the highest decile have an OR of 1.42 for IBS. The number of IBS cases and controls from GERA cohort were 3,359 and 58,488, respectively. The P value for case-control IBS polygenic score difference from GERA cohort is 5.4E-8.

References:

1. Bonfiglio, F. *et al.* Female-Specific Association Between Variants on Chromosome 9 and Self-Reported Diagnosis of Irritable Bowel Syndrome. *Gastroenterology* **155**, 168–179 (2018).
2. van Rheenen, W., Peyrot, W. J., Schork, A. J., Lee, S. H. & Wray, N. R. Genetic correlations of polygenic disease traits: from theory to practice. *Nat. Rev. Genet.* (2019). doi:10.1038/s41576-019-0137-z
3. Chang, C. C. *et al.* Second-generation PLINK: rising to the challenge of larger and richer datasets. *Gigascience* **4**, 7 (2015).
4. Burgess, S., Butterworth, A. & Thompson, S. G. Mendelian Randomization Analysis With Multiple Genetic Variants Using Summarized Data. *Genet. Epidemiol.* **37**, 658–665 (2013).
5. Bowden, J., Davey Smith, G. & Burgess, S. Mendelian randomization with invalid instruments: effect estimation and bias detection through Egger regression. *Int. J. Epidemiol.* **44**, 512–525 (2015).
6. Bowden, J., Davey Smith, G., Haycock, P. C. & Burgess, S. Consistent Estimation in Mendelian Randomization with Some Invalid Instruments Using a Weighted Median Estimator. *Genet. Epidemiol.* **40**, 304–314 (2016).
7. Verbanck, M., Chen, C.-Y., Neale, B. & Do, R. Detection of widespread horizontal pleiotropy in causal relationships inferred from Mendelian randomization between complex traits and diseases. *Nat. Genet.* **50**, 693–698 (2018).
8. O'Connor, L. J. & Price, A. L. Distinguishing genetic correlation from causation across 52 diseases and complex traits. *Nat. Genet.* **50**, 1728–1734 (2018).
9. Wu, Y. *et al.* Genome-wide association study of medication-use and associated disease in the UK Biobank. *Nat. Commun.* **10**, 1891 (2019).
10. Santos, R. *et al.* A comprehensive map of molecular drug targets. *Nat Rev Drug Discov* **16**, 19–34 (2017).
11. Loh, P.-R., Kichaev, G., Gazal, S., Schoech, A. P. & Price, A. L. Mixed-model association for biobank-scale datasets. *Nat. Genet.* **50**, 906–908 (2018).
12. MacArthur, J. *et al.* The new NHGRI-EBI Catalog of published genome-wide association studies (GWAS Catalog). *Nucleic Acids Res.* **45**, D896–D901 (2017).
13. Jiang, W. & Yu, W. Power estimation and sample size determination for replication studies of genome-wide association studies. *BMC Genomics* **17**, 19–32 (2016).
14. Bulik-Sullivan, B. K. *et al.* LD Score regression distinguishes confounding from polygenicity in genome-wide association studies. *Nat. Genet.* **47**, 291–295 (2015).
15. Bulik-Sullivan, B. *et al.* An atlas of genetic correlations across human diseases and traits. *Nat Genet* **47**, 1236–1241 (2015).
16. Finucane, H. K. *et al.* Partitioning heritability by functional annotation using genome-wide association summary statistics. *Nat. Genet.* **47**, 1228 (2015).
17. Finucane, H. K. *et al.* Heritability enrichment of specifically expressed genes identifies disease-relevant tissues and cell types. *Nat. Genet.* **50**, 621–629 (2018).
18. Zhu, Z. *et al.* Integration of summary data from GWAS and eQTL studies predicts complex trait gene targets. *Nat. Genet.* **48**, 481–487 (2016).
19. McRae, A. F. *et al.* Identification of 55,000 Replicated DNA Methylation QTL. *Sci. Rep.* **8**, 17605 (2018).
20. de Leeuw, C. A., Mooij, J. M., Heskes, T. & Posthuma, D. MAGMA: Generalized Gene-Set Analysis of GWAS Data. *PLOS Comput. Biol.* **11**, e1004219 (2015).
21. Cai, N. *et al.* Minimal phenotyping yields GWAS hits of low specificity for major depression. *bioRxiv* 440735 (2019). doi:10.1101/440735
22. Nakazawa, M. Package 'fmsb'. *https://cran.r-project.org/web/packages/fmsb/index.html* (2019).
23. Zhu, Z. *et al.* Causal associations between risk factors and common diseases inferred from GWAS summary data. *Nat. Commun.* **9**, 224 (2018).
24. Wray, N. R. *et al.* Genome-wide association analyses identify 44 risk variants and refine the genetic architecture of major depression. *Nat Genet* **50**, 668–681 (2018).
25. GTEx Consortium. The Genotype-Tissue Expression (GTEx) pilot analysis: Multitissue gene regulation in humans. *Science (80-.).* **348**, 648–660 (2015).
