# Supplementary material for: GWAS of peptic ulcer disease implicates Helicobacter pylori infection, other gastrointestinal disorders and depression
Source: Nat Commun. 2021 Feb 19;12:1146. doi: 10.1038/s41467-021-21280-7 (PMC7895976; doi:10.1038/s41467-021-21280-7)
Supplement: Supplementary file 2 — Description of Additional Supplementary Files [file 41467_2021_21280_MOESM2_ESM.pdf]

Description of Additional Supplementary Files

File Name: Supplementary Data 1.

Description: Detailed pleiotropy analysis for UKB digestion phenotypes associated SNPs using GWAS Catalog Data.

File Name: Supplementary Data 2.

Description: Results of tissue specific enrichment in 54 GTEx v8 tissue using GENE2FUNC of FUMA pipeline (https://fuma.ctglab.nl/), with the input of *MUC1*, *MUC6*, *FUT2*, *PSCA*, *ABO*, *CDX2*, *GAST* and *CCKBR*.

File Name: Supplementary Data 3.

Description: Genetic correlation between each of the five digestion phenotypes and 258 traits from LD hub.

File Name: Supplementary Data 4.

Description: Genetic correlation between each of the four sensitivity analyses phenotypes and 258 traits from LD hub.

File Name: Supplementary Data 5.

Description: Results of gene-based association analyses using MAGMA.
