# Supplementary material for: GWAS of peptic ulcer disease implicates Helicobacter pylori infection, other gastrointestinal disorders and depression
Source: Nat Commun. 2021 Feb 19;12:1146. doi: 10.1038/s41467-021-21280-7 (PMC7895976; doi:10.1038/s41467-021-21280-7)
Supplement: Supplementary file 8 — Reporting Summary [file 41467_2021_21280_MOESM8_ESM.pdf]

nature research

Corresponding author(s): Yeda Wu & Naomi R. Wray

Last updated by author(s): Oct 02, 2020

Reporting Summary

Nature Research wishes to improve the reproducibility of the work that we publish. This form provides structure for consistency and transparency in reporting. For further information on Nature Research policies, see our Editorial Policies and the Editorial Policy Checklist.

Statistics

For all statistical analyses, confirm that the following items are present in the figure legend, table legend, main text, or Methods section.

n/a	Confirmed	
☐	☒	The exact sample size (n) for each experimental group/condition, given as a discrete number and unit of measurement
☐	☒	A statement on whether measurements were taken from distinct samples or whether the same sample was measured repeatedly
☐	☒	The statistical test(s) used AND whether they are one- or two-sided<br>*Only common tests should be described solely by name; describe more complex techniques in the Methods section.*
☐	☒	A description of all covariates tested
☐	☒	A description of any assumptions or corrections, such as tests of normality and adjustment for multiple comparisons
☐	☒	A full description of the statistical parameters including central tendency (e.g. means) or other basic estimates (e.g. regression coefficient) AND variation (e.g. standard deviation) or associated estimates of uncertainty (e.g. confidence intervals)
☐	☒	For null hypothesis testing, the test statistic (e.g. F, t, r) with confidence intervals, effect sizes, degrees of freedom and P value noted<br>*Give P values as exact values whenever suitable.*
☒	☐	For Bayesian analysis, information on the choice of priors and Markov chain Monte Carlo settings
☒	☐	For hierarchical and complex designs, identification of the appropriate level for tests and full reporting of outcomes
☐	☒	Estimates of effect sizes (e.g. Cohen's d, Pearson's r), indicating how they were calculated

Our web collection on statistics for biologists contains articles on many of the points above.

Software and code

Policy information about availability of computer code

Data collection	This is fully described in the Methods and Supplementary Materials (Supplementary Note, Tables, Figures and Data).
Data analysis	Genomic analysis was conducted with BOLT-LMM, LD score regression, MAGMA, GSMR, LocusZoom stand-alone package for data visualization. R 3.4.0 base functions and TwoSample MR for Mendelian Randomization.

For manuscripts utilizing custom algorithms or software that are central to the research but not yet described in published literature, software must be made available to editors and reviewers. We strongly encourage code deposition in a community repository (e.g. GitHub). See the Nature Research guidelines for submitting code & software for further information.

Data

Policy information about availability of data

All manuscripts must include a data availability statement. This statement should provide the following information, where applicable:

- Accession codes, unique identifiers, or web links for publicly available datasets
- A list of figures that have associated raw data
- A description of any restrictions on data availability

Summary statistics are available at http://cnsgenomics.com/data.html. The data that support the findings of this study are available from UK Biobank (http://www.ukbiobank.ac.uk/about-biobank-uk/). Restrictions apply to the availability of these data, which were used under license for the current study (ID: 12505). Data are available for bona fide researchers upon application to the UK Biobank. We also used peptic ulcer disease GWAS summary statistics (https://cnsgenomics.com/data.html) from the Resource for the Genetic Epidemiology Research on Adult Health and Aging (GERA: dbGaP phs000674.v2.p2) study. We used GWAS summary statistics for major depression that include data from 23andMe. These data can be obtained by qualified researchers under an agreement with 23andMe that protects the privacy of the 23andMe participant 23andMe . Researchers can perform meta-analysis of 23andMe summary statistics and the other five-cohort results file, as described in Wray et al.49, to get major depression GWAS summary statistics (excluding UK Biobank cohort). The data for generating the figures are provided

in the Supplementary Materials.

Field-specific reporting

Please select the one below that is the best fit for your research. If you are not sure, read the appropriate sections before making your selection.

- [x] Life sciences
- [ ] Behavioural & social sciences
- [ ] Ecological, evolutionary & environmental sciences

For a reference copy of the document with all sections, see nature.com/documents/nr-reporting-summary-flat.pdf

Life sciences study design

All studies must disclose on these points even when the disclosure is negative.

Sample size	The main analyses were based on 456,327 European individuals with genotype data and phenotype information
Data exclusions	Individuals that were excluded from analysis were based on poor genetic information or other phenotype information and is fully described in the detail in Online Methods section.
Replication	Primary GWAS results were obtained from a single large homogeneous population analysis in UK Biobank. For peptic ulcer and irritable bowel syndrome GWAS significant hits, we used Genetic Epidemiology Research on Aging (GERA) cohort of which the sample size is 61,847 individuals. Given the power setting, we compared the effect size of these GWAS hits in UK Biobank with GERA and the effect size showed similar results.
Randomization	Association analysis was performed on all individuals together adjusting for age, sex, and genetic principle components.
Blinding	Polygenic risk scores calculated are blinded to the phenotype status of participants.

Reporting for specific materials, systems and methods

We require information from authors about some types of materials, experimental systems and methods used in many studies. Here, indicate whether each material, system or method listed is relevant to your study. If you are not sure if a list item applies to your research, read the appropriate section before selecting a response.

Materials & experimental systems

n/a	Involved in the study	
[x]	[ ]	Antibodies
[x]	[ ]	Eukaryotic cell lines
[x]	[ ]	Palaeontology and archaeology
[x]	[ ]	Animals and other organisms
[ ]	[x]	Human research participants
[x]	[ ]	Clinical data
[x]	[ ]	Dual use research of concern

Methods

n/a	Involved in the study	
[x]	[ ]	ChIP-seq
[x]	[ ]	Flow cytometry
[x]	[ ]	MRI-based neuroimaging

Human research participants

Policy information about studies involving human research participants

Population characteristics	The UK Biobank is a longitudinal study, containing genotype and phenotype data of over 400,000 individuals at the time of study recruitment. Detailed information are fully described in Online Method section.
Recruitment	The UK Biobank recruitment aimed for generalizable population and is subject to healthy participant bias in which the sample tends to have reduced disease rates and higher socioeconomic status than a true population sample.
Ethics oversight	This study makes use of data from UK Biobank (Project ID: 12505). UK Biobank is approved by the National Research Ethics Service Committee and participants signed written informed consent, specifically applicable to health-related research. All ethical regulation were followed.

Note that full information on the approval of the study protocol must also be provided in the manuscript.
